# Genome-wide association analysis reveals specialization to hosts and niches in multiple species of the *Lactobacillaceae*

**DOI:** 10.1093/ismejo/wrag198

**Published:** 2026-07-31

**Authors:** Dalimil Bujdoš, Jens Walter, Paul W O’Toole

**Affiliations:** APC Microbiome Ireland, University College Cork, National University of Ireland, Cork, T12 K8AF, Ireland; School of Microbiology, University College Cork, National University of Ireland, Cork, T12 K8AF, Ireland; APC Microbiome Ireland, University College Cork, National University of Ireland, Cork, T12 K8AF, Ireland; School of Microbiology, University College Cork, National University of Ireland, Cork, T12 K8AF, Ireland; Department of Medicine, University College Cork, National University of Ireland, Cork, T12 K8AF, Ireland; APC Microbiome Ireland, University College Cork, National University of Ireland, Cork, T12 K8AF, Ireland; School of Microbiology, University College Cork, National University of Ireland, Cork, T12 K8AF, Ireland

**Keywords:** microbial GWAS, host adaptation, host specialization, Lactobacillaceae, *Limosilactobacillus reuteri*, *Lactobacillus crispatus*, Limosilactobacillus mucosae, *Ligilactobacillus salivarius*, *Ligilactobacillus ruminis*, adhesion

## Abstract

The *Lactobacillaceae* inhabit diverse environments, but the extent of their habitat adaptation remains unclear and the colonization factors unknown. First, we applied multiple machine learning models to determine if we can distinguish strains of the same species isolated from two different habitats based on their gene content. Surprisingly, we show that no species is differentially adapted to the oral cavity versus the human gut, or food versus the human gut, while only *Lactobacillus crispatus* showed specialization to the human urogenital system versus human gut. We then asked which species of *Lactobacillaceae* are habitat-specialized and how they could be identified. Using multiple lifestyle predictors incorporated in logistic regression models, we found that *Limosilactobacillus reuteri, Ligilactobacillus ruminis, L. salivarius, L. crispatus*, and *L. mucosae* displayed the highest degrees of host specialization. Applying our microbial genome-wide association study tool, *aurora*, to these species identified genes encoding adhesins and bacteriocins as the strongest and most common adaptation factors. This work establishes a generalizable framework for identifying novel species–habitat pairs with strong evidence of specialization and for uncovering the genomic features underlying within-species host and habitat adaptation.

## Introduction

Strains belonging to the family *Lactobacillaceae* have been isolated from mammalian and insect gastrointestinal tracts, dairy products, fermented foods such as kimchi, sauerkraut, and sausages, fermented fish or cheese, vegetables, fruits, wastewater, the phyllosphere, and alcoholic beverages such as wine or beer [1–3]. Individual strains within a species are often found in different environments, raising the question of whether they have evolved adaptive mechanisms that restrict them to these habitats or if they engage in more complex lifestyles where different habitats serve as temporary niches for transmission or refuge [1]. To simplify the lifestyle discussion, species belonging to the family *Lactobacillaceae* are usually divided into three categories: host-adapted (vertebrate and insect), environmental (free-living), and nomadic [1, 3, 4]. However, this classification is not based on a statistical method that systematically validates lifestyle and niche associations [1]. This has practical implications, as it is often unclear whether lactobacilli found, for example, in fecal samples can stably colonize the human gut without continuous seeding from food, oral cavity, or probiotics. Whereas some species are commonly isolated from the human gastrointestinal tract (GIT) (specifically *L. gasseri, L. paragasseri, L. ruminis, L. casei, L. paracasei, L. lactis, L. delbrueckii, L. rhamnosus, L. salivarius*), some of these species are also prevalent in food or the oral cavity [5–7].

The degree to which lactobacilli can engraft within the resident human gut microbiota is highly dependent on both species and strain, and may also vary between individuals [8, 9]. Some studies have indicated that species not typically found in the mammalian gut can undergo a period of rapid mutation and achieve stable host colonization [10, 11]. In contrast, other studies suggest that long-term host colonization can only occur over extended evolutionary time-scales [12–14]. In a previous study, we identified early signs of speciation towards multiple hosts in *Salmonella enterica* subsp. Typhimurium [15]. A similar trend was observed in earlier work using a genome-wide association studies (GWAS) independent method [16]. We were also able to distinguish the host-adapted *Limosilactobacillus reuteri* from a generalist *Lactiplantibacillus plantarum* in the same study [15]. We had already validated our approach for identifying microbes in the early and late stages of host specialization; hence, we were interested in whether it was possible to detect signs of specialization to the mammalian gut in food-borne lactobacilli. We argue that if a species can colonize the human gut without requiring continuous replenishment by transmission from the oral cavity or by food containing that microbe, then gut isolates should have distinguishable gene content from oral cavity isolates or food isolates. Multiple species of *Lactobacillaceae* have also been isolated from both the gut and female UGS (urogenital system) [5–7], and strain-level analyses in the same individuals demonstrate rectal–vaginal strain overlap [17, 18]. In contrast, controlled human studies indicate that orally administered lactobacilli do not reliably establish stable colonization of the urogenital tract in healthy women [19, 20]. These contradictory observations motivate comparative genomics to test whether gut-derived strains differ in gene content from strains isolated from the UGS.

Extensive research has explored the interactions of a few reference strains with their habitats [21, 22]. However, the extent to which findings from reference strains can be generalized to the entire species remains uncertain, as different strains usually have distinct gene content and therefore different functional traits. A broader comparative approach, incorporating multiple strains from diverse environments, is therefore necessary to achieve a complete understanding of species-level habitat adaptation strategies. Recently, GWAS techniques have been applied by us and others to elucidate the adaptation strategies of *Lactobacillaceae* [4, 15, 23, 24]. However, applying GWASs on strains that are not adapted to the habitat in which they were isolated (allochthonous species) or on a generalist/nomadic species (species adapted to survive in various habitats) is likely to lead to false-positive results, as it has been shown that various GWAS tools often produce false positives even in the absence of causal genomic factors [15, 25].

The objective of this study was to determine whether strains to identify within-species and environment specific colonization factors. To this end, we assembled a comprehensive database of isolate genomes from the family *Lactobacillaceae* with detailed metadata, encompassing 40 species and nearly 10 000 genomes. Using this database, we demonstrate that food and oral cavity isolates are indistinguishable from human gut isolates and that *L. crispatus* is the only species with separate adaptation factors for the human gut and urogenital system. Because we show that strains isolated from different habitats cannot be distinguished in many species, we first sought to classify species of *Lactobacillaceae* as habitat-specialized or nonspecialized. Among the specialists, we selected those with strong signatures of within-species specialization to their isolation habitats. Subsequently, *aurora* was used to identify putative genetic factors associated with these habitats. To independently validate our GWAS findings, we leveraged the growing availability of metagenome-assembled genomes (MAGs) and employed a newly developed pangenome visualization technique that depicts the co-localization of genes within pangenomes (see Supplementary Methods). Using these analysis pipelines, we identified a number of adaptation factors in multiple species–habitat pairs and we demonstrate that (i) if a species is habitat-specialized, then strains from the same habitat will form distinct lineages in the phylogenetic tree, (ii) adaptation factors to the same habitat differ across species, and (iii) the strongest and most commonly observed adaptation factors are proteins involved in adhesion and bacteriocin production.

## Materials and methods

### Database construction

All genomes were downloaded from NCBI Genome database or Sequence Read Archive (SRA) database between October 2024 and January 2025. Taxonomic classification was verified for all genomes using GTDB-Tk version 2.3.2 [26]. The cutoff for genome completeness was >85% and <15% for contamination as calculated by CheckM2 version 1.1.0 [27]. If reads from SRA were downloaded, then the genomes were assembled by SPAdes version 4.2.0 [28]. Critically, each genome in the database was annotated with its isolation habitat, based on information primarily obtained from the NCBI BioSample and BioProject databases. When these sources lacked habitat data or provided ambiguous information, manual curation was performed to determine the isolation source by searching dedicated databases such as Leibniz Institute DSMZ, BacDive [29], or JGI GOLD [30]. Additionally, the geographic location (country and city/state/county/province) where the strain was isolated was also recorded in the database. This resource is deposited at Zenodo repository (DOI 10.5281/zenodo.17582651).

### Identification of habitat specialization

To find out if a species has evolved specific adaptation factors towards two environments (e.g. food vs gut) we developed a method similar to that used in our previous study [15]. The method assumes that if a species is differentially adapted to two habitats, then it should be possible to distinguish strains from these two habitats based on gene content. In this analysis, first one strain from each habitat was mislabelled into the other habitat, then this resulting dataset was adjusted for population structure using a phylogenetic walk algorithm [15]. Then, a Classification And Regression Tree (CART) model was built to classify the strains into the two categories. This process was repeated multiple times (the number of repetitions was equal to the number of strains in the category with fewer strains). Lastly, if the mislabelled strains have significantly lower classification probabilities than the nonmislabelled strains (*P* < .05, Kolmogorov–Smirnov test), then it was assumed that the two categories are distinguishable and thus the species is specifically adapted to these two habitats.

### Calculation of pangenomic fluidity, residualized standardized residualized effect sizes, and prediction of macromolecular systems

Pangenomic fluidity was calculated as described before [31]. This value represents the average of ratios of unique genes to the sum of all genes in a pair of two randomly selected genomes.


\begin{align*} pangenome\ fluidity=\frac{2}{N\left(N-1\right)}\sum_{k,l=1\dots N}\frac{U_k+{U}_l}{M_k+{M}_l} \end{align*}

where *U_k_* and *U_l_* are the number of genes found only in genomes *k* and *l*, respectively, and *M_k_* and *M_l_* are the total number of genes found in *k* and *l*, respectively. It was shown that pangenomic fluidity is stabilized after *N≥ 20* [31], which is lower than the minimal number of strains in our database. However, the value of pangenomic fluidity can be influenced by population structure (e.g. if we introduce multiple clonal strains the value would decrease). The population-adjusted pangenomic fluidity was calculated using the same equation, but the *k* and *l* genomes were not selected randomly but using a phylogenetic walk algorithm [15].

The standardized residualized effect sizes (SESs) for each species describe how much strains isolated from each host cluster on the phylogenetic tree. The greater the value, the more the strains from each host form separate lineages. First, a parsimony score is calculated for each species–tree pair using the Fitch algorithm [32]. Then, the habitat labels were randomized on the same phylogenetic tree and the parsimony score was calculated again. This randomization step was repeated 1000 times to create a null distribution of parsimony scores. SES is the number of standard deviations from the mean of the null distribution to the observed parsimony score. However, the SES was positively correlated with the number of strains in the dataset (*r* = 0.67, *P* < .01, Fig. S5). To account for the correlation between the number of strains and the SES, we residualized the SES with respect to the number of strains by fitting a linear model (SES ~ number of strains) and extracting the residuals.

The mobility of each species was assessed using Mac-Sy-Finder [33] version 2.1.4 and the TXSScan model [34]. If at least 75% of species’ genomes were found to encode a flagellum structure, then the whole species was labelled as mobile. Other macromolecular systems such as CRISPR-Cas systems were identified with the same tool with additional models: TFFscan [35], CasFinder [36], and CONJScan [37].

### Pangenome and phylogenetic tree construction, GWAS analysis, and gene annotation

The pangenomes and phylogenetic tree were constructed as described before [15]. Briefly, the annotated genomes of each species were used as an input into Panaroo version 1.2.9 [38]. Then, the phylogenetic tree was constructed by first aligning protein sequences of all single-copy core genes using MAFFT version 7.490 [39]. The alignments were trimmed by trimAI version 1.4.15 [40]. These alignments served as an input into IQ-TREE2 version 2.2.0 [41] with 1000 bootstrap replicates using UFBoot2 [42]. The tree was rooted by midpoint rooting. The GWAS analyses were carried out by *aurora* [15]. First, the function aurora_pheno was run with panaroo pangenome and the phylogenetic tree as an input with default parameters except repeats = 10. Then the function aurora_GWAS was run with default parameters except use_ada = FALSE, use_log = FALSE. Then, the top 100 adaptation factors associated with each host were annotated. The annotation pipeline is available here: (link to an online tool will be inserted upon manuscript acceptance). Each adaptation factor was annotated using three approaches: KofamScan [43], InterProScan version 5.76 [44], and UniRef90 [45]. In addition to the GWAS analysis, we employed two GWAS-independent approaches to validate the colonization factors identified by GWAS. The first approach is based on the co-localization of genes for colonization factors within the genomes of habitat-specialized strains, whereas the second leverages MAGs, that is, genomes not included in the GWAS analysis. These two methods are described in detail in Supplementary Methods. MAGs for verification of the GWAS results were downloaded from SPIRE [46]. The cutoff for completeness was >50% and for contamination <10%.

## Results and discussion

### Database of currently available genomes of the *Lactobacillaceae*

**Figure 1:**
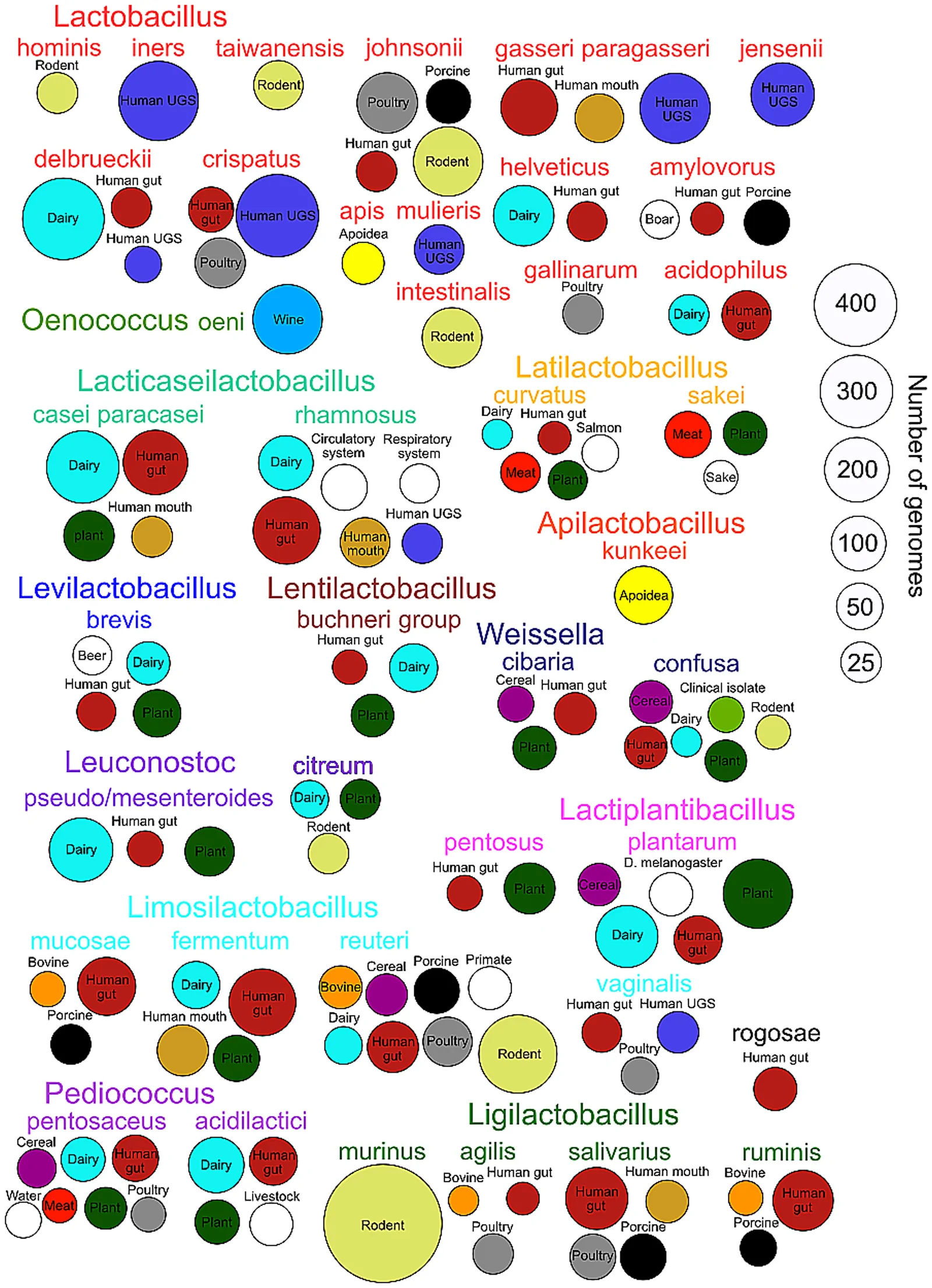
Graphical representation of the database of genomes from the family *Lactobacillaceae* constructed from sequences downloaded from the NCBI assembly database and the SRA database. The isolation habitats are color-coded, with rare isolation habitats having no color. In order to maximize power in the GWAS analysis, some species were grouped (e.g. *L. gasseri/paragasseri*, buchneri group) so the power of the analysis is increased urogenital system (UGS), buchneri group = *L. buchneri, L. parabuchneri, L. kefiri, L. parakefiri, L. sunkii*, and *L. otakiensis*.

We constructed a comprehensive database of genomes of the members of the family *Lactobacillaceae*. There are nearly 300 species in this family [47, 48], but to allow for statistically meaningful comparisons, we restricted the analysis to species represented by >20 genome sequences in each habitat where the species was isolated. This resulted in a final dataset of 40 species (Fig. 1). After these filtering steps, the database comprised 9983 genome sequences with 23 unique isolation habitats. The most populous habitats were rodents (2185 genomes), followed by the human gut (1877), dairy environments (1739), and the human urogenital system (UGS) (1475).

Because of the variety of habitats from which lactobacilli are isolated, we deployed a low level of granularity for habitat annotation. For instance, the ‘dairy’ category was assigned regardless of specific sources such as cheese, milk, and yogurt. Likewise, all edible plant sources, including fermented foods like kimchi and sauerkraut, were grouped under a single label, ‘plants’. Whereas fermented beverages such as beer, wine, and sake could also be categorized under a single label, we chose to classify them separately due to the specific adaptation mechanisms required for survival in high-ethanol environments [49]. The most frequently represented species in the current dataset was *L. murinus*, with 1266 genome sequences. Whereas the majority of these are rodent isolates originating from a single BioProject (PRJEB59335), the isolates themselves were collected from diverse geographic locations [50]. To maximize the power to detect adaptation factors, we grouped some phylogenetically related species into single analytical categories. These new categories were *Lactobacillus gasseri* grouped with *Lactobacillus paragasseri, Lacticaseibacillus casei* with *Lacticaseibacillus paracasei, Leuconostoc mesenteroides* with *Leuconostoc pseudomeseteroides* and a *buchneri* group comprising *Lentilactobacillus buchneri, Lentilactobacillus parabuchneri, Lentilactobacillus kefiri, Lentilactobacillus parakefiri, Lentilactobacillus sunkii*, and *Lentilactobacillus otakiensis*.

### Food isolates are indistinguishable from human gut isolates and food lactobacilli are not in the process of speciation towards the human gut

We argue that if certain food-associated lactobacilli were adapted to humans or other mammals, or were in the process of so adapting, then the gene content of their genomes should reflect this. In other words, we should be able to find genes that distinguish human isolates from strains isolated from other habitats. For this analysis, we examined 15 species of the *Lactobacillaceae* for which sufficient numbers of genome sequences were available (Fig. S1) from strains isolated both from food sources and from the human gut. We applied a Classification And Regression Tree (CART) model to these species to distinguish the food isolates and human gut isolates. Before the CART model was used, one strain from both habitats was mislabelled to the other habitat. Multiple CART models with mislabelled strains were then built. If strains isolated from two habitats are specialized, the classification probabilities of mislabelled strains should be significantly lower than those of correctly labelled strains (see Materials and Methods). We ran this in parallel with our previously described *aurora* [15].

**Figure 2:**
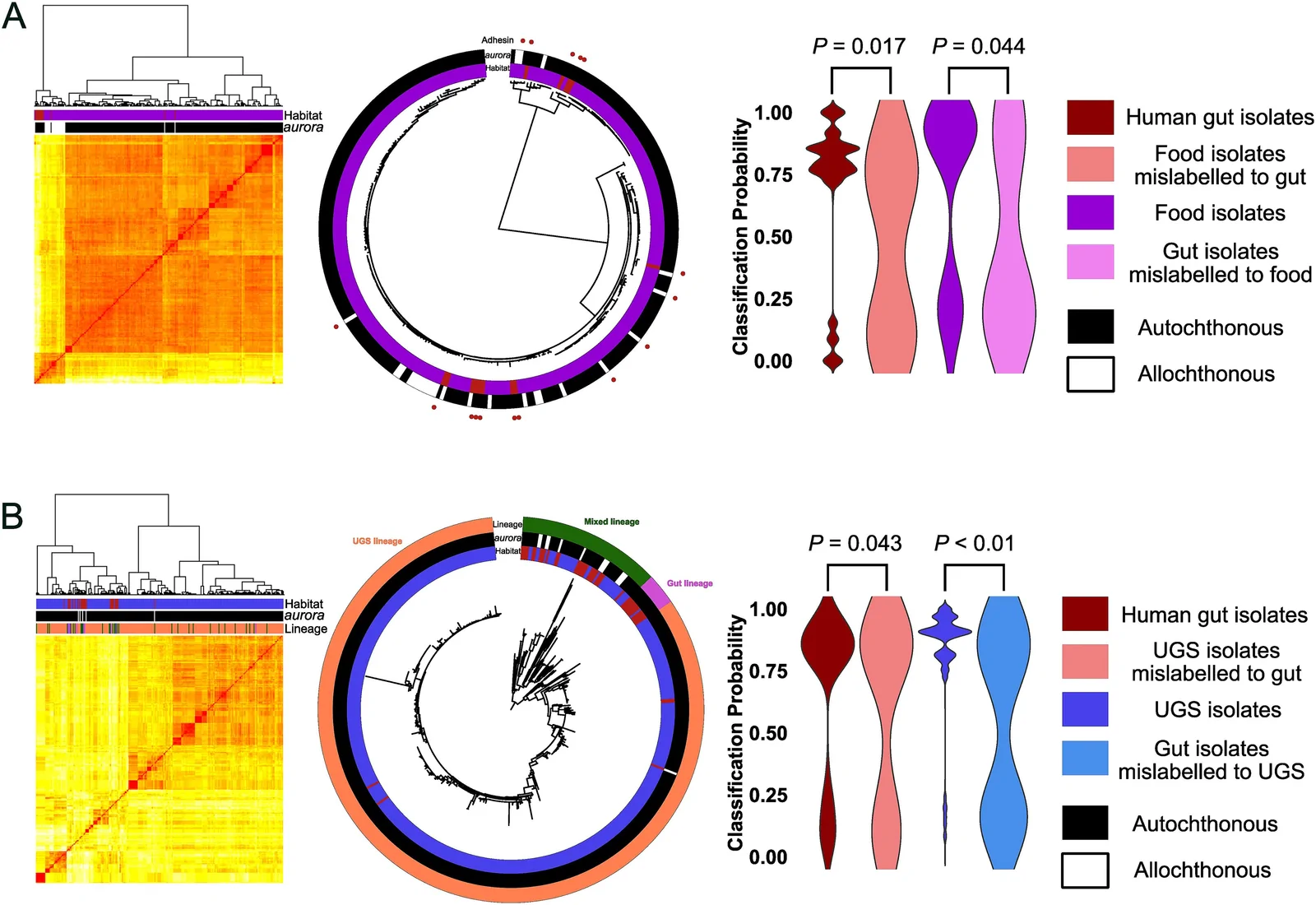
Comparative analysis of *L. mesenteroides/pseudomesenteroides* strains isolated from food and human gut (A) and *L. crispatus* isolated from human urogenital system (UGS) and human gut (B). These were the only two species whose isolates from these habitats could be distinguished based on gene contents. The heatmaps in each panel show the similarity between strains based on how often they are grouped together by a random forest model used in *aurora*. Strains that are frequently classified together appear closer and cluster in the heatmap, indicating the presence of the same habitat colonization factors. The core-genome phylogenetic trees show how the strains from different hosts (inner ring) cluster, and the outer ring shows if the strains were predicted as either autochthonous (true stable colonizer; black band) or allochthonous (introduced from a different habitat; white band). In panel (A), an additional ring shows the presence of a human gut–specific adhesin gene. In panel (B), the additional band shows the three lineages with different adaptation pattern. The violin plots show results of the CART model analysis.

This analysis showed that, for 14 species in the *Lactobacillaceae*, food and human gut isolates from the same species were indistinguishable based on gene content (Fig. S1). Thus, these species are unlikely to be specialized to the human gut. The only group where the CART model was able to distinguish between human gut isolates and food isolates was *Leuconostoc pseudomesenteroides/mesenteroides* (Fig. 2A and Fig. S1). Mislabelled food and gut isolates of this species exhibited significantly lower classification probabilities in the CART model (Fig. 2A) consistent with their clustering in the heatmap (Fig. S1). Only 11 human gut isolate genomes were available for *L. pseudomesenteroides/mesenteroides*, and these samples come from only six studies. Therefore, a batch effect could be responsible for the difference and that more isolates are necessary to support the evidence for specialization. Next, we carried out an *aurora* analysis to identify the human gut adaptation factors of *L. pseudomesenteroides/mesenteroides*. The top-ranked human gut colonization factor differentiated the gut isolates from the food isolates well. This gene was present in all human gut isolates and present in only 4% of food isolates and was annotated as an LPXTG-motif containing surface protein by UniProt (accession number: A0A3P1VBF3). Thus, this gene is a potential human-specific adhesin. As we show below, adhesins are usually adaptation factors with the highest effect size and appear to be essential for colonization of many habitats. Other top human gut colonization factors had much lower effect size and are thus unlikely to contribute to colonization of human gut by *L. pseudomesenteroides/mesenteroides*. The *aurora* analysis shows that the dairy and plant isolates are distinguishable based on gene content and clustered on the phylogenetic tree (Fig. S11). In the Supplementary Materials, we discuss the adaptation factors to these two habitats.

### Human oral cavity isolates are indistinguishable from human gut isolates

Several species of the *Lactobacillaceae* are commonly found at multiple human body sites, including the oral cavity, gastrointestinal tract, and vagina [1, 5, 6]. A central question is whether the same bacterial strains can colonize both the oral cavity and the gut, or whether distinct, niche-adapted lineages of the same species occur in each location. Some studies suggest that many lactobacilli detected in the human gut represent transient allochthonous microbes originating from the oral cavity, rather than stable gut residents [51–53]. However, not all studies agree on the extent of oral–gut overlap. A more recent investigation concluded that the salivary and colonic microbiotas of healthy adults were distinct, with no evidence of oral bacterial colonization of the distal gut under healthy conditions [54]. In contrast, ’oralization‘ of the gut microbiome is a feature of several disease states including colon cancer [55].

To test these competing hypotheses, we applied the CART-based analysis to genomes of seven species found in both the human oral cavity and gut (*L. fermentum, L. rhamnosus, L. salivarius, L. casei/paracasei,* and *L. gasseri/paragasseri*). This analysis could not distinguish oral isolates from gut strains based on genomic content for any of these species (Fig. S2), indicating that lactobacilli are not differentially adapted to these two habitats. It has been proposed that the oral cavity is the primary habitat of these species and that oral strains are introduced into the gut through swallowing of saliva [56]. These data also suggest that probiotic candidates intended to improve oral health may not need to be oral isolates. For example, *L. salivarius* AR809, isolated from the oral cavity and reported to adhere to pharyngeal mucosa and reduce inflammation [57], may not possess unique oral-adaptation traits that the gut strains lack. Hence, other *L. salivarius* strains, even gut isolates, might achieve similar effects.

### *Lactobacillus crispatus* is the only tested species with differing adaptation to human urogenital system and human gastrointestinal system

The human intestine and vagina represent different ecosystems. The gut provides a nutrient-rich (but low in simple sugars), near-neutral pH environment with high microbial diversity and intense interspecies competition in which lactobacilli are present in low numbers of relative proportions of the microbial community. Instead, the vagina is characterized by lower diversity, acidic pH (~3.5–4.5), glycogen-derived nutrients, and dominance of species from the *Lactobacillaceae*. Our genomic dataset contained six species (*L. delbrueckii, Limosilactobacillus vaginalis, L. rhamnosus, L. gasseri/paragasseri,* and *L. crispatus*) that occur in both the gastrointestinal tract and the female urogenital tract. In addition, *L. delbrueckii* and *L. rhamnosus* can also be found in food (Fig. 1). Previous comparative genomics and GWASs provide some evidence for strain specialization to one or other of these two niches [58–60]. Using our CART-based approach to assess habitat specialization, only *L. crispatus* was identified as being genomically specialized to the gut and vaginal environments (Fig. S3 and Fig. 2B). Gut isolates mislabelled as being from the UGS showed significantly lower classification probability for belonging to that niche. In the phylogenetic tree, three lineages can be distinguished: a large UGS-only lineage, a smaller mixed lineage, and a small gut-only lineage (Fig. 2B). The strains from the gut-only lineage are also often clustered together on a heatmap generated by *aurora*, whereas the strains from the mixed lineage are spread around the heatmap (Fig. 2B). Even though certain strains or lineages of other *Lactobacillus* species may display higher relative fitness in one environment (e.g. [58, 59], our data support a scenario in which gut and vaginal isolates of some species such as *L. crispatus* can successfully transition between habitats or remain adapted to either. This is consistent with earlier studies reporting that rectal and gut isolates are often identical to vaginal isolates and that orally administered probiotics can pass through the gastrointestinal (GI) tract and reach the vagina [61, 62].

### Identification of strong within-species habitat specialization

In the analyses presented in the preceding three paragraphs (Fig. 2), we show that, for most of the species analysed, gene content does not allow reliable discrimination between strains isolated from two different habitats. Consequently, applying a computationally and manually intensive GWAS to all 40 species of *Lactobacillaceae* would be impractical and may lead to many false positives. We therefore sought to determine whether general genomic features could predict the degree of habitat specialization across species, therefore enabling us to focus GWAS analyses on the most highly habitat-specialized taxa. As we show above and as has been discussed previously [1, 3], many species in the *Lactobacillaceae* are not specialized to any particular habitat. Of 40 species examined in a previous phylogenomic study [1], 8 had no source/habitat labels and 12 were from a single habitat, making them already classifiable as specialists, leaving 20 species that we considered for the analysis. These 12 species isolated from a single habitat were removed from the analysis as they were not an object of our interest because the goal of this study was to identify within-species adaptation factors that can be only identified if the species has been isolated from more than one habitat. We also wanted to quantify the degree of specialization in the remaining strains and therefore searched for additional GWAS-independent measures of the degree of specialization within species. A previous study suggested that pangenomic fluidity, genome size, and motility are key predictors of bacterial lifestyle [63]. Accordingly, we calculated the average genome size (approximated by the number of genes per genome) and inferred motility based on the presence of flagellar genes in at least 75% of the strains within a species. We also estimated pangenomic fluidity for each species, a metric that quantifies the proportion of genes shared between pairs of randomly selected strains [31]. It has previously been shown that specialists generally have lower pangenomic fluidity (i.e. more conserved gene content) than generalists [63].

**Figure 3:**
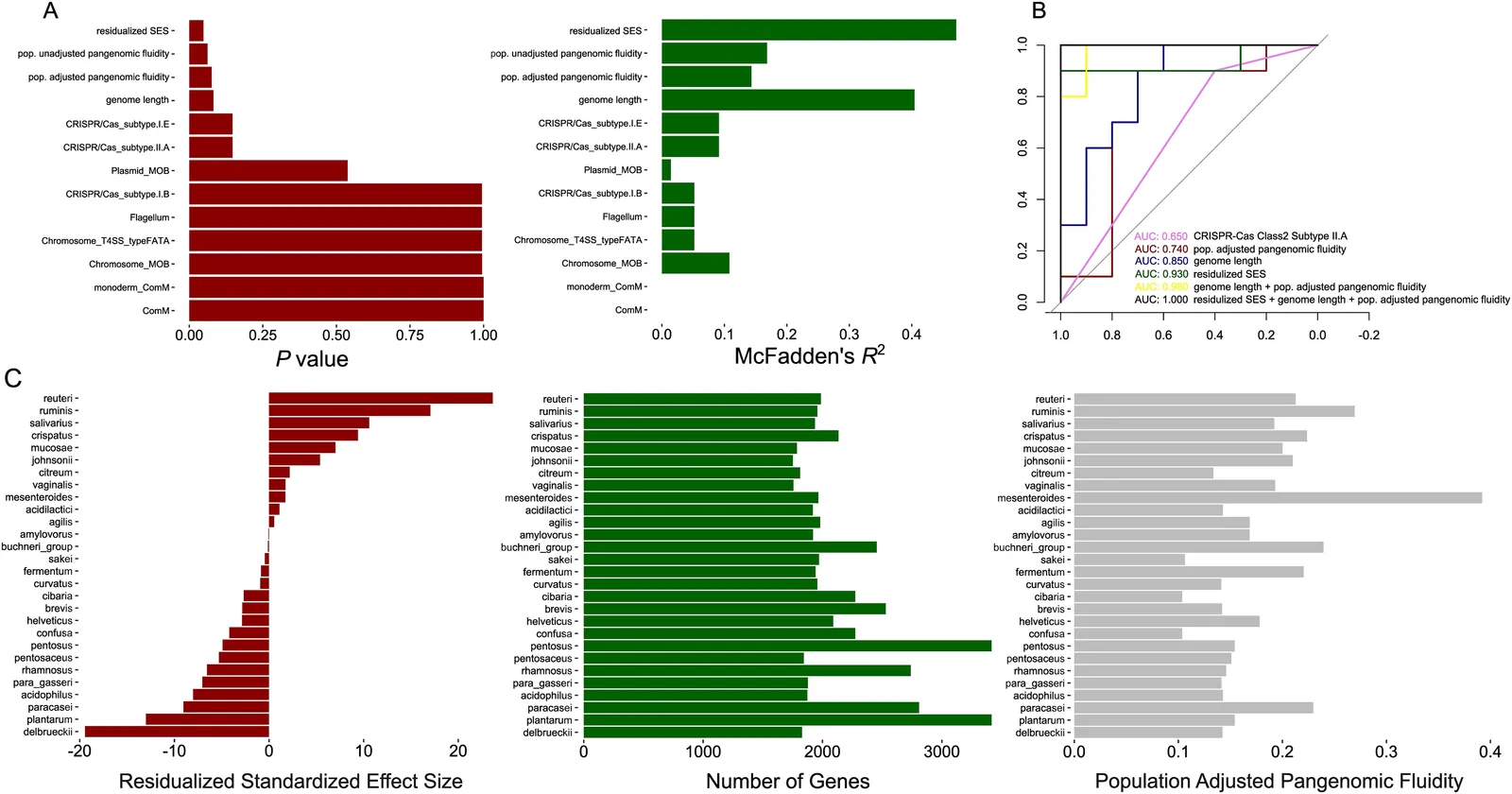
Results from the logistic regression analysis. (A) *P*-values and McFadden’s *R*^2^ values (effect size of the feature) calculated for logistic regression models with only one generalist/specialist predictor (rows). (B) AUC curves for six logistic regression models. (C) the values of the top three predictors used in the best logistic regression model. The species are sorted based on residualized standardized effect sizes which was the best predictor of specialist/generalist label.

To assess the predictive power of each variable in the generalist/specialist classifier, we constructed separate logistic regression models and calculated both the *P*-values and McFadden’s *R*^2^ to evaluate model performance relative to random chance. A good predictive variable would have a low *P*-value, indicating statistical significance, and a high McFadden’s *R*^2^, indicating a large effect size. As expected, flagellum production was not a significant predictor (Fig. 3A; *P* = .99), consistent with the fact that only one motile species in the dataset, *L. ruminis*, has been previously identified [64]. Similarly, pangenomic fluidity showed limited predictive value, with a *P*-value of .062 and a McFadden’s *R*^2^ = 0.17 (Fig. 3A). In contrast, genome size proved to be a strong predictor, yielding a *P*-value of .08 and a McFadden’s *R*^2^ of 0.40 (Fig. 3A). The specialist–generalist prediction model based on genome size alone achieved an accuracy of 80% and an area under curve (AUC) of 0.85 (Fig. 3B). We hypothesized that the lack of predictive power of pangenome fluidity may stem from confounding effects due to population structure. In other words, because strains share phylogenetic history—some are even clonal, effectively representing repeated observations—they cannot be treated as independent observations, as is implicitly assumed in standard pangenome fluidity calculations. This issue was partially addressed in a previous study where Markov chain Monte Carlo generalized linear mixed models were used to account for phylogenetic structure at the species level [63]. However, we argue that this approach is insufficient, as the strongest confounding arises at the strain level. To mitigate this, we employed an algorithm we previously developed, phylogenetic walk, which simulates random genome sampling based on a phylogenetic tree [15]. Using this algorithm, we quasi-randomly selected genome pairs to calculate pangenome fluidity. The results, however, remained comparable to the unadjusted analysis (*P* = .076, McFadden’s *R*^2^ = 0.14, Fig. 3A). Nonetheless, incorporating this adjusted fluidity metric into a logistic regression model with genome size improved predictive performance (accuracy = 0.90, AUC = 0.98, Fig. 3B).

To further expand the predictive framework, we evaluated additional genomic features, including protein translocation and secretion systems, CRISPR-Cas systems, Tad (tight adherence) pili, Com (competence) pili, and other conjugative or mobilizable elements. These candidates were selected based on prior evidence of ecological relevance. For instance, protein translocation systems have been implicated in host adaptation in *L. reuteri* [65]. CRISPR-Cas systems are more prevalent in environments with high viral abundance, and the type of CRISPR-Cas system is also predictive of the environmental type [66]. Conjugative and mobilizable elements are also known to facilitate lateral gene transfer in microbiomes with high microbial density [67]. Despite their biological plausibility, none of these additional predictors significantly improved model performance. The most promising, CRISPR-Cas class II-A, yielded a *P*-value of .15 and McFadden’s *R*^2^ of 0.091 (Fig. 3A). Adding this predictor to the existing model with genome length and population-structure-adjusted pangenome fluidity did not enhance overall accuracy (Fig. 3B). We also conducted the same analysis at the strain level rather than the species level (Fig. S4). Consistent with our previous findings, genome length remained the strongest predictor, yielding a *P*-value <.001 and a McFadden’s *R*^2^ of 0.68.

To further improve model performance, we explored additional variables that could better capture lifestyle-specific signatures. Previous studies showed that mammal-adapted, insect-adapted, and food-adapted species of the *Lactobacillaceae* are often group together on a phylogenetic tree [3, 68]. Similarly, rodent- and poultry-adapted strains of *L. reuteri* are also found in only a few phylogenetically distinct lineages [69, 70]. This pattern is consistent with expectations: it has been proposed that host association evolves gradually over long evolutionary timescales, beginning with a free-living lifestyle, passing through an intermediate phase, and eventually becoming fully host-associated [12–14]. We hypothesized that incorporating a variable quantifying the degree to which habitat labels group on phylogenetic trees could further enhance the predictive power of our model. We thus developed a measure that quantified this clustering: residualized standardized effect size (residualized SES).

This value proved to be a stronger predictor of generalist versus specialist classification than either genome length or pangenome fluidity (*P* = .048, McFadden’s *R*^2^ = 0.471, Fig. 3A). The corresponding model achieved 90% accuracy and an AUC of 0.93 (Fig. 3B). When genome length and population-adjusted pangenome fluidity were added to the model, performance improved further, achieving an AUC of 1.0 and 100% classification accuracy (Fig. 3B). We propose that the residualized SES serves as a quantitative measure of a species’ degree of specialization to the diverse habitats from which it has been isolated. In line with this interpretation, species such as *L. reuteri, L. ruminis, L. salivarius, L. crispatus*, and *L. mucosae* exhibit the highest within-species specialization and were therefore selected as taxa for further genome content analysis in this study. With the exception of *L. reuteri*, these species are exclusively isolated from host-associated environments, including human, porcine, bovine, rodent, and poultry (Fig. 1).

### Habitat adaptation features of selected species

In this section, we present the results of GWAS analyses carried out by *aurora* [15]. In addition to *aurora*, a method leveraging the growing availability of MAGs was developed to verify *aurora* results independently of GWAS assumptions. Additionally, a new pangenome visualization method was devised that assisted us in identification of host-specific gene groups. These two methods are compatible with the *aurora* output and are introduced in the Supplementary Materials. The finalized plots depicting the analysis of the five species are available here (link to an online tool will be inserted upon acceptance), and we deposited all databases, associated annotation files, and *aurora* results separately (DOI 10.5281/zenodo.17582651). Lastly, a detailed discussion of the adaptation factors identified across these five host-specialized species is provided in the Supplementary Materials.

*Limosilactobacillus reuteri*: According to our multiphasic regression analysis (Fig. 3C) *L. reuteri* is the most host-specialized species among the tested *Lactobacillaceae*. This species is frequently found in the gastrointestinal tract of mammals [56], and our dataset comprised sufficient strain numbers (>20) for GWAS analysis from six hosts: rodents, poultry, humans, nonhuman primates, cows, and pigs. Rodent specialization is the strongest as evidenced by the high-standardized residuals of the top adaptation genes predicted by *aurora* (see Supplementary Data) and by the high proportion of these top adaptation factors that passed the MAGs verification test (36 out of 100 top adaptation factors), meaning that the prevalence of these genes was also significantly higher in MAGs derived from rodent metagenomes (see Supplementary Results). This is concordant with previous reports that rodent is the original host of *L. reuteri* and that the species has developed multiple adaptation mechanisms to survive in this host [15, 69]. Using co-localization mapping, we observed that the genes encoding rodent adaptation factors are localized in a few distinct genetic groups (Fig. S6). The urease operon (*ureABCDEFG*) along with the ammonium transporter *ngrA* and cobalt/nickel transporter (LarMNOQ) represented one of the largest groups. The urease operon is known to be essential for rodent colonization [71] and the products of *ngrA* in conjunction with LarMNOQ facilitate its function (see Supplementary Materials for discussion). The highest-ranking rodent colonization factor predicted by *aurora* was o-succinylbenzoate synthase (*menC*), an enzyme involved in menaquinone (vitamin K₂) biosynthesis. This gene appears to be exclusive to rodent-adapted *L. reuteri* isolates (Supplementary Data). Supplementation with menaquinone and heme has previously been shown to improve growth efficiency and stress resistance in lactic acid bacteria, indicating that *menC* may confer an adaptive advantage in the rodent gut [72, 73].

In contrast to rodent-associated adaptation, the *L. reuteri* adaptation to other hosts appears to be weaker and less well characterized. This likely reflects both the limited number of studies on these hosts and the weaker association of *L. reuteri* with them, which may have resulted in fewer host-specific adaptation factors [65, 69]. We identified multiple genes in poultry isolates associated with vitamin synthesis: pantothenic acid (vitamin B5), folate (vitamin B9), and cobalamin (vitamin B12). Cobalamin specifically is essential for reuterin synthesis; reuterin is a potent antibacterial compound that inhibits the growth of bacteria and fungi [74].

**Figure 4:**
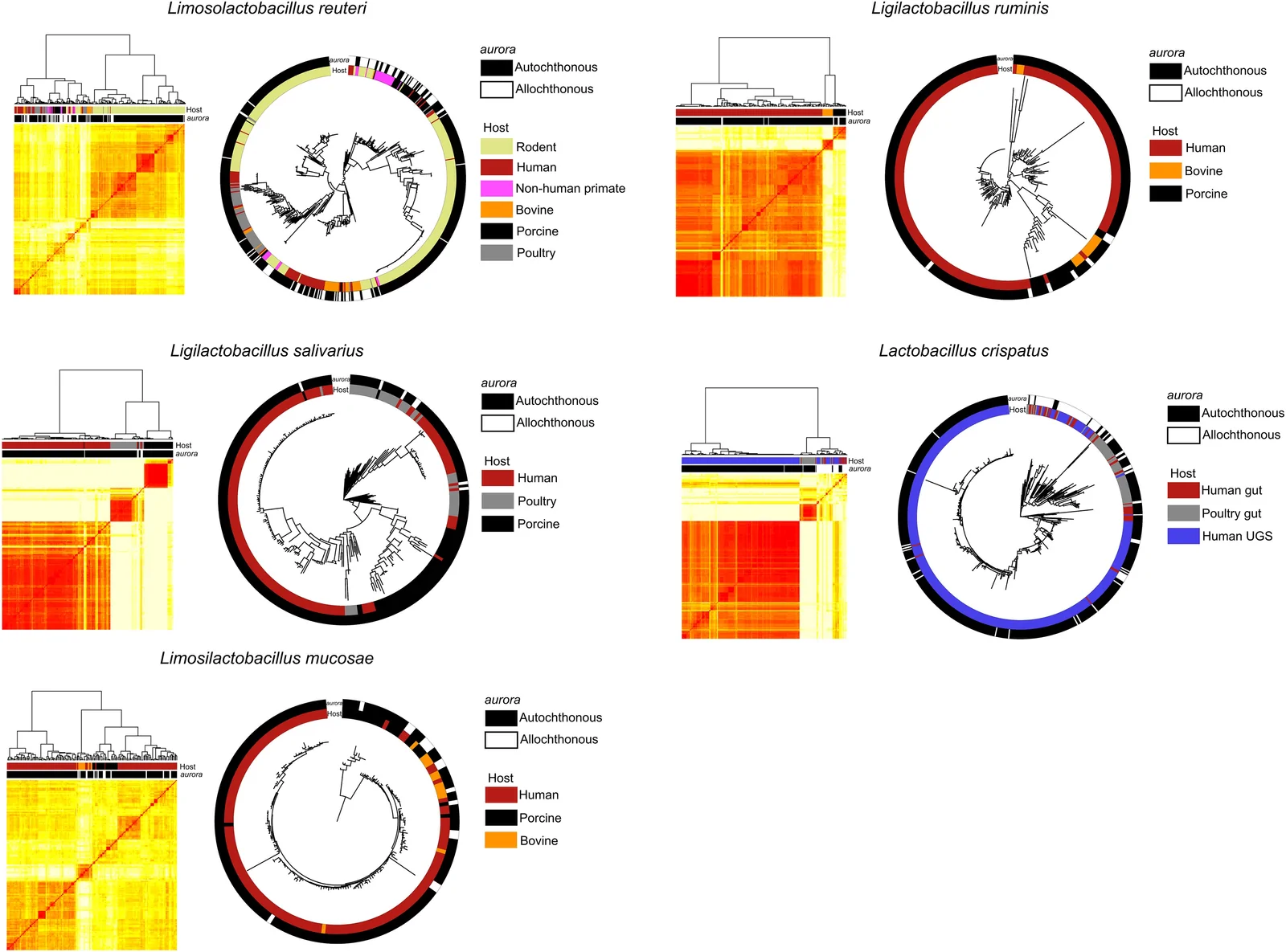
*aurora* analysis of five host-specialized species of *Lactobacillaceae*. The heatmaps in each panel show the similarity between strains based on how often they are grouped together by a random forest model used in *aurora*. Strains that are frequently classified together appear closer and cluster in the heatmap, indicating the presence of similar habitat colonization factors. The core-genome phylogenetic trees show how the strains from different hosts cluster (inner ring) and if the strains were predicted as either autochthonous (true stable colonizer) or allochthonous (introduced from a different habitat).

We and others have previously shown that human isolates of *L. reuteri* are difficult to distinguish from rodent and poultry adapted isolates, and human isolates do not seem to have greater genomic adaptation to the human gut compared to isolates from other hosts [15, 75]. However, in the current study, we show that these strains can be distinguished by *aurora* (Fig. 4). Among them are two LPXTG cell wall–anchored proteins (putative adhesins), and histidine decarboxylase (HdcA)/histidine decarboxylase maturation protein (HdcB). HdcA/HdcB produce histamine from histidine—a possible probiotic trait. Histamine produced by gut-residing bacteria can influence immune responses via the human histamine receptor 2, possibly exerting anti-inflammatory effects [76, 77].

*Ligilactobacillus ruminis*, the species showing the second highest degree of host-specialization, was isolated from human, porcine, and bovine gastrointestinal tract. Isolates from these three hosts can be clearly distinguished (Fig. 4, *aurora* heatmap and phylogenetic tree) indicating long-term host-specific adaptation. Carbohydrate utilization has been reported to be the most distinguishing trait in *L. ruminis*, with lactose and sucrose utilization uniquely associated with human isolates [78]. Our results concur, but in addition, we were able to identify human-associated carbohydrate utilization genes with a lower effect size. These include genes for transport or catabolism of mannose, ribose, glucosides, cellobiose (demonstrated before [79]), xylobiose, and xylose. Bovine isolates did not harbour such an extensive repertoire of host-associated carbohydrate utilization factors (see Supplementary Data), and genes related to microbial competition dominated the top-ranking bovine adaptation factors. These include genes for restriction endonucleases, CRISPR-Cas systems, bacteriocins, bacteriocin immunity proteins, and bacteriocin transporters.

Porcine adaptation in *L. ruminis* appears to be a comparatively recent event relative to human or bovine adaptation (Fig. 4). Our GWAS analysis identified only 16 genes within the top 100 adaptation factors that also passed validation in the MAGs test (Supplementary Data). Of these, 10 showed a high effect size, being present in at least 70% of porcine strains and absent from isolates of other origins. However, 9 of these 10 genes were annotated as hypothetical proteins. Beyond these high-effect genes, we also detected several with lower effect sizes. Among these, genes implicated in iron and copper acquisition and resistance, as well as β-1,6-galactofuranosyltransferase and α-1,6-mannosyltransferase involved in exopolysaccharide biosynthesis at the cell surface, were particularly noteworthy.

*Ligilactobacillus salivarius* shows clear evidence of genomic adaptation to human, poultry, and porcine gastrointestinal systems (Fig. 4). The host adaptation of this species has been extensively studied because of its probiotic effects in humans [80–82]. The high-effect size human colonization factors identified by *aurora* [exopolysaccharide (EPS) synthesis and CRISPR-Cas systems] are already well known [81]. However, in addition to these we identified a number of yet-unknown human adaptation factors such as the bacillosamine biosynthesis cluster. This cluster is involved in synthesis of diacetylbacillosamine-containing glycans. These glycans serve as a protein glycosylation scaffold and facilitate host interaction and immune evasion [83]. Another example is a methionine acquisition cluster or a regulatory switch composed of diguanylate cyclase and a c-di-GMP phosphodiesterase. These two genes control the transition between biofilm-bound and dispersed state [84], which is intriguing for an oral/gut species known to form biofilms [85].

Porcine isolates of *L. salivarius* are all localized in one phylogenetic lineage that diverged from the human-adapted lineage (Fig. 4). The top-ranked colonization factors were annotated as either phage-related genes or transposases. Other high-effect-size genes encode SecA2-SecY2 protein translocation system or EPS-related genes (see below for further discussion). The low-effect-size genes encode, for example, utilization of rhamnose and fucose. Of note, this pattern of high-effect size genes encoding mobile genetic elements was likewise evident in GWAS results from hosts with which *L. reuteri* has only recently established adaptation (e.g. bovine and porcine). Host specialization is thought to initiate through transient interactions with the host, during which bacterial genomes experience genomic decay while incorporating new genes [12–14]. It is thus possible that mobile genetic elements are the first genes that are acquired during host adaptation. These are then followed by genes that confer actual fitness advantage, whose acquisition is facilitated by the mobile genetic elements.

Because some of the porcine isolates are genomically closely related to the poultry strains (Fig. 4), the overall adaptation profile of *L. salivarius* in poultry resembles that observed in porcine isolates. In both groups, the adaptation gene set is largely composed of glycosyltransferases, EPS biosynthesis enzymes, and components of the SecA2-SecY2 protein translocation system. Certain adaptation factors, such as SecY2, are shared between porcine and poultry isolates whereas others such as L-lactate oxidase or sialidase are poultry-specific. L-lactate oxygenase generates bacteriostatic H_2_O_2_ from lactic acid and sialidase removed sialic acid residues from host produced mucins.

*Lactobacillus crispatus* is primarily isolated from the human urogenital tract and from poultry, including chickens and turkeys. Although it can also be recovered from the human gastrointestinal tract, it is considered an uncommon inhabitant in this environment [6]. As was demonstrated above (Fig. 2) and further supported by the *aurora* analysis across different host species (Fig. 4), a distinct *L. crispatus* lineage adapted to the human gastrointestinal tract was identified. This lineage possessed multiple colonization factors including genes for degradation of host-derived mucins (β-glucuronidase, β-N-acetylhexosaminidase, and β-glucoside catabolism), genes for utilization of dietary carbon sources sugars (ribose, glycerol, xylan, cellobiose, galactosides, glucosides, and rhamnose), CRISPR-Cas, and a complete purine biosynthesis operon.

In addition to this gut-adapted lineage, we also observed lineages associated with poultry, the human urogenital system, and a mixed-origin lineage comprising strains from multiple habitats (Fig. 4). Due to its strong association with vaginal health, several *L. crispatus* strains have been developed as probiotics with the aim of promoting women’s health [86].

The *aurora* statistics (Supplementary Data) indicate that pullulanase is one of the most important human UGS colonization factors in *L. crispatus*. This gene product enables degradation of host glycogen into maltodextrins that are further fermented to lactic acid [87]. We also identified cysteine desulfurase and several Suf system proteins that assemble and repair Fe–S clusters under oxidative stress. These functions are essential in the human UGS where hydrogen peroxide, much of it produced by *L. crispatus* itself, creates oxidative stress. By preserving Fe–S cofactors, the Suf pathway supports metabolic activity and has previously been proposed as a colonization factor in *L. crispatus* (Selbach *et al*., 2014; Zhang *et al*., 2020d). In addition to the pullulanase and Suf systems, multiple adhesins were also identified: S-layer proteins, fibronectin-binding proteins, and homolog of staphylococcal clumping factor B (see Supp. Data). A dedicated surface display machinery was also present among the top adaptation factors.

*Limosilactobacillus mucosae* is isolated from human, porcine, and bovine gastrointestinal systems, but compared with other species analysed here, it appears to harbour fewer high- effect-size host-adaptation factors. A notable human gut colonization factor is a complete tryptophan biosynthesis operon. Possession of this pathway enables *L. mucosae* to produce tryptophan independently, a trait that is uncommon among human gut microbes and may provide a competitive advantage [88]. Beyond serving as a nutrient, microbial transformation of tryptophan generates metabolites that shape host physiology and mediate cross-talk within the gut microbiota [89]. Human gut isolates of *L. mucosae* have some adaptation factors that are similar to those in *L. crispatus* strains adapted to the human UGS, such as ascorbate catabolic genes (ascorbate phosfotranferase (PTS) system co-localized with a transketolase gene) and a 1,4-α-glucan branching enzyme (also annotated as neopullulanase) involved in glycogen catabolism.

### Summary of adaptation factors in *Lactobacillaceae*

Multiple host-specialized species analysed here have the apparent ability to colonize the same hosts, namely, human, poultry, rodent, bovine, porcine, and nonhuman primates. We proposed two hypotheses: (i) the presence of a shared set of host adaptation factors across species, or (ii) host adaptation being species-specific, which would reflect distinct ecological niches within the same host. Our results largely support the latter hypothesis. With a few exceptions, the adaptation factors identified were not shared across different species colonizing the same host (see Table 1 for overview of adaptation factors). Those exceptions are tetracycline resistance genes (*tetW* in *L. reuteri* adapted to porcine, *tetM* in *L. salivarius* adapted to porcine and in *L. crispatus* adapted to poultry). Tetracyclines and penicillins are among the most frequently used antibiotics in pig farming [90]. However, tetracyclines has been banned for use in porcine husbandry in European Union countries since 2019 (https://eur-lex.europa.eu/legal-content/EN/TXT/?uri=CELEX:32019R0004). It would therefore be interesting to investigate if these genes are equally spread in isolates from the European Union compared to other countries. We also identified a gene associated with resistance to penicillin (penicillin amidase) that was present in porcine-adapted and human-adapted *L. ruminis* strains. In addition, two polyether ionophore transporters in poultry-adapted *L. crispatus* strains were identified. Ionophore antibiotics such as salinomycin and narasin are used in poultry farming as a coccidiostats [91].

**Table 1:** Selected adaptation factors identified in host-adapted species of *Lactobacillaceae*.

Adaptation factor	Species	Hosts	Protein or gene
Tetracycline resistance	*Limosilactobacillus reuteri*	Porcine	Tetracycline resistance ribosomal protection protein TetW
	*Ligilactobacillus salivarius*	Porcine	Tetracycline resistance ribosomal protection protein TetM
	*Lactobacillus crispatus*	Poultry	Tetracycline resistance ribosomal protection protein TetM
CRISPR-Cas systems	*Ligilactobacillus salivarius*	Human gut	Multiple CRISPR-Cas gene clusters
	*Lactobacillus crispatus*	Human gut	Multiple CRISPR-Cas gene clusters
	*Limosilactobacillus mucosae*	Human gut	Multiple CRISPR-Cas gene clusters
	*Ligilactobacillus ruminis*	Bovine	Multiple CRISPR-Cas gene clusters
Antimicrobial compound production and resistance	*Limosilactobacillus reuteri*	Poultry Porcine	*pdu-cbi-cob-hem* genomic island (reuterin pathway)
	*Ligilactobacillus ruminis*	Human gut	Linearmycin/Streptolysin S transport permease
	*Ligilactobacillus ruminis*	Bovine	Multiple bacteriocin synthesis and immunity gene clusters
	*Limosilactobacillus mucosae*	Human gut	Linearmycin/Streptolysin S transport permease
	*Lactobacillus crispatus*	Poultry	Two polyether ionophore transporters (salinomycin and narasin resistance)
	*Ligilactobacillus salivarius*	Human gut	Nisin
	*Ligilactobacillus salivarius*	Porcine	Bacteriocin gene and associated transporter
	*Ligilactobacillus ruminis*	Porcine Human gut	Penicillin amidase (penicillin resistance)
Adhesins	*Limosilactobacillus reuteri*	Rodent	Serine-rich repeat adhesin
	*Ligilactobacillus salivarius*	Porcine	Fibronectin-binding protein/mucin adhesion protein
	*Lactobacillus crispatus*	Poultry	Fibronectin-binding protein A and LPXTG motif protein
	*Limosilactobacillus reuteri*	Rodent	Multiple mucin-binding genes
	*Limosilactobacillus reuteri*	Human gut	Two LPXTG motif proteins
	*Ligilactobacillus ruminis*	Porcine	Fibronectin-binding protein
	*Ligilactobacillus salivarius*	Porcine	Collagen-like protein
	*Ligilactobacillus salivarius*	Human gut	Cell wall–anchored WxL-domain protein
	*Lactobacillus crispatus*	Human UGS	Two fibronectin-binding proteins
	*Lactobacillus crispatus*	Poultry	Mucin-binding protein and generic adhesin gene
	*Lactobacillus crispatus*	Human UGS, Poultry	Multiple S-layer proteins
Teichoic acid and cell wall modifications	*Limosilactobacillus reuteri*	Human gut	Teichoic acid glycerol-phosphate transferase, TuaB, TuaG
	*Ligilactobacillus ruminis*	Bovine	Phosphocholine metabolism genes (modifying teichoic acid)
	*Ligilactobacillus salivarius*	Porcine	Teichoic acid D-alanyltransferase
	*Lactobacillus crispatus*	Human gut	D-alanine-D-alanine ligase, teichoic acid D-alanine hydrolase
	*Limosilactobacillus mucosae*	Human gut	Alanine-adding enzyme
Exopolysaccharide (EPS) production	*Limosilactobacillus reuteri*	Human gut Poultry Porcine	Partial EPS synthesis gene cluster
	*Ligilactobacillus salivarius*	Human gut Porcine	Partial EPS synthesis gene cluster
	*Limosilactobacillus mucosae*	Human gut Porcine	Partial EPS synthesis gene cluster
	*Ligilactobacillus ruminis*	Bovine	Partial EPS synthesis gene cluster
Acid stress resistance	*Limosilactobacillus reuteri*	Rodent	Urease operon and cobalt/nickel transport system
	*Limosilactobacillus reuteri*	Rodent	Glutaminase and glutamate/GABA antiporter
	*Limosilactobacillus reuteri*	Poultry	Formyl/oxalate CoA-transferase
	*Ligilactobacillus ruminis*	Bovine	Acetoin permease
	*Limosilactobacillus mucosae*	Porcine	Acetoin permease
	*Ligilactobacillus ruminis*	Bovine	Arginine deiminase
	*Ligilactobacillus salivarius*	Porcine	Ornithine decarboxylase
	*Lactobacillus crispatus*	Human UGS	Chloride channel and sodium/hydrogen exchange protein
Bile salt hydrolase	*Lactobacillus crispatus*	Human gut	Bile salt hydrolase/chologlycine hydrolase
	*Ligilactobacillus salivarius*	Porcine	Bile salt hydrolase/chologlycine hydrolase
Arsenate resistance	*Limosilactobacillus reuteri*	Rodent	Arsenate reductase
Copper resistance	*Ligilactobacillus ruminis*	Porcine	Two copper-binding proteins
	*Lactobacillus crispatus*	Poultry	Copper-exporting ATPase
Tannin detoxification	*Limosilactobacillus reuteri*	Nonhuman primates	Hypothetical protein with an LpdD-like domain, flavin prenyltransferase, gallate decarboxylase, and a transcriptional regulator
Carbon utilization	*Ligilactobacillus ruminis*	Human	Mannose/sorbose PTS genes
	*Limosilactobacillus reuteri*	Nonhuman primates	Uronic acid utilization genes
	*Lactobacillus crispatus*	Human UGS	*ula* operon (ascorbate utilization)
	*Ligilactobacillus salivarius*	Poultry	Sialidase

We also show that the presence of CRISPR-Cas systems is an indicator that a species is a human-specific colonizer. We identified multiple CRISPR-Cas systems in human-adapted strains of *L. salivarius, L. crispatus,* and *L. mucosae*. In addition to those, we identified a gene cluster with seven CRISPR-Cas genes in bovine adapted *L. ruminis*. CRISPR-Cas systems are more common in habitats with high competition [66, 92], and they are often lost and acquired [92]. However, it is not clear why CRISPR-Cas systems are adaptation factors for strains in the human gut and not in other hosts. It has been shown that low phage diversity and high phage abundance favours the presence of CRISPR-Cas systems [66]. It is thus possible that the human gut has uniquely high abundance but low diversity of phages. Different animals have very different diversity of phages. Yu *et al*. identified 977 viral OTUs in mice, 12 896 in pigs, and 1480 in cynomolgus macaques [93]. However, no comparable numbers are available for humans.

The most common adaptation factor was antimicrobial compound production (e.g. bacteriocins, antibiotics), which was identified in every species analysed. For example, we identified reuterin production in poultry and pig strains of *L. reuteri* as an adaptation factor. This is consistent with the previous literature that shows that the *pdu–cbi–cob–hem* genomic island is responsible for reuterin production and is present in poultry and pig isolates but infrequent in rodent isolates [94, 95]. Unlike the CRISPR-Cas system, bacteriocin production was not specifically associated with any host and appears to be a universal mammalian colonization factor. The importance of bacteriocin production also appears to be variable. Some species such as bovine-adapted *L. ruminis* strains harbour multiple gene clusters related to bacteriocin synthesis and immunity, whereas porcine adapted strains of *L. salivarius* have only one gene (of high effect size) encoding a bacteriocin and a co-localized bacteriocin transporter. It would therefore be interesting to see if a specific type is associated with a specific host. However, annotation of bacteriocin genes is still difficult because of the large sequence diversity and a low number of experimentally annotated sequences [96].

In addition to bacteriocins, adhesins have also been identified as colonization factors in all species analysed here. One of the best characterized adhesins is a serine-rich repeat protein specific to rodent isolates of *L. reuteri* [65, 97–99]. We also identified this protein as a rodent colonization factors (mucin-binding protein, group_1363). We show that this adhesin gene is co-localized with genes encoding an O-glycosylation system and SecA2–SecY2 protein translocation system (link to an online tool will be inserted upon acceptance). This pair of systems was identified by *aurora* as a colonization factor in other host–species combinations: for example, poultry-adapted *L. reuteri*. However, in this case, we were not able to identify the gene encoding the ‘target’ adhesin. The pair of systems was also present in porcine and poultry isolates of *L. salivarius*. In porcine isolates, we identified two possible candidates for the dependent adhesin: a fibronectin-binding protein co-localized with the two systems and a large high-effect size mucin adhesion protein (see Supp. Data). Likewise, we were also able to pinpoint the SecA2–SecY2 dependent adhesin in poultry-adapted isolates. One substrate candidate is a fibronectin-binding protein A, and another is an LPXTG motif-containing protein that was the top poultry-associated gene and which is even co-localized with the genes of the SecA2–SecY2 system.

In addition to these adhesins, SecA2–SecY2-independent adhesins were also identified. For example, we identified multiple high effect size mucin-binding genes in rodent-adapted *L. reuteri*. Two large proteins with LPXTG motif were also identified in human-adapted *L. reuteri*. A fibronectin-binding protein and a collagen-like protein were also identified as a high effect size colonization factor in porcine adapted *L. ruminis* and *L. salivarius* respectively. Collagen-like proteins are known to bind to host fibronectin or collagen and can thus serve as adhesins [100]. In addition, we identified a cell wall–anchored protein with a C-terminal WxL domain as a human-specific adaptation factor of *L. salivarius*. WxL-domain proteins were shown to mediate adhesion [101]. Two fibronectin-binding proteins with a high effect size were also identified in *L. crispatus* adapted to the human UGS. Likewise, two high-effect size genes, mucin-binding protein and a gene annotated simply as adhesin, were found to be poultry-specific adaptation factors of *L. crispatus*. Lastly, porcine-adapted *L. mucosae* strains harbour three collagen-like proteins. Genes encoding two of these were co-localized (Supp. Data). We also identified multiple lower-effect size S-layer proteins in human UGS-adapted and poultry-adapted *L. crispatus*. S-layer proteins have been experimentally shown to function as adhesins in *L. crispatus* [102].

However, S-layer proteins and surface adhesins are not the only factors important for adhesion. In every species, we identified teichoic acid modification mechanisms that may enhance adhesion to mucus or cell surfaces but could also make the bacterial cell wall more resistant to the external environment [103]. In human-adapted *L. reuteri* strains, we found a large gene cluster containing teichoic acid glycerol-phosphate transferase and teichuronic acid biosynthesis proteins TuaB and TuaG, as well as multiple genes that potentially further modify the cell wall. In bovine-adapted *L. ruminis* strains, genes involved in phosphocholine metabolism were identified. Phosphocholine-decorated teichoic acids serve as anchoring sites for choline-binding proteins, which function as adhesins in several Gram-positive species [104]. In porcine-adapted *L. salivarius*, we identified teichoic acid D-alanyltransferase as an adaptation factor. This enzyme modifies the bacterial cell wall, a key mechanism for tolerating acidic environments and resisting cationic antimicrobial peptides [105]. Likewise, in human adapted *L. crispatus* strains, two gene clusters containing cell wall biosynthesis genes (e.g. D-alanine–D-alanine ligase or teichoic acid D-alanine hydrolase) were identified as high-ranking features. Lastly, we identified genes in human gut–adapted *L. mucosae* strains that contribute to D-alanylation of teichoic acids—a known colonization factor in rodent gut *L. reuteri* colonizers [105].

Another common trait, particularly in humans and pig strains, was the production of EPS layer. EPS has multiple roles, including transit through the gastrointestinal tract, immune evasion or modulation, mucus adhesion, and biofilm formation [106, 107]. EPS production was identified as a colonization factor in human-adapted *L. reuteri, L. salivarius*, and *L. mucosase* strains; porcine-adapted *L. ruminis, L. salivarius*, and *L. mucosae*; and bovine-adapted *L. ruminis*. However, the regions of the genomes identified by *aurora* did not contain what was expected for a complete EPS synthesis cluster. These clusters are usually large spanning multiple kilobases [108]. We identified only certain genes from these clusters that were host-specific. This is in agreement with a previous study that observed that certain EPS genes are under host-specific selection pressure [81]. EPS clusters are thus not interchangeable and specific genes are needed for colonization of a specific host. Further research should focus on what modifications are essential for colonization of different hosts. Such analysis is beyond the scope of this paper as it would require *k*-mer- or unitig-based GWAS analysis.

The isolates studied here were either from the gastrointestinal tract or from the UGS. In these two environments, the strains are challenged with low pH. During gut transition, the strains are briefly exposed to low pH in the stomach. In the UGS, the strains are continuously living in low pH (~4). In addition to the EPS synthesis and teichoic acid modifications described above, other adaptive mechanisms to combat the effects of low pH were identified. A typical example is the presence of a urease operon in rodent-adapted strains of *L. reuteri* [97]. Urease also requires nickel ions to catalyse the hydrolysis of urea into ammonia subsequently increasing the extracellular pH [71]. In agreement with this, we have also identified a cobalt/nickel transport system that is co-localized with the urease operon in *L. reuteri*. Another adaptation mechanism in the same species is conversion of glutamine to glutamate carried out by glutaminase. The glutamate is then exported out of the cell via glutamate/GABA antiporter. The produced ammonia then buffers the intracellular or extracellular pH [109]. We also identified acid resistance genes in poultry-adapted *L. reuteri*, specifically the formyl/oxalate CoA-transferase. Experimental colonization in rodents has shown that this gene is essential for acid resistance in *L. reuteri* [110].

A predicted acetoin permease in bovine-adapted *L. ruminis* and porcine-adapted *L. mucosae* was identified. Acetoin secretion is a known mechanism to combat cell acidification [111]. In addition to this gene, we also identified arginine deiminase, the first enzyme of the arginine deiminase pathway, as a bovine adaptation factor in *L. ruminis*. This pathway is particularly relevant under acidic conditions, where ammonia helps buffer intracellular pH [112]. In porcine-adapted *L. salivarius* strains, we identified ornithine decarboxylase. This enzyme decarboxylates ornithine to putrescine, a process that consumes protons and contributes to pH homeostasis. This amino acid decarboxylation pathway is a known acid resistance mechanism in *L. rossiae* [113]. In UGS-adapted *L. crispatus* multiple ion transporters (chloride channel and sodium/hydrogen exchange protein) were identified. These genes likely allow *L. crispatus* to survive and grow at ~pH 4 by preventing intracellular acidification [60].

Low pH is not the only stressor that lactobacilli endure. For example, in human-adapted *L. crispatus* and porcine-adapted *L. salivarius*, we identified bile salt hydrolase (or chologlycine hydrolase). The enzyme encoded by this gene detoxifies bile by cleaving conjugated bile acids [114]. In rodent-adapted *L. reuteri*, a gene encoded as arsenate reductase was also identified as colonization factor. This gene is known to render the bacteria able to withstand high concentration of arsenic [115]. Copper resistance genes were also among the host-colonization factors in porcine-adapted *L. ruminis* and poultry-adapted *L. crispatus*. Copper is frequently supplemented as a growth promoter in pig and poultry feed [116, 117]. Lastly a four gene cluster of enzymes capable of detoxifying tannins was identified in *L. reuteri* adapted to nonhuman primates. Tannins are polyphenolic compounds present in primate diet, like fruits and leaves, that inhibit microbial growth.

A number of carbon utilization genes were identified in each species–host combination. These genes had a variable effect size, ranging from low effect size genes like the mannose/sorbose PTS system in human adapted *L. ruminis* strains or uronic acid utilization in nonhuman primate–adapted *L. reuteri* strains, to high effect size genes like the L-ascorbate catabolic *ula* operon in UGS isolates of *L. crispatus* or sialidase in poultry-adapted *L. salivarius*. Sialidase is an enzyme that catalyses the removal of sialic acid from glycoproteins and glycolipids and makes it available for utilization as a carbon source. Different species of *Lactobacillaceae* had different carbohydrate utilization enzymes as adaptation factors to the same host, and we did not observe any unifying pattern per host in carbohydrate utilization profiles. However, our analysis was not targeted at identifying shared adaptation factors across the species. For that type of analysis, we are currently conducting a GWAS analysis at the family level.

## Conclusion

This study provides a comprehensive analysis of host adaptation in representative species of the largest microbial family: *Lactobacillaceae*. We compiled a large genome dataset with detailed metadata, which is available for future research. Using this resource, we addressed key questions regarding habitat association. In most species (except *L. mesenteroides/pseudomesenteroides*), food and human gut isolates were genomically indistinguishable. Hence, probiotic strains of these species are unlikely to stably colonize the human gut. Likewise, human oral and gut isolates showed no clear genomic separation, suggesting that the oral cavity may represent the primary habitat for many species of *Lactobacillaceae*, whereas gut isolates are likely allochthonous. Urogenital isolates were also largely indistinguishable from gut isolates, with *L. crispatus* as the only exception, displaying distinct gut- and urogenital-associated genes. Across the dataset, five species showed the strongest signatures of habitat specialization: *L. reuteri, L. ruminis, L. salivarius, L. crispatus*, and *L. mucosae*. Convergent evolution in these species was limited, indicating that host-adaptive traits are mostly species-specific. Adhesion and microbial competition genes were the most common high-effect size adaptation factors across species and habitats. We acknowledge that this study does not provide a fully exhaustive analysis of habitat adaptation factors within the *Lactobacillaceae*. Owing to limited availability of genomic data for many species, our analyses were restricted to 40 species out of the ~300 currently described in the family. In addition, the coarse granularity of habitat annotations in our database may have limited the detection of adaptation to specific food niches. For example, subdividing the ’dairy‘ category into more specific labels such as cheese and milk could potentially reveal additional, niche-specific adaptation factors. Finally, we employed a pangenome-based GWAS that does not capture sequence-level variation within gene families or variation in core genes. Alternative approaches, such as unitig-based GWAS, may therefore identify additional adaptation factors. However, such methods substantially reduce interpretability, as unitigs are more difficult to annotate and biologically contextualize than gene families.

## Supplementary Material

Supplementary_material_wrag198

## Data Availability

GWAS results and database of *Lactobacillaceae* strains are deposited in a permanent Zenodo repository (DOI 10.5281/zenodo.17582651). Our GWAS results (Figs S6, S7, S8, S9, S10) are also available as an interactive 2D plots for further exploration: https://github.com/DalimilBujdos/Lactobacillaceae_interactive_plots. Lastly, we developed an analysis pipeline for processing and interpretation of GWAS results. This pipeline is maintained and available here: https://github.com/DalimilBujdos/Lactobacillaceae_MAGs_verification.
