# Supplementary material for: Genome-wide association analysis reveals specialization to hosts and niches in multiple species of the Lactobacillaceae
Source: ISME J. 2026 Jul 31;20(1):wrag198. doi: 10.1093/ismejo/wrag198 (PMC13548328; doi:10.1093/ismejo/wrag198)
Supplement: Supplementary file 1 [file supplementary_material_wrag198.zip › supplementary_materials_final.pdf]

Supplementary Figures

Figure S1. Comparative analysis of human gut and food isolates. Violin plots for each species of *Lactobacillaceae* isolated from both food and the human gut. In this analysis, one strain from each habitat was deliberately mislabelled and a CART model was trained on the complete dataset. If the two habitats differ, the classification probabilities of mislabelled strains should be significantly lower than those of correctly labelled strains. This procedure was repeated multiple times, and the resulting distributions are shown as violin plots. Significant differences ($p < 0.05$, Kolmogorov-Smirnov test) between food and human gut strains were observed only for *Leuconostoc mesenteroides/pseudomesenteroides*.

Figure S2. Comparative analysis of strains from human gut and human oral cavity. (A) Violin plots for each Lactobacillaceae species isolated from both human oral cavity and the human gut. In this analysis, one strain from each habitat was deliberately mislabelled and a CART model was trained on the complete dataset. If the two habitats differ, the classification probabilities of mislabelled strains should be significantly lower than those of correctly labelled strains. This procedure was repeated multiple times, and the resulting distributions are shown as violin plots. Significant differences ($p < 0.05$, Kolmogorov-Smirnov test) between oral cavity and human gut strains were not observed in any species. The violin plots are accompanied by a phylogenetic tree showing only oral cavity and human gut isolates.

Figure S3. Comparative analysis of strains from human urogenital system (UGS) and human gut. Violin plots for each *Lactobacillaceae* species isolated from both human UGS and the human gut. In this analysis, one strain from each habitat was deliberately mislabelled and a CART model was trained on the complete dataset. If the two habitats differ, the classification probabilities of mislabelled strains should be significantly lower than those of correctly labelled strains. This procedure was repeated multiple times, and the resulting distributions are shown as violin plots. Significant differences ($p < 0.05$, Kolmogorov-Smirnov test) between human UGS and human gut strains were observed only in *L. crispatus*. The violin plots are accompanied by a phylogenetic tree showing only human UGS and human gut isolates.

Figure S4. Logistic regression analysis of species from the family Lactobacillaceae. P values and McFadden's R2 values (effect size of the feature) calculated for logistic regression models with only one generalist/specialist predictor (rows). Unlike the results in Figure 3A. in the main text, the predictors were not aggregated at the species level, allowing for more predictors to be considered. A separate logistic regression model was constructed for each predictor.

Figure S5. Correlations between Standardized Effect Size (SES) and two variables: Number of strains in each species and number of isolation habitats in each species.

Figure S6. *Limosilactobacillus reuteri* pangenome co-localization plot used for analysis of GWAS results and a pie chart showing the distribution of MAGs across different habitats. The figure is available as an interactive map here: https://github.com/DalimilBujdos/Lactobacillaceae_interactive_plots.

Figure S7. *Ligilactobacillus ruminis* pangenome co-localization plot used for analysis of GWAS results and a pie chart showing the distribution of MAGs across different habitats. The figure is available as an interactive map here: https://github.com/DalimilBujdos/Lactobacillaceae_interactive_plots.

Figure S8. *Ligilactobacillus salivarius* pangenome co-localization plot used for analysis of GWAS results and a pie chart showing the distribution of MAGs across different habitats. The figure is available as an interactive map here: https://github.com/DalimilBujdos/Lactobacillaceae_interactive_plots.

Figure S9. *Lactobacillus crispatus* pangenome co-localization plot used for analysis of GWAS results and a pie chart showing the distribution of MAGs across different habitats. The figure is available as an interactive map here: https://github.com/DalimilBujdos/Lactobacillaceae_interactive_plots.

Figure S10. *Limosilactobacillus crispatus* pangenome co-localization plot used for analysis of GWAS results and a pie chart showing the distribution of MAGs across different habitats. The figure is available as an interactive map here: https://github.com/DalimilBujdos/Lactobacillaceae_interactive_plots.

Figure S11. Results of GWAS analysis of *Leuconostoc mesenteroides/pseudomesenteroides* (A) *aurora* analysis of *L. mesenteroides/pseudomesenteroides* (clustered distance matrix based on random forest distances). The clustering reflects the habitat distribution. (B) Phylogenetic tree of *L. mesenteroides/pseudomesenteroides*. The inner circle indicates habitat, and the outer dots mark the presence or absence of the top candidate habitat adaptation factors.

Supplementary Methods

Two new methods to validate microbial GWAS findings

Previous studies have shown that microbial GWAS results often include many false positives (1,2). This is mainly due to imperfect control of linkage disequilibrium confounding (i.e., some genomic variants are transferred or inherited more often than expected by chance), as well as sampling bias and population structure in microbial datasets. Similar challenges are also present in human GWAS. Results of these analyses may include SNPs that pass the *P value* threshold but are not causal. Typically, only a cluster of SNPs with elevated log(*P* values) is a stronger signal that a genomic region is associated with the phenotype (3). To address these issues, researchers in this more established field developed various methods (4). One common approach is the use of Manhattan plots to visualize GWAS results. These plots help identify loci with consistently high log(*P* values) and they filter out isolated SNPs that passed the significance threshold but are not causal. Following the visualization step, fine-mapping tools are applied to estimate the posterior inclusion probability for each SNP. The outcome of this analysis is a “credible set” of genomic variants that is likely to contain the true causal variant(s). In the current study, we adopted a similar approach. First the pangenome and phylogenetic tree are used as an input into *aurora* (equivalent of human

GWAS). Instead of Manhattan plots we developed a pangenome visualization method that enables the identification of gene clusters associated with specific phenotype/host. In addition, we introduced a GWAS-independent method to provide stronger evidence linking genomic variants to the host (equivalent of fine-mapping).

Our analysis pipeline begins with the collection and annotation of genomes of microbial isolates from relevant databases (NCBI genome or SRA). We then construct a pangenome and generate a core-genome phylogenetic tree, which serves as an input for *aurora*. Next, we compile a separate dataset of the same species, this time using MAGs (metagenome-assembled genomes) instead of isolates. The MAGs dataset contains large numbers of draft genomes assembled into scaffolds from total community sequencing projects. For each gene family in the pangenome (constructed from isolates), we extract a representative sequence and perform a BLASTP search against this MAGs database to identify which genomes contain the gene. Very rare genes (present in fewer than 0.5% of isolates) or core genes (present in at least 95% of isolates) are excluded in this step. Based on the presence/absence pattern of each gene, we compute standardized residuals for each habitat. A higher standardized residual indicates a stronger association between the gene and the habitat (the same association measure is used in *aurora*). The standardized residuals obtained from presence/absence pattern of each gene family in the MAGs dataset are normally distributed. Hence a corresponding z-scores and then P values were calculated. The results are thus P values for every host-gene pair. If a gene family appears among the top 100 adaptation factors identified by *aurora* and its P value from the MAG-based test is below 0.05, we consider this strong evidence of host association. However, as we discuss below this method is reliable only when the number of MAGs is sufficient (>30).

However, even with additional layer of evidence from MAGs, it is difficult to identify the credible set of adaptation factors. One major obstacle is identifying how these adaptation

factors cooperate together to give the species advantage during the host colonization. For example, in our analysis of *Limosilactobacillus reuteri* adaptation to rodents, we identified multiple glycosyltransferases as well as components of the SecA2-SecY2 protein translocation system (see below). SecA2-SecY2 system and the associated glycosyltransferases are known rodent adaptation factors and these genes act on a large surface adhesin (5,6). These results on their own do not reveal which surface protein these gene products target as there are multiple surface proteins in the top rodent colonization factors identified by *aurora*. Notably, with our new pangenome visualization method we showed that in most rodent isolates of *L. reuteri*, the genes encoding the SecA2-SecY2 system and the glycosyltranferases are co-localized with a serine-rich repeat protein gene (Figure S6). This co-localization suggests that these genes are involved in the glycosylation and export of this protein. Indeed, this has been experimentally confirmed in previous studies (7,8).

When *L. reuteri* genes are ranked by their rodent-associated standardized residuals derived from *aurora*, the accessory Sec system protein Asp3 has rank 14, accessory Sec system protein Asp2 has rank 45, SecY2 has rank 52, and the glycosyltransferase GlyD has rank 120. Identifying the functional relationship among these genes would be difficult without knowing whether these genes are co-localized in *L. reuteri* genomes. Co-localization and synteny analysis in bacteria is particularly challenging beyond small sample sizes due to the plasticity of bacterial genomes and the fragmented nature of sequences in public databases. Nevertheless, synteny is often key to understanding gene function, as host- and phenotype-associated genes tend to be co-localized, as demonstrated in the example above. To address this, we developed a robust method which enables visualization of genomic distances among thousands of genes within bacterial pangenomes, facilitating the identification of co-localized gene clusters. The result of the method is a plot that integrates the information from *aurora*,

the MAGs-based test (if supplied), multiple annotation tools, and co-localization. Although annotation of prokaryotic genomes is still an open research area, many genes identified in microbial GWAS studies are poorly annotated (9). To address this, we integrated a broad set of available annotation tools to identify the function of the genes in the output. The user can additionally supply their own annotation file. The annotation results and basic statistics from *aurora* and the MAGs test are then displayed in the final interactive plot.

We used this analysis pipeline to identify host-associated genes and gene clusters in all the species of *Lactobacillaceae* with strong evidence of host adaptation: *Limosilactobacillus reuteri, Ligilactobacillus ruminis, Ligilactobacillus salivarius, Lactobacillus crispatus* and *Limosilactobacillus mucosae*. All co-localization plots are available here (link to an online tool will be inserted upon acceptance). The adaptation factors of *L. reuteri* to rodent host are well known (10–12). We used this dataset to demonstrate that the method reliably verifies all previously known adaptation factors and is also capable of identifying novel putative adaptation factors (see below). We however acknowledge that our method is dependent on manual examination of the final plot and is thus to some degree subjective. We therefore made the final plots of all these species available here (github link to an online tool will be inserted upon acceptance) and we deposited all databases, associated annotation files and *aurora* results separately (DOI 10.5281/zenodo.17582651).

Supplementary Discussion

In this section, we examined in detail the five species showing the strongest host adaptation, as identified in the main text based on the residualized SES measure (Figure 3C). For this analysis, we first constructed both a pangenome and a core-genome phylogenetic tree, which were used as inputs for *aurora*. For each gene in the pangenome, a P value was calculated using the MAGs test (see Supplementary Methods). The resulting pangenome was then

visualized using the newly developed visualization approach described above. In the final visualization, the 100 genes with the highest standardized residuals (representing the strength of association estimated by *aurora*) are highlighted in host-specific colors, whereas genes with $p < 0.05$ in the MAGs test are marked with a diamond symbol (Figures S6–S10). This figure served to identify the host-associated gene clusters discussed below. For each gene mentioned, we also report its rank in the *aurora* results, where ranks are based on the host-specific standardized residuals and reflect the relative effect size of each gene (e.g. gene with rank 1 has the highest effect size).

Limosilactobacillus reuteri habitat adaptation

Limosilactobacillus reuteri is a frequent inhabitant of mammalian gastrointestinal systems. *L. reuteri* comprises only a small part of the bacterial community in the human gut, but in the rodent forestomach, chicken crop and pig pars oesophagea *L. reuteri* is found in high abundance (Tannock, 1992, Walter, 2005). In these habitats, *L. reuteri* adheres to non-secretory epithelium and forms resistant biofilms (11). However, in the human small and large intestines, there is no analogous epithelium, and microorganisms adhere to and persist in the mucosa (13). Consequently, it has been proposed that the gastrointestinal tracts of rodents and poultry require a markedly different set of adaptation factors compared to those of humans (14). This distinction is evident in our genome analyses: 36% of the top 100 rodent-associated genes passed the MAGs test (*P* value < 0.05, see section above, Figure S6). Similarly, 25% of the poultry adaptation factors passed the MAGs test. In contrast, only 18%, 6%, 7%, and 7% of the putative adaptation factors in human, bovine, porcine, and non-human primate isolates, respectively, passed the test (Figure S6).

The absence of acquired adaptation mechanisms in these hosts has been previously discussed and attributed to the relatively recent colonization of these hosts by *L. reuteri*, in contrast to

the rodent and poultry lineages that have been established for millions of years (7,10,15,16). Moreover, it has been shown that only rodent and poultry isolates possess a colonizing advantage, being able to outcompete isolates from other hosts in their native environments (10,15). Although human and porcine isolates do not appear to stably colonize their hosts (15), *aurora* was still able to distinguish them from isolates originating from other hosts (Figure 4). However, *aurora* identified that a large proportion (25%) of human isolates are allochthonous. These strains are closely related to those from poultry and rodents (Figure 4). In contrast, cereal and dairy isolates lack any adaptation factors to those habitats (see supplementary data). This is in agreement with previous studies that show that sourdough *L. reuteri* isolates likely originate from rodents and are not restricted to sourdough (17,18). Based on our current findings, *L. reuteri* appears to be specialized to animal-associated habitats, including humans, with the strongest adaptation observed in rodent and poultry hosts.

Many of the key adaptation factors of *L. reuteri* in rodents are already well characterized (11), acting as a good benchmark to evaluate our findings. For each adaptation factor discussed, we report its rank, which was derived by sorting the *aurora* results according to standardized residuals. This rank reflects the effect size of the adaptation factor and facilitates its identification in the Supplementary Data, where representative sequences can be retrieved. Lastly, we made interactive co-localization plots for each analysed species available (link to an online tool will be inserted upon acceptance).

Using co-localization mapping method, we found that the genes encoding the identified rodent adaptation factors are co-localized (Figure S6). One of the largest clusters of adaptation-associated genes that passed the MAG-based validation was the urease operon (*ureABCDEFG*, ranks 2 -12), which has previously been identified as an essential factor for rodent colonization (7). It was suggested that the function of the operon is to allow survival in

acidic conditions in rodent forestomach, allow biofilm formation by ameliorating the buildup of acidic metabolic end products, and by providing a nitrogen source for growth (12,16,19). Adjacent to this operon is a gene *ngrA* (rank 13). This gene encodes an ammonium transporter which is functionally consistent with its genomic context, as it facilitates the export of ammonia produced by urease activity. Another transporter within the cluster is the LarMNOQ system (rank 4), which encodes a cobalt/nickel transporter. This is functionally relevant, as the urease enzyme active site specifically requires two nickel ions to catalyze the hydrolysis of urea into ammonia. The co-localization of the urease operon with a nickel transporter has also been observed in other organisms, including *Geobacillus* and *Microbulbifer*, suggesting a conserved genomic arrangement that facilitates efficient urease function (20). The urease operon and cobalt/nickel transporter genes exhibit similar presence–absence patterns across *L. reuteri* strains. In addition to their prevalence in rodent isolates, these genes were also found in some bovine and primate isolates, occur rarely in human and porcine isolates, and are completely absent in poultry-associated strains (see Supplementary Data).

The gene with the highest standardized residual value was *o*-succinylbenzoate synthase (*menC*, rank 1), which was present in 94% of rodent-associated isolates but nearly absent from isolates derived from other habitats. *menC* encodes an enzyme involved in the biosynthesis of menaquinone (vitamin K_2). Notably, supplementation with menaquinone and heme has previously been shown to enhance growth efficiency and stress resistance in lactic acid bacteria, suggesting a potential adaptive advantage of *menC* in the rodent gut environment (21,22). In addition, a putative ferrous iron transporter (rank 124) is co-localized with *menC* and another such transporter with rank 32 is localized in a separate gene cluster. The transporters may facilitate the uptake of Fe^{2+} ions, which are essential for heme biosynthesis. Additionally, homologues of the *cydABCD* operon, a heme-dependent

cytochrome capable of generating a proton motive force, were found in *L. reuteri* (21). These findings raise the possibility that rodent adapted *L. reuteri* may have acquired *menC* to enable menaquinone biosynthesis for respiration in the presence of heme. This mechanism was previously described in other lactic acid bacteria. For example, *Lactococcus cremoris* produces menaquinone and, when exposed to both oxygen and heme, switches to menaquinone-dependent aerobic respiration (23). This would be consistent with the fact that the rodent GIT is not strictly anaerobic, potentially allowing for respiratory metabolism under certain conditions (24). However, *menC* is not the terminal enzyme in the menaquinone biosynthesis pathway. The pangenome constructed herein shows that the *L. reuteri* pangenome includes downstream biosynthetic genes such as *menE*, *menB*, *menA*, and *menG*, as well as upstream genes *menH* and *menD*. All of these genes are part of the core genome and are thus not specific to rodent isolates.

To our knowledge, there are no published studies demonstrating that *L. reuteri* synthesizes menaquinone or engages in respiration under physiologically relevant conditions. Although the full biosynthetic pathway may not be functional, the organism would likely need to import 6-hydroxycyclohexa-2,4-diene-1-carboxylate or another upstream intermediate if *menC* were functionally active in respiration. Alternatively, the presence of the *menC* gene in rodent isolates may be due to linkage disequilibrium with neighboring genes which are the object of selection in this host. Notably, *menC* is located adjacent to *arsC* (rank 44), which encodes an arsenate reductase known to confer arsenic resistance in *Lactobacillaceae* (25). We previously identified *arsC* as a poultry adaptation factor in *Salmonella enterica* subsp. Typhimurium (1). Both *menC* and *arsC* are flanked by transcriptional regulators, two of the LytTR family (ranks 16 and 63) and one BcrR-type regulator (rank 30), as well as phage-associated genes, including a holin and a phage head-tail joining protein. This genomic context suggests that *menC* and/or *arsC* may be embedded within a prophage-derived mobile

genetic element. Prophages are common in *L. reuteri* and have frequently been associated with fitness advantages (26).

Among the genes with high standardized residual values, two co-localized genes were involved in glutamate metabolism: glutaminase and the glutamate/GABA antiporter (rank 67 and 53 respectively). Previous studies have demonstrated that supplementing acidic media with glutamate significantly enhanced the survival of the rodent isolate *L. reuteri* 100-23, improving viability by 100-fold (27). These two genes function analogously to the urease operon (i.e. in affording protection against low pH) by converting host-derived glutamine into ammonia and γ-aminobutyric acid (GABA). The ammonia produced can buffer extracellular pH or serve as a nitrogen source, whereas glutamate derived from glutamine is decarboxylated to GABA and exported via the glutamate/GABA antiporter.

We identified the SecA2–SecY2 protein translocation system (ranks 14 – 108) as a potential rodent-specific adaptation factor. The functional importance of this system has been previously demonstrated: deletion of SecA2 in the rodent isolate *Lactobacillus reuteri* 100-23 abrogated its ability to adhere to the mouse forestomach epithelium and form biofilms (6,16). Proteomic analyses further revealed that the SecA2 mutant fails to secrete the key surface protein Lr_70902 – a serine-rich repeat protein annotated in our pangenome analysis as a mucin-binding protein (group_1363) (16). Contrary to earlier findings suggesting that the SecA2–SecY2 system is restricted to rodent and porcine isolates and absent in human and poultry strains (6,7,16,28), our pangenome analysis shows a broader distribution: the system is present in 19% of human isolates, 10% of poultry isolates, and 50% of primate isolates, but remains entirely absent in bovine strains. In *L. reuteri*, the SecA2–SecY2 locus is co-localized with multiple glycosyltransferase-encoding genes (including *gtfE*, *glyD*, and *glyG*, ranks 110, 120, 149, respectively), suggesting a specialized glycosyltransferase system for its serine-rich adhesins. These glycan modifications stabilize the large adhesin as well as protect

the adhesin from proteolytic degradation, facilitate interactions with host lectins, or masks antigenic epitopes, contributing to immune evasion (6). Analogous to the glycosylation of streptococcal serine-rich adhesins, *L. reuteri* was previously predicted to glycosylate Lr_70902 in a stepwise fashion (6,29). This process produces complex O-linked oligosaccharides.

Glucosyltransferase activity plays a key role in autoaggregation under acidic conditions in *L. reuteri* TMW1.106 (30). In agreement with this we have also identified two glucosyltransferases *gtfA* and *gtfB* (rank 100). A *gtfA* mutant exhibited significantly reduced resistance to lactic acid compared to the wild-type strain (30), highlighting an additional role for glycosylation in acid stress tolerance. GtfA and GtfB synthesize extracellular polysaccharides that create a physical biofilm matrix. In addition to acid resistance, this glucan facilitates bacterial auto aggregation and eventually biofilm formation in *L. reuteri* (31). Additionally, the extracellular matrix help the bacteria to stick to surfaces in the gut environment (30).

Another set of putative adaptation factors were that was identified with *aurora*: glycerol-3-phosphate transporter (GlpT, rank 56), extracellular-matrix-binding protein (EbhA, rank 66), pyrroline-5-carboxylate reductase (ProC, 123), PadR family transcription regulator (rank 89), and xylose and isoprimeverose utilization (XylA, XylP, XylT, AraC regulator, ranks 81.5 – 95). These genes do not seem to be functionally related to each other, but all are co-localized. GlpT can import glycerol-3-phosphate (G3P) from the lumen into the cell. In *L. reuteri*, G3P uptake can feed into energy metabolism or membrane repair, or it can be converted to glycerol. Importantly, *L. reuteri* ferments glycerol to 3-hydroxypropionaldehyde (3-HPA, “reuterin”) via a cyanocobalamin (B12)-dependent glycerol dehydratase. Reuterin inhibits the growth of multiple gastrointestinal pathogens (32), thus benefiting *L. reuteri* as well as the rodent host. The production of reuterin is not often observed in rodent isolates (33) due to the

absence of the corresponding metabolic island for propanediol utilization. In many Gram-positive bacteria, EbhA/Embp family proteins are large surface proteins that bind host matrix components and mediate adhesion (34). In *L. reuteri*, a collagen-binding protein (Cnb) has been identified, suggesting that this species uses surface proteins to adhere to host tissues (8). An EbhA-like protein in rodent strains could similarly tether bacteria to the forestomach or intestinal mucosa of mice/rats. ProC catalyzes the final step in proline biosynthesis. In bacteria, proline accumulation is a well-known response to various stresses. Proline acts as a compatible solute and stabilizes proteins, membranes, and balances osmotic pressure (35).

The combined function of XylA, XylP, XylT, and AraC is to import and catabolize plant pentoses. In the mouse intestine, *L. reuteri* was found to induce the xylose isomerase gene (*xylA*) in vivo (36). Later studies using comparative genomic analysis found that the xylose cluster was associated with rodent lineages (7). Additionally, plant-associated *Lactiplantibacillus pentosus* strain was also shown to harbour the isoprimeverose transporter *xylP* (37). Isoprimeverose is a xyloglucan oligosaccharide and could thus be part of the rodent diet.

Our analysis identified multiple mucin-binding genes with high standardized residuals, including group_3368 (rank 35), group_1363 (rank 50), and group_1208 (rank 87), along with several genes annotated as ABC transporters:group_2134 (rank 64), *ecfA1_3* (rank 80), and group_581 (ranks 122 and 138), which have previously been implicated in adhesion processes (38). Whereas these genes were enriched in rodent isolates, they were also found in other isolates. For example, the top-ranked adhesin, group_3368, was also present in the genomes of primate and human isolates.

The role of adhesion proteins in *Lactobacillus reuteri* host colonization is well established. Roos and Jonsson (2002) and later MacKenzie et al. (2010) identified and characterized the

mucus adhesion protein Mub in the pig isolate *L. reuteri* 1063 (39,40). Jensen et al. (2014) discovered an essential LPXTG-motif-containing adhesin in a *L. reuteri* strain isolated from human milk (41). Walter et al. (2005) identified a high-molecular-mass surface protein, Lsp, in the rodent isolate *L. reuteri* 100-23, as a host adaptation factor (42). This protein was later annotated as a serine-rich repeat protein (SRRP; group_1363), with its secretion shown to depend on the SecA2-SecY2 system (16,43). More recently, SRRPs have been implicated in host-specific colonization beyond *L. reuteri*. A serine-rich repeat protein was shown to be essential for *Lactiplantibacillus plantarum* colonization in *Drosophila melanogaster* (44). Similarly, the importance of SRRPs for host interaction has been recognized across various species from the family *Lactobacillaceae* (5). Furthermore, SRRP clusters, along with associated glycosyltransferase genes, have been found to be highly prevalent in Firmicutes. These gene clusters may hold the key to understanding host range specificity in Gram-positive bacteria. However, their identification and annotation remain challenging due to their considerable sequence divergence (5,45).

Compared to rodent-associated adaptation, the overall landscape of adaptation factors in other hosts remains less well defined. This is likely due to two main factors: the limited number of studies focusing on these hosts and the fact that rodents are considered the ancestral host of *L. reuteri* (10,16). Moreover, many of the top-ranked adaptation factors with high standardized residuals are genes associated with mobile genetic elements (recombinases, resolvases, toxin–antitoxin systems, holins, and transposases). Phage-related genes – such as those encoding phage head closure proteins, head–tail joining proteins, capsid proteins, tail domain-containing proteins, and phage transcriptional regulators – also frequently appear among the top candidates. Whereas these genes are host-associated, they are unlikely to confer direct fitness advantages. However, if these mobile elements serve as vectors for horizontally

transferring true adaptation factors, then this could be inferred through our co-localization analysis.

We identified three genes encoding 2-dehydropantoate 2-reductase (ranks: 30.5, 85, and 103) as putative poultry-associated adaptation factors. This enzyme plays a role in the biosynthesis and salvage of pantothenate, a precursor of coenzyme A (CoA). Notably, *L. reuteri* is auxotrophic for pantothenate and several other vitamins (28). One of these genes is co-localized with that encoding a hypothetical protein and genes associated with mobile genetic elements, whereas the other two are located near genes encoding a GNAT family N-acetyltransferase (rank 59) and pyruvate carboxylase (rank 38.5). GNAT family N-acetyltransferases catalyze the transfer of acetyl groups to a broad range of substrates, influencing gene expression and enhancing stress resistance (46). These enzymes utilize acetyl-CoA, synthesized from pantothenate, as a substrate. Acetyl-CoA also serves as an allosteric activator of pyruvate carboxylase, a key enzyme in gluconeogenesis and anaplerotic metabolic pathways. Although the presence of multiple pantothenate and CoA producing 2-dehydropantoate 2-reductases in our results is notable, the wide-ranging metabolic roles of pantothenate and CoA complicate efforts to identify a specific adaptive benefit conferred by these genes.

Beyond pantothenate metabolism, poultry isolates of *L. reuteri* also harbor genes involved in the biosynthesis of other essential cofactors, including folate and cobalamin (vitamin B_{12}). Additionally, two highly ranked cobalamin-related genes: threonine phosphate decarboxylase (*cobD*, rank 22) and adenosylcobalamin/α-ribazole phosphatase (rank 14.5) were identified. The ability of *L. reuteri* to synthesize cobalamin has been previously confirmed (47). In addition to these genes, we also detected the presence of a full cobalamin biosynthesis gene cluster and other genes associated with the well known *pdu-cbi-cob-hem* cluster. However these genes had lower ranks (233–258.5). This cluster is responsible for production of

antimicrobial compound reuterin (32) and is prevalent in poultry isolates (76%) and also found in human (73%), bovine (44%), and porcine (43%) isolates. In contrast, the cluster was nearly absent in rodent-associated strains (10%). In addition, two genes involved in early steps of cobalamin biosynthesis: uroporphyrinogen III synthase and uroporphyrinogen III methyltransferase (ranks 14.5 and 22, respectively) were found in approximately two-thirds of poultry isolates but were largely absent in strains from other hosts. Cobalamin may serve multiple functions within *L. reuteri*, one of the most notable being its role in the biosynthesis of the antimicrobial compound reuterin. Similar to rodent-adapted strains, poultry-adapted *L. reuteri* may utilize the cobalamin-dependent enzyme glycerol dehydratase to convert glycerol into reuterin, thereby enhancing competitive fitness in the gut environment (32).

Several genes associated with cobalamin metabolism in *L. reuteri* poultry isolates are also involved in nucleotide biosynthesis. Among these are adenosylcobalamin-dependent ribonucleoside-triphosphate reductase (rank 6) and carbamoyl phosphate synthase (rank 77). Carbamoyl phosphate synthase catalyzes the production of carbamoyl phosphate, a precursor in both pyrimidine and arginine biosynthesis. Ribonucleoside-triphosphate reductase is essential for the conversion of ribonucleotides into deoxyribonucleotides, enabling the bacterium to synthesize DNA precursors *de novo*, rather than relying solely on dietary sources (28,47). In addition to nucleotide biosynthesis, poultry-associated *L. reuteri* strains may catabolize host-derived or dietary nucleosides via deoxyribose-phosphate aldolase (rank 17.5) and a co-localized deoxyribonucleoside regulator (rank 17.5). Deoxyribose-phosphate aldolase cleaves 2-deoxyribose-5-phosphate, a breakdown product of DNA or nucleosides, into acetaldehyde and glyceraldehyde-3-phosphate, with the latter feeding directly into glycolysis (48). This pathway may allow the organism to extract both carbon and energy from nucleic acid turnover products in the gut environment.

We also identified the gene encoding a predicted large-conductance mechanosensitive channel (rank 83) that was enriched in poultry isolates. This protein protects the cell against sudden osmotic shifts, which is a common stressor in the gastrointestinal tract. In response to increased membrane tension, the channel opens to release small osmolytes and prevent cell lysis (49).

The poultry-associated gene annotated as a galactoside/maltose O-acetyltransferase in *L. reuteri* (rank 72) acetylates the C-6 hydroxyl groups of N-acetylmuramic acid in peptidoglycan. Peptidoglycan O-acetylation is a well-known strategy for Gram-positive bacteria to resist lytic enzymes of the innate immune system (50). O-acetylation blocks lysozyme from cleaving the glycan backbone of peptidoglycan In the poultry gastrointestinal tract, lysozyme is abundant (birds secrete lysozyme in intestinal mucus and also in the egg white (51)). Indeed, the enzyme catalyzing peptidoglycan O-acetylation is considered a virulence or persistence factor in many bacteria and has also been found in *L. reuteri* (50).

The formyl/oxalate CoA-transferase gene (*frc*, rank 40) enables *L. reuteri* to engage in a rare form of anaerobic metabolism: the degradation of oxalate (52,53). Oxalate is abundant in many plants and is a common component of herbivorous diets, including certain poultry feeds. When present in the gut, *frc* catalyzes the transfer of CoA from formyl-CoA to oxalate, producing oxalyl-CoA and formate. In oxalate-degrading bacteria such as *Oxalobacter formigenes*, oxalyl-CoA is further decarboxylated to form formyl-CoA and CO_2 via *oxc* (oxalyl-CoA decarboxylase), effectively closing a metabolic loop that allows for oxalate detoxification and formate production (52). *L. reuteri* often harbors both *frc* and *oxc* , suggesting a potential capacity for oxalate metabolism (53). In the current pangenome analysis, *oxc* is part of the core genome. Notably, the rodent isolate *L. reuteri* 100-23C also contains both the *frc* and *oxc* genes (53). In contrast, two human-derived strains carry an *frc* gene fragment. Functional studies have shown that a *frc*-deficient mutant was outcompeted

by the wild-type strain in the mouse intestine and exhibited reduced growth under acidic conditions (53). However, neither the wild-type nor the mutant demonstrated measurable oxalate catabolism under experimental conditions. These findings suggest that whereas *frc* and *oxc* may have originated as components of an oxalate-degrading pathway, their primary role in *L. reuteri* may instead relate to pH regulation or broader metabolic adaptation rather than true oxalotrophy.

Surprisingly the SecA2–SecY2 protein translocation system (rank 52.5) and associated glycosyltransferases (gtfA: rank 52.5, gtfB: rank 52.5) were also found to be associated with poultry. This set of genes is a distinct gene family from the SecA2–SecY2 translocation system found in rodents. The average nucleotide identity between rodent and poultry SecA2 was 71.4% and SecY2 73.7%. Just like the rodent SecA2–SecY2 system, the poultry equivalent was also found to be co-localized with glycosyltransferases; however, unlike the rodent SecA2–SecY2 system the poultry system is not co-localized with any surface adhesin. It is thus not clear which protein is the main target of these genes. *Clostridioides difficile* was found to be able to use the SecA2–SecY2 to export and glycosylate S-layer proteins (54). However, in the top results we have not identified any S-layer protein nor any other surface adhesin. A small protein with high rank (52.5) group_4555 is co-localized with this cluster but its function is not known, and it is unlikely that it is surface adhesin or S-layer protein due to its small coding sequence size (189 bp). An α-ketoglutarate permease (rank 185) is also co-localized with the cluster, but this protein is likely not related to SecA2–SecY2 system. Additionally, a glucosyltransferase *gtf3* (rank 111) is also co-localized with the cluster. This enzyme is known to be transported out of the cell and contributes to biofilm formation and cell aggregation (31). The export of this enzyme in *Streptococcus mutans* is not dependent on SecA2–SecY2 system but rather relies on the general Sec pathway (55). Thus, it not clear what protein the SecA2–SecY2 system in poultry isolates acts on.

Poultry isolates of *L. reuteri* encode key folate-pathway (vitamin B9) enzymes dihydroneopterin aldolase (FolB, rank 89.5), hydroxymethyldihydropteridine diphosphokinase (FolK, rank 3), folylpolyglutamate synthase (FolC, rank 13) and dihydrofolate reductase (FolA, rank 99). These enzymes all participate in the biosynthesis and recycling of folate derivatives, which are critical to carry out nucleotide and amino acid biosynthesis. Only one gene in this pathway, whose ultimate product is tetrahydrofolate (THF), is missing (folP, dihydropteroate synthase). However, this gene may be in the top results, but it could be misannotated. Many lactobacilli share the folate synthesis capability (56) as THF has many essential roles. In particular, 10-formyl-THF (synthesized from THF) is required for purine, thymidine and methionine biosynthesis. The cofactor 10-formyl THF is also required for the initiation of protein synthesis. Hence, robust folate biosynthesis ensures that *L. reuteri* can maintain nucleotide levels and proteosynthesis under nutrient limitation. In addition two genes, nicotinamidase and nicotinate phosphoribosyltransferase, are co-locallized with some of the folate synthesis genes. These two genes encode sequentially-acting enzymes in a NAD^+ salvage route found in many bacteria, fungi, and invertebrates (57). This pathway allows organisms to recycle nicotinamide, a byproduct of NAD^+-consuming reactions, back into NAD^+. To the best of our knowledge, direct relationship of NAD^+ to folate synthesis is not known but folate-mediated metabolic pathway involve the transfer of single-carbon units essential for nucleotide and amino acid biosynthesis. Enzymes like methylenetetrahydrofolate dehydrogenase require $NADP^+$ as a cofactor, converting it to NADPH during the oxidation of 5,10-methylene-THF. Thus, maintaining adequate $NADP^+$/NADPH levels, which are both derived from NAD^+, is crucial for these folate-dependent processes. Inspection of the molecular structure of the folate molecule shows that folate is a tripartite molecule, composed of a pterin residue, para-aminobenzoic acid and a γ-linked glutamate tail. In addition the 5-oxoprolinase complex (*pxpA*/*pxpB*/*pxpC*, rank 116.5)

converts 5-oxoproline into this glutamate. 5-oxoproline arises spontaneously from protein turnover and from glutathione metabolism, so its disposal is important. In *Bacillus subtilis*, loss of *pxpABC* slows growth and causes 5-oxoproline accumulation (58). Thus in *L. reuteri* this system likely salvages nitrogen and carbon from 5-oxoproline, feeding glutamate back into amino-acid metabolism and folate synthesis.

Co-localized with *pxpABC* is 2-keto-4-pentenoate hydratase (MhpD , rank 16), divalent metal canion transporter (MntH, rank 116.5), and biotin-dependent carboxylases (AccB/AccC/PycA, rank 116.5). The enzyme MhpD participates in phenylalanine and related aromatic compound degradation (59). In *L. reuteri* this could enable the bacterium to exploit plant- or host-derived phenolic substrates as alternative carbon sources. The acetyl-CoA carboxylase (AccC plus the biotin-carrier AccB) and pyruvate carboxylase (PycA) drive fatty-acid synthesis (via malonyl-CoA) and synthesis of the tricarboxylic acid cycle intermediate oxaloacetate (60). Fatty acid metabolism as well as glutamate synthesis were found to be upregulated under acid induced stress in *L. paracasei* (61). This potentially links the *accC/accB/pycA* cluster to *pxpABC;* both could thus be an adaptation to acidic conditions in poultry gut.

As discussed above, the adaptation of porcine *L. reuteri* isolates to their host is not well defined. This is likely because *L. reuteri* colonized pigs only recently (10). Nonetheless, *aurora* can still clearly distinguish porcine isolates from those derived from other hosts. To achieve this, *aurora* primarily identifies genes enriched in porcine isolates; however, many of these genes are unlikely to confer any evolutionary advantage. Of the top 100 genes identified, 95 were either mobile genetic elements (primarily phages and insertion sequence elements) or proteins without known annotation. The remaining five annotated genes were: Glycerol-3-phosphate transporter (PgtP, rank 13.5), rRNA adenine N-6-methyltransferase (*erm*, rank 44), tetracycline resistance ribosomal protection protein (*tetW*, rank 45),

arabinogalactan endo-β-1,4-galactanase (rank 65), and pseudouridine synthase (RluD, rank 85).

The functional roles of these genes in porcine adaptation remain speculative due to the lack of porcine-specific studies. However, *pgtP* has been found to be highly expressed in *L. plantarum* in both mice and humans (62), suggesting it may broaden the organism's substrate range. Glycerol, notably, is a precursor for reuterin synthesis, and the gene cluster responsible for this pathway is present in 43% of porcine isolates. Both *tetW* and *erm* have been previously implicated in tetracycline and erythromycin resistance in *L. reuteri*, respectively (63). Tetracycline resistance is particularly prevalent among porcine isolates, with 82% harboring the *tetW* gene (64). This aligns with antibiotic usage patterns in pig farming, where tetracyclines and penicillins are among the most frequently administered antibiotics (65).

The arabinogalactan endo-β-1,4-galactanase enzyme specifically cleaves β-1,4-galactosidic linkages in arabinogalactan, a polysaccharide found in plant cell wall pectins (66). Typical porcine diets are rich in cereals, legumes, and other plant-derived materials, so this enzyme may enable *L. reuteri* to break down complex carbohydrates and utilize them for fermentation. Pseudouridine synthases catalyze the isomerization of uridine to pseudouridine in RNA, enhancing RNA stability. In bacteria, these enzymes are known to improve survival under stress conditions (67). Additionally, pseudouridine modifications may reduce RNA immunogenicity, potentially aiding immune evasion (68).

It is worth noting that the genes most strongly associated with porcine isolates show high specificity. For instance, the top gene, group_5806 (IS3 family transposase), is found in 63% of porcine isolates but is nearly absent in isolates from other hosts. It has been proposed that host colonization begins with transient interactions, during which bacterial genomes undergo rapid decay whereas acquiring new adaptive elements (69–71). Our findings suggest that

porcine isolates may be in this early phase of colonization, where, apart from a few genes that enhance fitness, only porcine-specific mobile elements have been integrated into the genome. Moreover, our method focuses exclusively on gene presence or absence. It is therefore possible indeed likely that key adaptive changes have already occurred at the sequence level within individual genes. A complementary approach based on k-mers or unitigs would be well-suited to capture such variation (72).

We identified more putative adaptation factors in *L. reuteri* isolates from non-human primates than from pigs. One notable finding was a complete gene cluster for the utilization of uronic acids (e.g., galacturonate and glucuronate), which ranked between 116-125. This metabolic pathway was first described in *Paucilactobacillus suebicus* (73). Uronic acids are commonly found in pectin, a component of plant cell walls that is especially abundant in citrus fruits which is a dietary staple for many primates. Although the pathway was found in only 27% of primate isolates, it was nearly absent in all other host groups except poultry (24%). However, none of the genes in this cluster passed the MAGs test. This is likely due to low statistical power, as only eight MAGs were available from primates (Figure S6). The test may not be sensitive enough to detect adaptation factors with small effect sizes in this context.

We also discovered a cluster of four genes that may enable primate *L. reuteri* isolates to detoxify tannins: polyphenolic compounds present in fruits and leaves that inhibit microbial growth. It has been shown that *L. plantarum* can convert tannins to pyrogallol, a less toxic compound (74). The identified gene cluster includes a hypothetical protein with an LpdD-like domain (group_4483, rank 75), flavin prenyltransferase (UbiX, rank 75), gallate decarboxylase (UbiD, rank 75), and a transcriptional regulator (LysR, rank 75). These genes likely cooperate to catabolize gallic acid, a tannin degradation product, into pyrogallol (75). This cluster appears unique to primate isolates and is found in approximately 20% of them. Additionally, we identified a three-gene cluster comprising of fructokinase, a SWEET family

sugar transporter, and a transcriptional regulator (ranks 44.5–57). This cluster may facilitate efficient fructose metabolism. Fructose is a common sugar in fruits and nectar, both of which are abundant in primate diets.

Importantly, most candidate colonization factors in primate isolates are co-localized within a single large gene cluster (ranks 1–75). This cluster includes genes involved in respiration-like metabolism, oxidative stress response, carbohydrate utilization, and nutrient storage. Key enzymes encoded include fumarate reductase flavoprotein subunit, FMN-dependent NADH-quinone oxidoreductase, and several flavodoxins. Together, these genes may establish a partial electron transport chain, increasing energy yield and enhancing fitness in the gut environment. Fumarate reductase, for instance, has been shown to be essential for intestinal colonization by *E. coli* (76), and the associated oxidoreductases are especially beneficial under iron-limiting conditions (77). This cluster also includes the hydrogen peroxide-inducible activator (oxyR_1) and associated enzymes such as FMN reductase and NADH-flavin oxidoreductase. These components likely mitigate oxidative damage by maintaining pools of reduced flavins or quinones, which help neutralize reactive oxygen species (78).

Further genes in this cluster support nutrient acquisition, including endo-1,4-β-xylanase and a glucose uptake protein. Other enzymes: glucose-1-dehydrogenase, gluconic acid reductase, and phosphogluconolactonase, are involved in an alternative glucose metabolism pathway that generates additional NADPH, improving stress resistance (79). Another NADPH-linked enzyme, glyoxal reductase, detoxifies harmful compounds such as glyoxal and methylglyoxal by converting them into glycolate or similar products (80). The cluster also encodes poly(3-hydroxybutyrate) (PHB, prokaryotic carbon and energy reserve) depolymerase. Although direct accumulation of PHB by *Limosilactobacillus* has not been documented, the enzyme could enable utilization of PHB produced by other microbial community members (81).

Lastly, we identified a mucin-binding protein (rank 56) that was not co-localized with other genes but may play a key role in host colonization. This gene is present in 100% of primate strains and 89% of porcine strains, but absent in nearly all other hosts. It is therefore possible that pigs and primates share a common adhesion mechanism. Further k-mer-based GWAS would be needed to determine whether this protein in pigs and primates is structurally distinct.

We have shown previously that human isolates of *L. reuteri* are difficult to distinguish from poultry and rodent isolates using *aurora* (1). This overlap is evident herein as well, where several top-ranked human colonization factors are also among the top poultry colonization factors (see Supplementary Table), for example, the transcriptional activator BcrR (rank 2), MerR family transcriptional regulator (rank 5), and holin (rank 6). This observation may reflect the possibility that human isolates originally derived from poultry and/or rodents. This indeed has been suggested before (10). Additionally, previous studies have shown that human *L. reuteri* isolates are unable to outcompete strains from other hosts in human experimental colonizations (82). Moreover, these isolates are neither abundant nor prevalent in the human microbiota (83,84), further suggesting that human-associated *L. reuteri* may not be strictly host-adapted to humans.

Despite this, *aurora* was still able to distinguish human isolates from those of other hosts (Figure 4). We identified a gene cluster responsible for the production of exopolysaccharide (EPS) or capsular polysaccharide as a putative human adaptation factor. The cluster includes the following genes: glycosyltransferase family 4 protein (twice, ranks 34.5), O-unit flippase-like protein (rank 34.5), capsular polysaccharide synthesis protein (rank 34.5), glycosyltransferase family 2 protein (rank 34.5), a hypothetical protein (rank 34.5), and an acyltransferase (rank 84.5). EPS clusters are common in human gut-associated bacteria and serve multiple roles, including transit through the gastrointestinal tract, immune evasion or

modulation, mucus adhesion, and biofilm formation (85). This finding resonates with our results from *Ligilactobacillus salivarius* where we show that EPS production is a highly ranked human colonization factor (see below). The EPS cluster is unique to human isolates but, unlike in *L. salivarius,* the cluster in *L. reuteri* is low effect size and the EPS locus is found in only about 12% of strains.

We also identified two LPXTG cell wall-anchored proteins (group_3460 and group_6926, ranks 84.5). These proteins are found in just one additional poultry isolate, and otherwise are unique to human-derived strains, being present in 13% of human isolates. These genes likely encode surface adhesins similar to known *L. reuteri* factors such as Mub and SRRP (5,8,39), and likely play roles in mucus and epithelial adhesion, as well as interactions with the host immune system.

Another notable gene is that for an ATP-dependent Clp protease (rank 72), which is co-localized with several phage-related elements and unannotated high-ranking proteins. Clp proteases have been shown to improve survival under conditions of bile stress and heat shock in *L. reuteri*, suggesting an important role in surviving gastrointestinal transit (86,87). These proteins contribute to protein quality control, including folding, degradation of damaged or regulatory proteins, and stress response regulation.

Another gene cluster that we identified constitutes of four genes that all passed the MAG test. This cluster includes the histidine decarboxylase maturation protein HdcB (rank 62), histidine decarboxylase (HdcA, rank 62), arginine–ornithine antiporter (rank 62), and histidine-tRNA ligase (rank 67.5). HdcA/HdcB is responsible for histamine production and, in addition to *L. reuteri*, it has previously been described in *L. parabuchneri* and *L. buchneri* (88,89). Microbe-derived histamine has been shown to suppress host immune responses by inhibiting the TNF pathway (88). Wüthrich et al. (2017) conducted a detailed analysis of this cluster and

found it to be highly mobile and frequently associated with species found in the human gut and in food production environments (89). In addition to its immunomodulatory role, this gene cluster has also been implicated in acid resistance (89). The cluster is present in 47% of human isolates and is nearly absent in strains from other hosts.

Another cluster likely involved in the synthesis of cell wall glycopolymers includes genes for teichoic acid glycerol-phosphate transferase (rank 134) and teichuronic acid biosynthesis proteins TuaB and TuaG (rank 134). Teichoic and teichuronic acids are known adhesion factors important for binding to intestinal epithelial cells (90). Whereas all lactobacilli possess teichoic acids, their structure, abundance, and functional properties can vary significantly (91). This cluster also includes three glucosyltransferases: lipopolysaccharide 1,6-galactosyltransferase, inositol-3-phosphate glycosyltransferase, and glycosyltransferase GlyG (all rank 134), as well as erythritol 4-phosphate cytidylyltransferase (rank 134). These enzymes may contribute to further cell wall modifications, though their precise functions and biological relevance remain to be clarified.

The final host from which *L. reuteri* was isolated in our dataset is bovine. Whereas bovine isolates are distinguishable from the rest of the isolates using the *aurora* method, we could not find any annotated high effect size genes outside of those belonging to mobile genetic elements.

Ligilactobacillus ruminis habitat adaptation

Ligilactobacillus ruminis has been isolated from the human gastrointestinal tract as well as from cows, pigs, dogs, and horses (92–94) (Figure 1). In our analyses, however, we excluded dog and horse strains due to the limited number of available genome sequences. Strains from

the remaining three hosts clustered into host-specific lineages, consistent with previous findings (93). In additionIn addition, we observed two large distinct human-specific lineages and two bovine and porcine lineages with common ancestor (Figure 4).

Although several large-scale investigations into host colonization factors of *L. ruminis* have been published (93,95,96), to the best of our knowledge, no genome-wide association study (GWAS) has been conducted to date.. We focused initially on human colonization, because human isolates are the most abundant in our dataset and have been the subject of previous studies. Earlier reports demonstrated that human-adapted strains harbor an expanded repertoire of carbohydrate utilization genes. For example, lactose utilization has been described as unique to human isolates, whereas sucrose fermentation is absent in pig isolates (95). Our results are in agreement with these findings: a substantial proportion of human-specific adaptation factors were associated with carbohydrate metabolism. These included β-galactosidase and associated lactose/galactose/raffinose permease (rank 33), mannose PTS system (rank 38.5), multiple sugar transport protein (rank 38.5), ribose transport protein co-localized with a two-component system NarL (rank 44), aryl alcohol dehydrogenase (rank 33), glucoside PTS system (rank 38.5), cellobiose PTS transport system (ranks 22 and 23), lactose PTS system (rank 133) and the co-localized lactose catabolic operon (tagatose-6-phosphate pathway, ranks 124.5–128), aldouronate transport system substrate-binding protein (rank 12), xylobiose transport system substrate-binding protein (rank 33), D-xylose transport protein (rank 80), α-amylase (rank 71) with a co-localized mannose/sorbose PTS system (rank 124.5), and three co-localized genes: sucrose 6-phosphate hydrolase (rank 3.5), sucrose permease (rank 3.5), and a transcriptional regulator (rank 5).

These genes suggest that human-associated *L. ruminis* strains are equipped to catabolize multiple carbohydrates present in the human diet, including lactose, sucrose, mannose, xylose, and cellobiose. *L. ruminis* is the only species of the *Lactobacillaceae* whose

prevalence in human stool is above 10% and in some continents (Asia, Africa, South America) the prevalence reaches 100% (84). Presence of these genes thus underscores possible adaptation to diverse human dietary patterns. Notably, the sucrose utilization operon represented a high-effect-size adaptation factor, detected in 89% of all human isolates. In contrast, genes required for cellobiose utilization (cellobiose being a component of dietary fibers such as cellulose) were detected in only 49% of human strains. Similarly, α-amylase, an essential enzyme for fiber degradation, was found in only 47% of human-associated isolates. Together, these observations suggest that *L. ruminis* may be adapting to modern human diets characterized by a high availability of rapidly absorbed sugars. *L. ruminis* is unique among the *Lactobacillaceae* species in that it is absent in foods but prevalent in gut (84,97). This pattern further supports the notion of host specialization. Beyond carbohydrate utilization, we also identified the oligopeptide ABC transporter (rank 28) and a co-localized proline iminopeptidase (rank 14.5). The latter is a peptidase that cleaves N-terminal proline residues from peptides imported by the ABC transporter. Together, these enzymes may provide *L. ruminis* with additional sources of carbon and nitrogen and could also play a role in interspecies communication within the gut microbiota (98).

We also identified two genes associated with antibiotic resistance: penicillin amidase (shared with porcine isolates, rank 96) and linearmycin/streptolysin S transport permease (rank 14.5). In addition, we detected a gene annotated as a bacteriocin (rank 47.5), a class of antimicrobial peptides known to contribute to colonization. Notably, *L. ruminis* is known to produce primarily class II bacteriocins, which have been implicated in niche competition within the gastrointestinal tract (99). A staphylococcal competence inhibitor (rank 57) was also identified as human colonization factor. This gene encodes a secreted protein that, in *Staphylococcus aureus*, is involved in immune evasion and biofilm formation (100), but its role in *L. ruminis* is unknown.

Furthermore, we detected three co-localized genes: flagellar protein FliB (rank 139), an oxidoreductase (rank 75.5), and a peptidoglycan LD-endopeptidase (rank 75.5). The presence of FliB is particularly notable, as human *L. ruminis* isolates have been reported to be non-motile and do not express flagella (101). FliB is a methylase that modifies flagellin, the structural subunit of flagella, but its presence alone does not imply motility. It is possible that in non-motile strains, this gene may have evolved to fulfil a different function.

In bovine-adapted strains, we found limited evidence of sugar or sugar alcohol utilization genes. These findings are consistent with previous studies: when human and bovine *L. ruminis* isolates were tested on 50 different substrates, fructooligosaccharides were found to be human-specific, whereas no bovine-strain specific carbon sources were identified (102). However, we found one notable exception: a gene cluster containing glycerol uptake facilitator protein and dihydroxyacetone kinase (DhKLM) (ranks 39.5). These genes are predicted to enable *L. ruminis* to utilize glycerol, which is first transported into the cell via the facilitator protein and subsequently oxidized to dihydroxyacetone by glycerol dehydrogenase. The dihydroxyacetone is then phosphorylated to dihydroxyacetone phosphate by DhKLM, entering central metabolism (103).

Another gene potentially involved in substrate utilization is acetoin permease (rank 14.5). Whereas some pseudomonads can use acetoin as a sole carbon source (104), in *L. ruminis* this compound is more likely involved in acid resistance, as observed in other bacteria (105). We also identified two genes related to motility and pilus formation: a gliding motility-associated transport system auxiliary component (rank 45.5) and a pilus assembly protein (rank 14.5). This is consistent with previous work showing that bovine isolates of *L. ruminis* are motile, a trait also shared with porcine isolates (106). The fact that only two motility-related genes emerged as bovine-associated likely reflects sequence divergence within the broader motility operon. The bovine and porcine homologues of these genes may be

sufficiently distinct to form separate gene clusters, masking their shared function at the sequence similarity level.

The majority of bovine-associated genes in *L. ruminis* were related to microbial competition. We identified a gene cluster comprising three copies of EcoRI restriction endonuclease (ranks 96.5), a bacteriocin immunity protein (rank 96.5), and a SEFIR domain-containing protein (rank 46.5). SEFIR domains are structurally related to the TIR domain and may play a role in bacterial immunomodulation and host interactions (107).

In addition, we detected three smaller gene clusters associated with bacteriocin production and transport: linearmycin/streptolysin S permease (rank 14.5), co-localized with microcin B17 processing protein (rank 14.5); lactococcin-G-processing and transport ATP-binding protein LagD (rank 30), co-localized with a bacteriocin gene (rank 156.5); type III restriction-modification enzyme (rank 96.5) co-localized with a lanthipeptide synthase (rank 96.5). Another gene cluster contained three co-localized genes involved in bacteriocin-related functions: a bacteriocin immunity protein, pediocin bacteriocin, and bacteriocin transport protein (ranks 53), further highlighting the importance of antimicrobial mechanisms in bovine strains. Beyond bacteriocins and restriction-modification systems, we also identified seven co-localized genes encoding a CRISPR-Cas system, suggesting an additional defence mechanism against mobile genetic elements. The significance of these systems is discussed in the main text of this article.

Previous studies have shown that the bovine isolate *L. ruminis* ATCC 27782 dedicates a substantial portion of its genome – 62 genes, comprising approximately 3.4% of the total genome length – to exopolysaccharide (EPS) biosynthesis (108). EPS is a known contributor to host colonization and immune evasion. In agreement with this, we identified a large gene cluster encoding multiple enzymes involved in EPS synthesis, including α-1,6-

mannosyltransferase, α-GlcNAc α-1,2-L-rhamnosyltransferase, UDP-glucose dehydrogenase, polysaccharide transporter, and O-antigen polymerase (all rank 96.5). These EPS genes were co-localized with genes related to phosphocholine metabolism, including choline kinase, choline-phosphate cytidylyltransferase, and choline phosphotransferase (all rank 14.5). Together, these genes likely enable *L. ruminis* to modify its cell wall with phosphocholine moieties. This modification proceeds via the formation of cytidine 5′-diphosphocholine and subsequent transfer to teichoic acids. Phosphocholine decorations serve as anchoring sites for choline-binding proteins, which function as adhesins in several Gram-positive species (109).

We also identified co-localized genes involved in amino acid transport and biosynthesis. Transport systems included those for L-cysteine, glutamine, and a permease for arginine/lysine/histidine (ranks 39.5), whereas biosynthetic genes included 2-oxoglutarate reductase, acetylornithine deacetylase, arginine deiminase, and histidinol phosphate aminotransferase (all rank 49.5). Notably, the cysteine transport system has been shown to be critical for acid resistance, membrane integrity, and epithelial adhesion in *Listeria monocytogenes* (110,111). The arginine deiminase identified here is the first enzyme of the arginine deiminase pathway, a metabolic route that allows anaerobic arginine degradation whereas producing ATP, ammonia, and CO_2. This pathway is particularly relevant under acidic conditions, where ammonia helps buffer intracellular pH (112). The arginine deiminase pathway has also been reported in other lactobacilli (113). Another gene in this cluster, 4-aminobutyrate aminotransferase (rank 49.5), catalyzes the conversion of GABA (4-aminobutyrate) to L-glutamate. GABA metabolism serves as an additional buffering system by importing glutamate and exporting GABA whereas consuming intracellular protons thus raising cytosolic pH (114,115). This mechanism has been observed in *L. reuteri* adapted to the rodent gut (see above).

Porcine adaptation appears to be a more recent event compared to human adaptation (Figure 4). We identified only 16 genes that were in the top 100 genes identified by GWAS analysis and that passed the MAGs-based test (Figure S7). Of these, 10 genes exhibited a high effect size: defined as being present in at least 70% of porcine strains whereas absent in isolates from all other sources. However, 9 of these 10 genes are annotated as hypothetical proteins. The only gene with an assigned function was a DeoR family transcriptional regulator / glucitol operon repressor, which was present in all porcine strains. However, this regulator was not co-localized with any other colonization factors, and its specific regulatory targets remain unclear.

In addition to high-effect genes, we identified several lower-effect candidates with clearer putative roles in porcine colonization. For example, four co-localized genes encoding a Cu^{2+} transporter, two copper-binding proteins, and a fibronectin-binding protein (rank 77.5). Fibronectin-binding proteins are established adhesins, whereas the copper transport system may provide dual benefits. Copper is essential for bacterial enzymes as a redox cofactor but becomes toxic in excess concentrations (116). Thus, the ability to tightly regulate intracellular copper levels is likely advantageous. Moreover, copper supplementation is widely used in pig farming to enhance growth performance (117), creating a selective environment for copper-tolerant microbes. High dietary copper has been shown to suppress lactic acid bacteria in the pig gut, allowing copper-resistant *Enterobacteriaceae* to dominate (118). In this context, copper transport systems in *L. ruminis* could serve two roles: enabling survival under high concentrations of copper and facilitating copper scavenging in copper-limited environments. The gastrointestinal tract (at least in humans) is typically considered copper-poor (119). In addition to this gene cluster, a separate gene cluster encoding a copper transport repressor and a copper-exporting ATPase (ranks 77.5) was also identified.

Beyond copper, we also identified a high-affinity iron transporter (rank 77.5) and two co-localized genes: ferredoxin–$NADP^+$ reductase and alkyl hydroperoxide reductase C (both rank 77.5). The latter is a key enzyme in detoxifying hydrogen peroxide and organic hydroperoxides, thus protecting against oxidative stress (120). Ferredoxin–$NADP^+$ reductase regenerates NADPH, which powers antioxidant defence systems such as glutathione and thioredoxin, thereby sustaining peroxidase activity. The enzyme utilizes ferredoxin as an electron donor, which itself requires iron-sulfur clusters for function. The iron transporter may therefore contribute to ferredoxin biosynthesis by securing the necessary iron.

Finally, we detected a group of low-effect genes with diverse roles, including: β-1,6-galactofuranosyltransferase (rank 166.5), L-malate glycosyltransferase, α-1,6-mannosyltransferase, polysaccharide transporter, colanic acid/amylovoran biosynthesis protein, inositol 3-phosphate glycosyltransferase, putative colanic acid biosynthesis glycosyltransferase (all rank 77.5). Among these, L-malate glycosyltransferase initiates the biosynthesis of bacillithiol, a low-molecular-weight thiol important in detoxification of reactive oxygen species and antibiotics (121). The β-1,6-galactofuranosyltransferase and α-1,6-mannosyltransferase are involved in constructing exopolysaccharides on the cell surface similar to the EPS cluster identified in bovine strains. The polysaccharide transporter, colanic acid/amylovoran biosynthesis protein, and colanic acid glycosyltransferases support the assembly and export of an extracellular polysaccharide capsule analogous to colanic acid. In *E. coli*, colanic acid forms a gelatinous capsule that contributes to protection and colonization (122).

Ligilactobacillus salivarius habitat adaptation

Ligilactobacillus salivarius has been predominantly isolated from the digestive systems of humans, poultry, and pigs. In addition, this species has also been found in badgers and fish (92,123,124). These studies, along with the phylogenetic analysis performed in *aurora* (Figure 4), support a clear pattern of long-term host-specific adaptation.

One of the best-characterized *Lactobacillaceae* strains, *L. salivarius* UCC118, has been marketed as a probiotic for many years and is known for its anti-inflammatory and antimicrobial properties (125). Human *L. salivarius* isolates are generally associated with healthy individuals (92,126). Habitat adaptations of *L. salivarius* are thus of great interest especially adaptations to the human gut. Due to genome availability, we limited our analysis to strains from humans, poultry, and pigs. A previous study attempted to map host adaptation factors across these three hosts (127). Whereas our findings largely align with conclusions of that study, the dataset included only 34 genomes, with just 3 poultry isolates. In contrast, our expanded dataset of 302 genomes allowed for the detection of low-effect size adaptation factors.

Nisin synthesis genes (for producing an antimicrobial peptide, more correctly called a nisin variant, related to the original nisin of *Lactococcus lactis* (128)) were associated with human isolates (ranks 198.5–295). However, as their ranks suggest, these are low-effect size adaptation factors, and nisin production has also been observed in isolates from other hosts (129). There are multiple nisin variants, and it is possible that human isolates produce a unique variant, but resolving this is beyond the scope of the current study. In contrast, several high-effect size adaptation factors in *Ligilactobacillus salivarius* were associated with modifications to the bacterial surface. We identified bacillosamine biosynthesis genes (*pglA*, *pglD*, *pglC*, *pglF*; ranks 5.5). These enzymes are responsible for producing

diacetylbacillosamine-containing glycans. These structures are commonly found on surface proteins of Gram-negative pathogens (130). Such glycans can mask the bacterium from innate immune recognition. In *Campylobacter jejuni*, disruption of this glycosylation system resulted in a 10-fold reduction in natural competence (131).

We also identified numerous genes involved in the synthesis of extracellular polysaccharides (EPS), which has been shown to be crucial for habitat colonization (132). The following genes were identified as human gut-specific: glycosyltransferases RgtABCD (rank 108), rhamnosyltransferase (rank 35), UDP-N-acetylglucosamine 4-epimerase (rank 5.5), UDP-glucose 6-dehydrogenase (rank 5.5), capsular polysaccharide biosynthesis protein CpsC (rank 1), poly-β-1,6-N-acetyl-D-glucosamine synthase (PNAG synthase; ranks 88.5, 92.5), and exosortase family protein XrtG (rank 98). The production of EPS in lactic acid bacteria support biofilm formation, intestinal adhesion, and protection against host immunity and environmental stress (133). Additionally, we identified a cell wall-anchored protein with a C-terminal WxL domain (rank 57). WxL-domain proteins are known to mediate adhesion to host tissues by forming protein complexes on Gram-positive cell surfaces (134). We also found TarS/TarP (rank 47), a poly(ribitol-phosphate) β-N-acetylglucosaminyltransferase involved in wall teichoic acid modification. Altering teichoic acids can reduce immunogenicity, enhance resistance to host defences, and prevent phage infection (135).

Another human gut adaptation factor associated with bacterial surface is the peptidoglycan L,D-endopeptidase LytH (rank 17), which cleaves cross-links in the cell wall. Such enzymes are important for cell division, wall remodeling, and morphology. In additionIn addition, bacterial production of LytH has also been linked to reduced Crohn's disease symptoms in mouse models (136). In the same gene cluster, we identified a diguanylate cyclase and a c-di-GMP phosphodiesterase (ranks 92.5). Elevated c-di-GMP levels are known to trigger biofilm formation and surface adhesion. In *L. acidophilus*, introduction of a functional diguanylate

cyclase increased c-di-GMP levels, leading to enhanced EPS production and aggregation (137).

Whereas we believe this analysis represents a substantial step forward in identifying host adaptation factors in *L. salivarius*, we acknowledge that a certain degree of uncertainty remains especially in human GIT adaptation results. This is particularly evident in the annotation of several genes within the EPS biosynthesis cluster. Carbohydrate-modifying enzymes are especially prone to annotation discrepancies (138), and this limitation is exemplified by the gene *mshA*. We identified six copies of *mshA* in human gut strains (ranks: 5.5, 10, 21, 33, 43, 66). These genes were annotated as L-malate glycosyltransferases using KofamScan (which uses KEGG as a reference database), whereas *prokka* annotated them as D-inositol-3-phosphate glycosyltransferases. If the KEGG annotation is correct, *mshA* participates in bacillithiol biosynthesis (139). If the *prokka* annotation is correct, the gene is instead involved in mycothiol biosynthesis (140). Both thiols play protective roles by shielding intracellular proteins and enzymes from oxidative damage. However, it remains unclear which of these substances is utilized by *L. salivarius*.

We also identified genes encoding three CRISPR-associated nucleases as putative human gut adaptation factors: *cas1* (rank 58), *cas2* (rank 52), and *cas9* (rank 36) as well as Csa3 (CRISPR-associated protein; rank 82). CRISPR-Cas systems are common in lactobacilli and have been linked to survival in highly competitive or densely populated environments (141). In addition, we found numerous genes involved in carbohydrate utilization. These include genes encoding a beta-glucoside PTS system, beta-D-glucosidase, galactiol PTS system, oligo-1,6-glucosidase, and a mannose PTS system. These genes likely expand the metabolic flexibility of *L. salivarius*, enabling it to access a wider range of food and host-derived carbohydrates. The functional significance of these genes can be illustrated by the mannose PTS system (ranks 100.5–130.5). This system allows *L. salivarius* to import and metabolize

mannose, a sugar commonly found in mucosal glycoproteins of the host gut lining (142). This confers a nutritional advantage within the gastrointestinal tract. Deletion of mannose PTS genes in a related *Lactobacillus* species was shown to significantly reduce its gut residence time (143).

We also identified genes involved in methionine acquisition: *metN*, *metP*, and *metQ* (ranks 64.5–68.5), which were co-localized with succinyl-diaminopimelate desuccinylase (DapE, rank 64.5). DapE is a key enzyme in the lysine biosynthesis pathway, producing lysine and meso-diaminopimelate, both essential components for bacterial peptidoglycan synthesis (144). Like many lactic acid bacteria, *L. salivarius* is auxotrophic for methionine (145), so the acquisition of methionine may be critical for survival in human gut. We also identified genes related to alanine utilization: an alanine permease (rank 62) and alanine dehydrogenase (rank 95). Alanine dehydrogenase enables bacteria to use alanine as a sole nitrogen source and is important for sustaining growth under anaerobic conditions (146).

In porcine isolates, the top-ranked gene identified by *aurora* was an IS110 family transposase, with another transposase ranked 12th. Several phage-associated genes also appear among the top results. Among the lower-effect size clusters (present in ~30% of porcine isolates), we identified genes encoding enzymes for L-rhamnose and L-fucose metabolism: L-rhamnulokinase, L-fucose mutarotase, L-fucose isomerase, and L-fuculose phosphate aldolase (ranks 50). These enzymes are predicted to allow *L. salivarius* to metabolize sugars derived from plant fibers and host mucins. It was shown that utilization of mucin-derived fucose provides a competitive advantage in the gut environment. An example of this is *Klebsiella pneumoniae* where fucose metabolism was linked to enhanced growth and biofilm formation (147).

We also identified a bacteriocin (rank 4) and its associated transporter (rank 5), suggesting antimicrobial activity that could contributes to niche competition. Additionally, we detected the tetracycline resistance gene *tetM* (rank 44). Tetracyclines are widely used in pig farming, and resistance genes are well-documented in pig-derived microbiota (148). Two other notable porcine-associated genes were argininosuccinate lyase (rank 7) and argininosuccinate synthase (rank 11), involved in arginine biosynthesis. These genes may make *L. salivarius* prototrophic for arginine, or alternatively, provide arginine for the arginine deiminase system. In *Streptococci*, this system contributes to external pH regulation and may play a similar role in the porcine isolates of *L. salivarius* (149). However, functionality of this system in *L. salivarius* has not yet been experimentally shown. The first enzyme of this pathway was identified as a bovine colonization factor in the related *Ligilactobacillus*, *L. ruminis* which suggests a broader distribution of this system in lactobacilli (see above).

We also identified another enzyme potentially involved in acid stress resistance: teichoic acid D-alanyltransferase (rank 10). This enzyme modifies the bacterial cell wall, a key mechanism for tolerating acidic environments and resisting cationic antimicrobial peptides (150). Finally, ornithine decarboxylase (rank 54) was also identified as porcine colonization factor. This enzyme decarboxylates ornithine to putrescine, a process that consumes protons and contributes to pH homeostasis. This amino acid decarboxylation pathway is a known acid resistance mechanism in *Lactobacillus rossiae* (151).

Just as in human adaptation, multiple genes related to exopolysaccharide (EPS) synthesis were identified in *L. salivarius* as porcine adaptation factors. These include the exopolysaccharide production protein ExoZ (rank 125) and several co-localized glucosyl and glycosyltransferases (ranks 27–65.5), along with a capsular biosynthesis protein CpsJ (rank 56). These genes likely serve roles similar to those in human isolates, contributing to adhesion and immune evasion. Some of the glycosyl and glucosyltransferases were also co-

localized with components of the protein translocation system SecA2-SecY2 (ranks 64–121.5). This system was previously shown to be essential for rodent colonization by *L. reuteri* and is responsible for translocating a large surface adhesin (5,6,42). In previous studies, the system has also been identified in *L. salivarius* pig and poultry isolates, but not in human isolates (152). In agreement with this observation in our dataset, the SecA2-SecY2 system was present in 89% of porcine isolates and 80% of poultry isolates, but only 2% of human isolates.

Whereas the surface adhesin secreted by the SecA2-SecY2 system has not been identified in porcine isolates of *L. salivarius*, we identified two potential candidates. One is a fibronectin-binding protein (rank 60), co-localized with the SecA2-SecY2 components and associated glycosyltransferases. However, this protein lacks the serine-rich repeat region typical of other adhesins (5). Another promising candidate is a large (~5600 bp) mucin adhesion protein (rank 2), found in 82% of porcine isolates and absent from isolates from all other hosts. This gene is co-localized with arginine biosynthesis genes, ExoZ, and chologlycine hydrolase (rank 3). Chologlycine hydrolase, also known as bile salt hydrolase, detoxifies bile by cleaving conjugated bile acids, enhancing bile resistance (153). These three high-effect size co-localized genes could thus be essential porcine colonization factors. Additionally, we identified a collagen-like protein (rank 13), which is not co-localized with any other significantly porcine-associated genes. Collagen-like proteins are frequently found in pathogens and are known to bind to host fibronectin or collagen (154).

Although the porcine isolates are related to the poultry strains (Figure 4), the general profile of poultry adaptation in *L. salivarius* mirrors that of porcine isolates. The adaptation gene set is similarly dominated by glycosyltransferases, EPS biosynthesis proteins, and components of the SecA2-SecY2 protein translocation system. Some adaptation factors, such as SecY2, are shared between porcine and poultry isolates. Others appear specific to poultry, including

glycosyltransferase EpsJ (rank 5), peptidoglycan L,D-endopeptidase (rank 13), and biofilm protein TabA (rank 15). It has been previously observed that genes involved in EPS synthesis and other extracellular structures underwent host-specific diversification under selective pressure in *L. salivarius* (155). Some of these genes thus may fulfil the same role in porcine and poultry strains, but have very divergent sequence. A proteomic analysis could further clarify how functional modifications in these proteins support host-specific adaptation in poultry, pigs, and humans. Similar to porcine isolates, poultry isolates also contain several unique adhesion proteins, some of which may be exported by the SecA2-SecY2 system. One such candidate is fibronectin-binding protein A (rank 54), whereas another is a protein with an LPXTG motif (rank 1). The latter is genetically co-localized with some of the poultry-specific components of the SecA2-SecY2 system

We identified several poultry-specific adaptation factors with unique functional roles. One such gene encodes L-lactate oxidase, an enzyme that oxidizes lactic acid to pyruvate whereas generating hydrogen peroxide (H_2O_2) as a byproduct. H_2O_2production is a well-established probiotic trait known to inhibit competing microbes. Only *Lactobacillus* strains with intact H_2O_2-generating capabilities have been shown to effectively protect their hosts and exclude intestinal pathogens (156,157). Another notable poultry-associated gene is sialidase (rank 22), which plays a role in cleaving sialic acid residues from host glycoconjugates. Sialic acid metabolism has already been shown to be essential for *L. salivarius* colonization of the piglet gut (158). However, the contribution of sialidase to colonization of the poultry gut has not yet been experimentally validated. This gene is present in 44% of poultry isolates but is nearly absent in isolates from other hosts. Another poultry colonization factor is oxidoreductase YjhC (rank 22), a gene involved in the downstream catabolism of sialic acid following its release by sialidase (159). The presence of both genes suggests that poultry-adapted *L. salivarius* strains may exploit host-derived sialic acids as a nutrient source.

Lastly, we identified several genes annotated as bacteriocins, including plantaricin alpha-peptide (rank 63), bacteriocin lactocin-F (rank 81), mutacin IV (rank 81), and a general bacteriocin gene (rank 127). Bacteriocin production has previously been observed in poultry-derived *L. salivarius* strains (160) and is widely recognized as a probiotic trait. Bacteriocins can support niche establishment by directly inhibiting pathogenic bacteria, preventing colonization by competing strains, and modulating the composition of the host microbiota (161).

Lactobacillus crispatus habitat adaptation

Unlike many other *Lactobacillaceae* species, *L. crispatus* has been the subject of multiple investigations into habitat adaptation mechanisms (162–165). However, these studies have generally lacked sufficient statistical power to detect adaptation factors with small effect sizes. The largest previous study included only 105 *L. crispatus* genomes (166), whereas herein we analyze 596 genomes. Moreover, earlier analyses relied exclusively non-GWAS statistical analyses, which is susceptible to sampling bias and may obscure subtle adaptive signals. To our knowledge, this is the first genome-wide association study (GWAS) conducted on *L. crispatus*.

We begin by examining the strains of *L. crispatus* isolated from the human gastrointestinal tract. The GWAS analysis revealed 41 genes present in human gut isolates and entirely absent from strains associated with poultry and the human urogenital system. Among these gut-specific genes is a β-glucuronidase (rank 21), which is co-localized with genes involved in ribose uptake and metabolism (ranks 108). The ribose transport and catabolic genes enable *L. crispatus* to utilize ribose, potentially derived from dietary fibers or extracellular nucleotides,

as a nutrient source. Access to this sugar may confer a competitive advantage in the gut, as demonstrated in *Bacteroides*, where a ribose-utilization system was shown to be essential for effective intestinal colonization (167). Notably, a β-glucuronidase gene has also been identified in the human-derived *Lactobacillus gasseri* strain ADH (168). In *L. crispatus*, β-glucuronidase likely facilitates the deconjugation of host-derived glucuronides, such as those in bile or mucins, thereby releasing glucuronic acid as a utilizable carbon source.

Adjacent to this genomic region is a cluster encoding the full purine biosynthesis operon (PurF, PurQ, PurL, PurS, PurC, PurM, PurE, PurK, and PurH; ranks 21), as well as formate–tetrahydrofolate ligase (rank 21). The ability to synthesize purines *de novo* may enable *L. crispatus* to survive in environments where free purines are limited. Supporting this, *Escherichia coli* mutants deficient in purine biosynthesis exhibited a >10,000-fold reduction in intestinal colonization efficiency in mice (169). The importance of purine biosynthesis for gut colonization has also been observed in several enteric pathogens (170). Additionally, formate–tetrahydrofolate ligase enables assimilation of formate into the one-carbon pool via synthesis of 10-formyl-tetrahydrofolate, a crucial cofactor in purine biosynthesis and other folate-dependent processes. This function may allow *L. crispatus* to exploit formate as an anabolic substrate. Consistently, formate–tetrahydrofolate ligase is among the most highly expressed enzymes in the human gut microbiomes (171). The gene clusters for ribose metabolism and purine biosynthesis appear to be functionally linked. In the purine biosynthetic pathway, the initial step involves the conversion of ribose-5-phosphate (derived from the pentose phosphate pathway) into 5-phosphoribosyl-1-pyrophosphate. Correspondingly, the pentose phosphate pathway gene ribulose-3-phosphate epimerase (rank 108) is co-localized with the purine biosynthesis genes, and additional pentose phosphate pathway genes, such as two transketolase genes (ranks 21 and 80.5), are also enriched in human gut isolates.

Beyond ribose and glucuronide metabolism, human gut isolates harbor a range of other carbohydrate utilization genes. A notable gene cluster contains β-N-acetylhexosaminidase (rank 21), a sugar efflux protein (rank 21), and a glucoside hydrolase (rank 52). β-N-acetylhexosaminidase facilitates the hydrolysis of β-linked N-acetylglucosamine residues, which are common in human gut mucins and other glycoproteins (172). An additional operon involved in β-glucoside catabolism (bglFGH; ranks 183.5) is present in 83% of human gut-derived strains and absent from isolates of other ecological origins.

In addition to the major colonization factors described above, our analysis identified several other genetic elements potentially contributing to *L. crispatus* adaptation to the human urogenital system. Multiple CRISPR–Cas elements were detected, including CRISPR-associated proteins and the nucleases Cas2, Cas3, Cas5, and Cas6 (ranks 80.5). We also identified a complete glycine/betaine/carnitine/choline transport system (opuCAB, osmX; rank 52), which is known to support bacterial tolerance to osmotic stress (173).

Several clusters of cell wall and surface glycan biosynthesis genes were also present. One cluster included genes for UDP-2-acetamido-4-amino-2,4,6-trideoxy-α-D-galactose:2-oxoglutarate ligase, D-alanine–D-alanine ligase, aspartate aminotransferase, and L-amino acid–D/L-Glu epimerase (ranks 80.5), all implicated in the synthesis of components that are essential for adhesion to intestinal mucosa (174). Another cluster encoded cell wall synthesis genes, including UDP-D-galactose:(glucosyl)LPS α-1,6-D-galactosyltransferase (WaaB, rank 136.5). WaaB has previously been shown to be essential for *Salmonella Typhimurium* invasiveness (175). In addition, a pediocin bacteriocin gene (rank 21) was identified, although it was not co-localized with other adaptation factors.

A larger gene cluster contained genes with diverse functions. Some are involved in cell wall modification (e.g., teichoic acid D-Ala hydrolase, rank 21), whereas others contribute to

stress regulation through modulation of inorganic phosphate metabolism via the PPX/GPPA system (rank 52). This system has been shown to be essential for biofilm formation, stress response, and serum resistance in *Campylobacter jejuni* (176). Within the same cluster, we also identified choloylglycine hydrolase (bile salt hydrolase, rank 52), a known human colonization factor. Its role in the gut environment is discussed above.

Finally, a large gene cluster strongly associated with the gastrointestinal tract is enriched for carbon catabolism pathways. This cluster includes genes for the catabolism of glycerol, xylan, cellobiose, galactosides, glucosides, and rhamnose (including α-L-rhamnosidase), alongside transketolases (ranks 21 and 80.5) and a peptidase (rank 21). These features suggest a broad metabolic capacity to exploit diverse host- and diet-derived carbohydrates.

In additionIn addition, a higher proportion of urogenital system (UGS)-associated genes passed the MAGs test compared with gut-associated genes (Figure S9). Among the top 100 UGS-associated genes, 34% passed the test, whereas only 2% of the top 100 gut-associated genes did. This discrepancy likely reflects the large number of allochthonous strains identified within the gut-derived *L. crispatus* population (Figure 4). One large cluster of UGS-associated genes contained multiple loci that passed the MAGs test. These included genes for a magnesium ATPase (rank 1), ammonium transporter (rank 14), fibronectin-binding protein (rank 2), cell wall-anchored thioredoxin (rank 75.5), lactococcin G transport protein (rank 94), a bacteriocin-like peptide (rank 52), glucosyl hydrolase/xylohydrolase (rank 24), and a lantibiotic transport system (three genes; ranks 33.5, 40.5, and 45). Additionally, two helveticin-like genes (ranks 13 and 59), an S-layer protein (rank 26), zinc/manganese transporters (ranks 65 and 90), and a toxin–antitoxin system (rank 99.5) were identified within the same cluster.

The analysis of *L. crispatus* adaptation to the human urogenital system revealed several previously described colonization factors. For example, a gene cluster containing pullulanase (rank 28), a chloride channel (rank 21), two gene copies for a surface layer protein A (rank 48.5), a peptidoglycan-binding protein (rank 54), an oligopeptide transporter (rank 18.5), and a sodium/hydrogen antiporter (rank 6). Pullulanase is a well-established colonization factor that enables *L. crispatus* to degrade host glycogen, abundant in vaginal fluid, into maltodextrins, which can then be fermented into lactic acid (177). Similarly, S-layer proteins are known to promote colonization by forming a protective surface layer and mediating adhesion to vaginal epithelial cells (178). The peptidoglycan-binding protein likely promotes auto-aggregation, a mechanism previously linked to colonization in *L. crispatus* through aggregation-promoting factor and related genes (179). The oligopeptide transporter likely supports growth in the peptide-rich vaginal environment by importing small peptides as nitrogen and amino acid sources (180). Finally, the chloride channel and sodium/hydrogen antiporter likely contribute to acid tolerance, helping *L. crispatus* survive at the low pH (~4) characteristic of the healthy vaginal environment by preventing intracellular acidification (181).

Unexpectedly, we also identified two genes involved in riboflavin (vitamin B2) biosynthesis: riboflavin synthase (rank 12) and ribD (rank 10). Riboflavin production is regarded as a niche-specific adaptation supporting lactobacilli survival in host-associated habitats (182). As riboflavin availability has been linked to female urogenital and reproductive health, this biosynthetic capability may represent a probiotic trait (183). Riboflavin biosynthesis has also been observed in other vaginal *Lactobacillaceae*, including *Lactiplantibacillus plantarum* and *Limosilactobacillus reuteri* (184).

In addition, we identified cysteine desulfurase (rank 85) and several Suf system proteins (SufU, SufT, SufB, SufC ATP-binding protein, and SufD; ranks 85–102), which are involved

in iron–sulfur (Fe–S) cluster assembly and repair under oxidative stress. This function is particularly relevant in the vaginal environment, which is rich in hydrogen peroxide – much of it produced by *L. crispatus* itself to inhibit pathogens. By maintaining Fe–S cofactors under oxidative stress, the Suf pathway ensures continued metabolic activity (185). Indeed, Suf system genes were previously proposed as putative *L. crispatus* vaginal colonization factors (186). Finally, we identified a manganese transport protein (rank 91), which likely contributes to oxidative stress resistance by importing Mn^{2+}. Manganese serves as an antioxidant in lactobacilli, and Mn^{2+} uptake has been shown to be essential for aerobic growth and peroxide tolerance (187).

We identified multiple adhesion proteins and components involved in surface display. In addition to the previously described S-layer protein, two fibronectin-binding proteins (ranks 25 and 27) and a clumping factor B homolog (rank 3; analogous to the adhesion factor ClfB in *Staphylococcus aureus*) were detected. Furthermore, a surface display machinery spanning ranks 62–73 was identified, consisting of a Firmicutes-CTERM sorting domain-containing protein, an exosortase family protein (XrtG), and a corresponding XrtG-associated glycosyltransferase. This system likely facilitates colonization by enabling proper display of adhesive or protective proteins on the bacterial surface. Mechanistically, it is analogous to the sortase–LPXTG anchoring pathway where sortase cleaves LPXTG-motif proteins and covalently attaches them to the cell wall (188). In *L. crispatus*, the presence of such machinery suggests a specialized system for processing and tethering surface proteins, comparable to the SecA2-SecY2 translocation system described in *L. reuteri* and *L. salivarius* (see above).

Genes encoding diguanylate cyclase (rank 72) and an EAL domain-containing phosphodiesterase (rank 63) were identified. These proteins likely regulate biofilm formation

and community behavior through the synthesis and degradation of cyclic-di-GMP, as previously demonstrated in *Lactobacillus acidophilus* (189).

We identified a cluster of L-ascorbate (vitamin C) catabolic and transport genes (ranks 36–45). This cluster contained the complete ulaABCDEFGR operon along with additional catabolic genes, including L-ribulose-5-phosphate 3-epimerase (rank 43), L-ribulose-5-phosphate 4-epimerase (rank 38.5), and xylulose-5-phosphate/fructose-6-phosphate phosphoketolase (rank 39). These genes enable *L. crispatus* to utilize L-ascorbate as an unconventional substrate, a capability previously observed in *L. crispatus* vaginal isolates (190).

Finally, three proteases were identified: a cysteine proteinase (rank 87.5), a pyrrolidone-carboxylate peptidase (rank 6), and a peptidoglycan endopeptidase (rank 87.5). These proteases have been described in *Lactobacillus helveticus* isolated from kefir (191). The extracellular cysteine protease may degrade host proteins such as mucins in vaginal mucus, liberating peptides and amino acids as nutrients. Pyrrolidone-carboxylate peptidase can remove N-terminal pyroglutamate residues from host peptides, increasing their bioavailability (192). The peptidoglycan endopeptidase functions as an autolysin, essential for cell wall remodeling during division and biofilm maturation. In other Gram-positive bacteria, autolysin deficiency leads to impaired cell separation and attenuated virulence (193).

Among the poultry-associated genes, we identified multiple putative surface adhesins, consistent with previous observations that *L. crispatus* isolates from poultry (particularly chickens) harbor specific cell surface proteins (194). These included an S-layer protein (rank 35) co-localized with a mucin-binding protein (rank 25), another S-layer protein co-localized with a bacteriocin (ranks 60), adhesin (rank 19), two co-localized S-layer proteins (ranks 63 and 99), and an additional S-layer protein (rank 88.5). We also identified a CAAX-family

protease (rank 62), which likely processes surface proteins to ensure proper assembly and functionality of adhesion factors. In addition, several of these adhesins appear to be non-essential for poultry colonization. For example, the S-layer protein ranked 99 was present in only 22% of poultry isolates (20 strains) and was are in human gut (5 strains) and urogenital system (27 strains) isolates. Likewise, the S-layer protein ranked 63 was detected in only 12% of poultry isolates and was nearly absent in other habitats. Whereas adhesion factors typically show strong effect sizes in our analysis, these poultry-associated adhesins displayed weaker effects, which we attribute to the heterogeneous nature of the “poultry” category, encompassing multiple niches. Indeed, our dataset includes isolates from both turkeys and chickens, and prior work has reported species-specific adaptation genes in *L. crispatus* (194).

We also identified multiple genes related to antimicrobial resistance and metal tolerance, including two polyether ionophore transporters (rank 38.5), a tetracycline repressor (rank 78.5), a tetracycline resistance protein TetW (rank 20.5), and multidrug efflux pumps (ranks 16 and 20.5). Such genes likely support survival under antimicrobial pressures in poultry environments. Ionophore antibiotics (e.g., salinomycin, narasin) are widely used as coccidiostats in poultry feed (195), and copper is frequently supplemented as a growth promoter (196). Accordingly, we detected a copper-exporting ATPase and an associated repressor (ranks 66.5 and 72.5). The carriage of TetW and its repressor further supports resistance to tetracyclines, which may be present in feed or the environment. Consistent with this, a Na^+-driven multidrug efflux pump was enriched in chicken-derived *L. crispatus* strains.

Genes involved in stress tolerance were also identified, including a Na^+/H^+ antiporter (rank 4.5), a peroxidase (rank 74), and two co-localized lipoproteins: one of unknown function (rank 1) and an osmotically inducible lipoprotein OsmE (rank 2.5). Na^+/H^+ antiporters are critical for maintaining intracellular pH and ion balance under acidic or saline conditions.

Osmotically inducible lipoproteins such as OsmE, and possibly the adjacent lipoprotein, likely stabilize the cell envelope under dehydration or high-solute stress (197). The identified peroxidase provides defence against oxidative stress by detoxifying reactive oxygen species (198).

In contrast, we identified fewer genes linked to nutrient utilization. Notable exceptions included scyllo-inositol 2-dehydrogenase (rank 2.5; for catabolism of inositol and related polyols), β-glucoside kinase (rank 17.5; for utilization of plant-derived β-glucosides, previously reported in poultry isolates; (199)), pyroglutamyl-peptidase (rank 4.5; releases amino acids from pyroglutamate-capped peptides, a colonization factor shared with human UGS isolates), and formate dehydrogenase (rank 66.5; oxidizes formate to generate energy under anaerobic conditions). Additionally, a galactitol PTS system (rank 49.5) co-localized with L-ribulose-5-phosphate 4-epimerase (rank 49.5) and polysaccharide biosynthesis protein PslJ (rank 49.5) was identified, though this locus was present in only 4% of poultry isolates. Galactitol, derived from galactose reduction, can result from the enzymatic breakdown of galacto-oligosaccharides (GOS), which was found to stimulate the growth of *Lactobacillus johnsonii* in chickens (200). We also identified α-D-xyloside xylohydrolase (rank 8) co-localized with a starch-binding SusE/F protein (rank 92), suggesting capacity for degradation of xylosides, xylan, or starch into utilizable sugars.

Finally, we identified a zinc/manganese transport system (rank 23). Although distinct from the manganese transport system detected in vaginal isolates, its likely role is similar – providing manganese for superoxide dismutase, a key enzyme for oxidative stress defence (201).

Limosilactobacillus mucosae habitat adaptation

Limosilactobacillus mucosae is a Gram-positive, heterofermentative lactic acid bacterium originally isolated from the intestinal mucosa of piglets (202). This species has attracted attention for its probiotic potential, with reported health-promoting effects including enhancement of mucosal immunity and cholesterol-lowering activity (203–205). It is now recognized as a natural inhabitant of the gastrointestinal mucosa in multiple hosts, including pigs, cattle, and humans (206). However, this is the first (GWAS) study performed for *L. mucosae*. A comparative analysis of 101 strains has been published, but the limited number of pig and bovine isolates prevented identification of host-specific genetic determinants (207).

Compared with other species analyzed here, *L. mucosae* appears to harbor fewer high effect size host-adaptation factors. The top human-associated gene was a peptidoglycan-binding domain protein (LysM), present in 62% of human isolates, 9% of bovine isolates, and absent in porcine isolates. This gene was co-localized with a general secretion pathway protein M (rank 10) and an alanine-adding enzyme (rank 9). Together, these genes may contribute to D-alanylation of teichoic acids, a modification known to be critical for gut colonization in *Lactobacillus reuteri* (208).

We also identified a complete tryptophan biosynthesis operon (TrpABCDEFG; ranks 100–174) co-localized with a sensor histidine kinase (rank 43.5). This operon enables *L. mucosae* to synthesize tryptophan de novo, which may confer a competitive advantage in the human gut, where most colonizers lack this capability (209). Beyond nutritional benefits, tryptophan-derived metabolites influence host physiology and microbiota cross-talk (210).

Genes associated with carbohydrate catabolism were also detected. A galactitol PTS transport system (ranks 28–42) was present in 42% of human isolates and 27% of bovine isolates whereas being absent in porcine isolates. Galactitol metabolism has been shown to be crucial

in poultry colonization by other *Lactobacillaceae* strains and may also be relevant for *L. mucosae* colonization of human gut (200). In addition, we identified an ascorbate PTS system (ranks 48–94.5) co-localized with a transketolase gene (rank 94.5). Although ascorbate catabolism via the *ula* operon produces D-xylulose-5-phosphate, a substrate of transketolase, this arrangement suggests a coordinated pathway for ascorbate utilization. A D-xylose transporter (rank 27) was also identified as a human-associated gene.

Several bacteriocin and immunity-related genes were enriched in human isolates, including a bacteriocin ATP transport system (rank 20), a bacteriocin peptide gene (rank 49), CRISPR-associated endonuclease Cas9 (rank 123.5), and a linearomycin/streptolysin S transport system (rank 8). Additional genes linked to cell surface modifications were detected, including O-antigen transport permease (rank 2), O-antigen biosynthesis α-1,2-rhamnosyltransferase (rank 4), and EPS-related UDP-N-acetylglucosamine 2-epimerase (rank 59). However, that most *L. mucosae* strains lack the chain-length-determining protein Cld, suggesting limited EPS biosynthesis capability (211).

Finally, we identified a cluster of genes involved in glycogen metabolism, including glycogen phosphorylase, glycogen synthase, two glucose-1-phosphate adenylyltransferases, and a 1,4-α-glucan branching enzyme (ranks 52–283). Glycogen synthesis and storage provide a metabolic reserve that supports bacterial survival during nutrient scarcity, therefore promoting persistence in the gut (212).

We identified several bovine-specific colonization factors, including a mucin-binding protein (rank 34). However, unlike similar genes in *L. reuteri* and *L. salivarius*, this is a low effect size gene present in just 27% of bovine and 19% of human isolates whereas being absent in porcine isolates. A tryptophan/tyrosine transport system (rank 10.5) was also detected. This suggests possible tryptophan auxotrophy in bovine strains. Co-localized with this transporter

was succinyl-diaminopimelate desuccinylase, a lysine biosynthesis enzyme also found adjacent to methionine acquisition genes in human-adapted *L. salivarius* (see above).

We identified a cluster of carbohydrate-degrading enzymes: β-xylosidase, α-L-arabinofuranosidase, and a glycoside/pentoside/hexuronide symporter (ranks 1.5 to 8.5) – likely enabling degradation and uptake of complex plant polysaccharides (e.g., hemicellulose components) abundant in bovine diets. A maltose/malto-oligosaccharide transporter (rank 16) and maltogenic α-amylase (rank 12) were also present in a separate genetic cluster, indicating capacity for maltodextrin utilization. Additionally, antimicrobial defence elements such as lysozyme (rank 28.5) and glutathione reductase (rank 17) were identified. The latter contributes to oxidative stress resistance (213).

In porcine *L. musocase* isolates, multiple collagen-like proteins were found: two co-localized (ranks 11 and 81) and one separate (rank 89). The co-localized adhesins were adjacent to UDP-N-acetylglucosamine 2-epimerase (rank 2) and a glycosyltransferase (rank 97.5), implicating these in EPS synthesis and host adhesion. Further, an arabinogalactan/maltooligosaccharide transport system, mannose-6-phosphate isomerase, and a co-localized transcriptional repressor (rank 26.5) were detected. These genes are predict to broaden the carbohydrate substrate repertoire.

We also uncovered a stress-response related gene cluster comprising universal stress protein F (rank 12), ferredoxin (rank 81), and enzymes mediating ADP-ribosylation (both ADP-ribosyltransferase and ADP-ribose hydrolase, ranks 81). ADP-ribosylation systems regulate DNA damage responses, viral infection, and intracellular signalling (214). Finally, an acetoin permease, implicated in acid resistance, was identified as a putative porcine colonization factor (215).

Leuconostoc mesenteroides/pseudomesenteroides habitat adaptation

L. mesenteroides/pseudomesenteroides was the only species of Lactobacillaceae adapted to both food and human gut. The majority of the *L. mesenteroides/pseudomesenteroides* isolates were isolated from dairy (n=190) followed by edible plants (n=67) and then human gut (n=11). In addition, unlike other species (Figure 3C) the human gut isolates do not cluster in one lineage (Figure 4). The strains are present in close phylogenetic proximity to both the plant isolates and the dairy isolates (Figure 4). The top ranked human gut colonization factor (LPXTG surface adhesin) is spread around the phylogenetic tree and is also encoded by the genomes of four dairy and one plant isolate. Thus, edible plants and dairy can potentially be sources of human-adapted *Leuconostoc mesenteroides/pseudomesenteroides* strains. However, although only a minority of food isolates encode the adhesin the proportion of human-adapted strains in food is likely very low. It is also evident from the *aurora* analysis of the *L. pseudomesenteroides/mesenteroides* genomes that the dairy isolates are clearly distinguishable from the plant isolates (Figure S11). The highest ranking dairy colonization factors were multiple genes encoding beta-galactosidases and lactose permease. Other low effect size genes were copper-exporting P-type ATPase and multicopper oxidase. In addition to these there were multiple mobile genetic elements in the results indicating that these genes may be mobile. Two top plant colonization factors were two genes related to iron export (Figure S11B. The tryptophan synthesis operon *trpABCDEF* found to be a low effect size plant colonization factor.

Whereas adaptation of lactobacilli to different food types was not specifically investigated, we observed that specialization to different foods is not common in *Lactobacillaceae* as only species isolated from mammalian hosts were predicted to be habitat-specialized (Figure 3C).

L. pseudomesenteroides/*mesenteroides* is however different even in this regard because the plant and dairy isolates clearly form separate clusters in the phylogenetic tree (Figure 2C) – a strong indication of specialization.

References

1. Bujdoš D, Walter J, O'Toole PW. aurora: a machine learning gwas tool for analyzing microbial habitat adaptation. Genome Biol. 2025 Mar 23;26(1):66. doi:10.1186/s13059-025-03524-7

2. Saber MM, Shapiro BJ. Benchmarking bacterial genome-wide association study methods using simulated genomes and phenotypes. Microbial Genomics. 2020 Mar 1;6(3). doi:10.1099/mgen.0.000337

3. Hammond RK, Pahl MC, Su C, Cousminer DL, Leonard ME, Lu S, et al. Biological constraints on GWAS SNPs at suggestive significance thresholds reveal additional BMI loci. eLife. 2021 Jan 18;10. doi:10.7554/elife.62206

4. LaPierre N, Taraszka K, Huang H, He R, Hormozdiari F, Eskin E. Identifying causal variants by fine mapping across multiple studies. Zeggini E, editor. PLoS Genet. 2021 Sep 20;17(9):e1009733. doi:10.1371/journal.pgen.1009733

5. Cinar MS, Niyas A, Avci FY. Serine-rich repeat proteins: well-known yet little-understood bacterial adhesins. Federle MJ, editor. J Bacteriol. 2024 Jan 25;206(1):e00241-23. doi:10.1128/jb.00241-23

6. Latousakis D, Nepravishta R, Rejzek M, Wegmann U, Le Gall G, Kavanaugh D, et al. Serine-rich repeat protein adhesins from *Lactobacillus reuteri* display strain specific glycosylation profiles. Glycobiology. 2019 Jan 1;29(1):45–58. doi:10.1093/glycob/cwy100

7. Frese SA, Benson AK, Tannock GW, Loach DM, Kim J, Zhang M, et al. The Evolution of Host Specialization in the Vertebrate Gut Symbiont Lactobacillus reuteri. Guttman DS, editor. PLoS Genet. 2011 Feb 17;7(2):e1001314. doi:10.1371/journal.pgen.1001314

8. Roos S, Aleljung P, Robert N, Lee B, WadstrÃ¶m T, Lindberg M, et al. A collagen binding protein from *Lactobacillus reuteri* is part of an ABC transporter system? FEMS Microbiology Letters. 1996 Oct;144(1):33–8. doi:10.1111/j.1574-6968.1996.tb08505.x

9. Beyvers S, Jelonek L, Goesmann A, Schwengers O. Bakta Web - rapid and standardized genome annotation on scalable infrastructures. Nucleic Acids Res. 2025 Jul 7;53(W1):W51–6. doi:10.1093/nar/gkaf335 PubMed PMID: 40271661; PubMed Central PMCID: PMC12230652.

10. Li F, Li X, Cheng CC, Bujdoš D, Tollenaar S, Simpson DJ, et al. A phylogenomic analysis of Limosilactobacillus reuteri reveals ancient and stable evolutionary

relationships with rodents and birds and zoonotic transmission to humans. BMC Biol. 2023 Mar 13;21(1):53. doi:10.1186/s12915-023-01541-1

11. Walter J. Ecological Role of Lactobacilli in the Gastrointestinal Tract: Implications for Fundamental and Biomedical Research. Appl Environ Microbiol. 2008 Aug 15;74(16):4985–96. doi:10.1128/AEM.00753-08

12. Walter J, Britton RA, Roos S. Host-microbial symbiosis in the vertebrate gastrointestinal tract and the *Lactobacillus reuteri* paradigm. Proc Natl Acad Sci USA. 2011 Mar 15;108(supplement_1):4645–52. doi:10.1073/pnas.1000099107

13. Paone P, Cani PD. Mucus barrier, mucins and gut microbiota: the expected slimy partners? Gut. 2020 Dec;69(12):2232–43. doi:10.1136/gutjnl-2020-322260

14. Oh PL, Benson AK, Peterson DA, Patil PB, Moriyama EN, Roos S, et al. Diversification of the gut symbiont *Lactobacillus reuteri* as a result of host-driven evolution. The ISME Journal. 2010 Mar 1;4(3):377–87. doi:10.1038/ismej.2009.123

15. Duar RM, Lin XB, Zheng J, Martino ME, Grenier T, Pérez-Muñoz ME, et al. Lifestyles in transition: evolution and natural history of the genus Lactobacillus. FEMS Microbiology Reviews. 2017 Aug 1;41(Supp_1):S27–48. doi:10.1093/femsre/fux030

16. Frese SA, MacKenzie DA, Peterson DA, Schmaltz R, Fangman T, Zhou Y, et al. Molecular Characterization of Host-Specific Biofilm Formation in a Vertebrate Gut Symbiont. Garsin DA, editor. PLoS Genet. 2013 Dec 26;9(12):e1004057. doi:10.1371/journal.pgen.1004057

17. Gänzle MG, Zheng J. Lifestyles of sourdough lactobacilli – Do they matter for microbial ecology and bread quality? International Journal of Food Microbiology. 2019 Aug;302:15–23. doi:10.1016/j.ijfoodmicro.2018.08.019

18. Su MSW, Oh PL, Walter J, Gänzle MG. Intestinal Origin of Sourdough *Lactobacillus reuteri* Isolates as Revealed by Phylogenetic, Genetic, and Physiological Analysis. Appl Environ Microbiol. 2012 Sep 15;78(18):6777–80. doi:10.1128/AEM.01678-12

19. Wilson CM, Loach D, Lawley B, Bell T, Sims IM, O'Toole PW, et al. Lactobacillus reuteri 100-23 Modulates Urea Hydrolysis in the Murine Stomach. Macfarlane GT, editor. Appl Environ Microbiol. 2014 Oct;80(19):6104–13. doi:10.1128/AEM.01876-14

20. Eitinger T, Suhr J, Moore L, Smith JAC. Secondary Transporters for Nickel and Cobalt Ions: Theme and Variations. Biometals. 2005 Aug;18(4):399–405. doi:10.1007/s10534-005-3714-x

21. Brooijmans R, Smit B, Santos F, Van Riel J, De Vos WM, Hugenholtz J. Heme and menaquinone induced electron transport in lactic acid bacteria. Microb Cell Fact. 2009 Dec;8(1):28. doi:10.1186/1475-2859-8-28

22. Liu Y, Charamis N, Boeren S, Blok J, Lewis AG, Smid EJ, et al. Physiological Roles of Short-Chain and Long-Chain Menaquinones (Vitamin K2) in Lactococcus cremoris. Front Microbiol. 2022 Mar 15;13:823623. doi:10.3389/fmicb.2022.823623

23. Duwat P, Sourice S, Cesselin B, Lamberet G, Vido K, Gaudu P, et al. Respiration Capacity of the Fermenting Bacterium *Lactococcus lactis* and Its Positive Effects on Growth and Survival. J Bacteriol. 2001 Aug;183(15):4509–16. doi:10.1128/JB.183.15.4509-4516.2001

24. Albenberg L, Esipova TV, Judge CP, Bittinger K, Chen J, Laughlin A, et al. Correlation Between Intraluminal Oxygen Gradient and Radial Partitioning of Intestinal Microbiota. Gastroenterology. 2014 Nov;147(5):1055-1063.e8. doi:10.1053/j.gastro.2014.07.020

25. Andres J, Bertin PN. The microbial genomics of arsenic. Danchin A, editor. FEMS Microbiology Reviews. 2016 Mar;40(2):299–322. doi:10.1093/femsre/fuv050

26. Oh JH, Lin XB, Zhang S, Tollenaar SL, Özçam M, Dunphy C, et al. Prophages in Lactobacillus reuteri Are Associated with Fitness Trade-Offs but Can Increase Competitiveness in the Gut Ecosystem. Kivisaar M, editor. Appl Environ Microbiol. 2019 Dec 13;86(1):e01922-19. doi:10.1128/AEM.01922-19

27. Su MS, Schlicht S, Gänzle MG. Contribution of glutamate decarboxylase in Lactobacillus reuteri to acid resistance and persistence in sourdough fermentation. Microb Cell Fact. 2011 Dec;10(S1):S8. doi:10.1186/1475-2859-10-S1-S8

28. Wegmann U, MacKenzie DA, Zheng J, Goesmann A, Roos S, Swarbreck D, et al. The pan-genome of Lactobacillus reuteri strains originating from the pig gastrointestinal tract. BMC Genomics. 2015 Dec;16(1):1023. doi:10.1186/s12864-015-2216-7

29. Jiang YL, Jin H, Yang HB, Zhao RL, Wang S, Chen Y, et al. Defining the enzymatic pathway for polymorphic O-glycosylation of the pneumococcal serine-rich repeat protein PsrP. Journal of Biological Chemistry. 2017 Apr;292(15):6213–24. doi:10.1074/jbc.M116.770446

30. Schwab C, Walter J, Tannock GW, Vogel RF, Gänzle MG. Sucrose utilization and impact of sucrose on glycosyltransferase expression in Lactobacillus reuteri. Systematic and Applied Microbiology. 2007 Sep;30(6):433–43. doi:10.1016/j.syapm.2007.03.007

31. Walter J, Schwab C, Loach DM, Gänzle MG, Tannock GW. Glucosyltransferase A (GtfA) and inulosucrase (Inu) of Lactobacillus reuteri TMW1.106 contribute to cell aggregation, in vitro biofilm formation, and colonization of the mouse gastrointestinal tract. Microbiology. 2008 Jan 1;154(1):72–80. doi:10.1099/mic.0.2007/010637-0

32. Engevik MA, Danhof HA, Shrestha R, Chang-Graham AL, Hyser JM, Haag AM, et al. Reuterin disrupts *Clostridioides difficile* metabolism and pathogenicity through reactive oxygen species generation. Gut Microbes. 2020 Nov 9;12(1):1795388. doi:10.1080/19490976.2020.1795388

33. Zhang Z, Wang K, Oh JH, Zhang S, Van Pijkeren JP, Cheng CC, et al. A Phylogenetic View on the Role of Glycerol for Growth Enhancement and Reuterin Formation in Limosilactobacillus reuteri. Front Microbiol. 2020 Dec 21;11:601422. doi:10.3389/fmicb.2020.601422

34. Büttner H, Perbandt M, Kohler T, Kikhney A, Wolters M, Christner M, et al. A Giant Extracellular Matrix Binding Protein of *Staphylococcus epidermidis* Binds Surface-

Immobilized Fibronectin via a Novel Mechanism. Msadek T, editor. mBio. 2020 Oct 27;11(5):e01612-20. doi:10.1128/mBio.01612-20

35. Sleator RD, Hill C. Bacterial osmoadaptation: the role of osmolytes in bacterial stress and virulence. FEMS Microbiol Rev. 2002 Mar;26(1):49–71. doi:10.1111/j.1574-6976.2002.tb00598.x

36. Walter J, Heng NCK, Hammes WP, Loach DM, Tannock GW, Hertel C. Identification of *Lactobacillus reuteri* Genes Specifically Induced in the Mouse Gastrointestinal Tract. Appl Environ Microbiol. 2003 Apr;69(4):2044–51. doi:10.1128/AEM.69.4.2044-2051.2003

37. Chaillou S, Postma PW, Pouwels PH. Functional Expression in *Lactobacillus plantarum* of *xylP* Encoding the Isoprimeverose Transporter of *Lactobacillus pentosus*. J Bacteriol. 1998 Aug;180(15):4011–4. doi:10.1128/JB.180.15.4011-4014.1998

38. Roos S, Aleljung P, Robert N, Lee B, WadstrÃ¶m T, Lindberg M, et al. A collagen binding protein from *Lactobacillus reuteri* is part of an ABC transporter system? FEMS Microbiology Letters. 1996 Oct;144(1):33–8. doi:10.1111/j.1574-6968.1996.tb08505.x

39. MacKenzie DA, Jeffers F, Parker ML, Vibert-Vallet A, Bongaerts RJ, Roos S, et al. Strain-specific diversity of mucus-binding proteins in the adhesion and aggregation properties of Lactobacillus reuteri. Microbiology. 2010 Nov 1;156(11):3368–78. doi:10.1099/mic.0.043265-0

40. Roos S, Jonsson H. A high-molecular-mass cell-surface protein from Lactobacillus reuteri 1063 adheres to mucus components.

41. Jensen H, Roos S, Jonsson H, Rud I, Grimmer S, Van Pijkeren JP, et al. Role of Lactobacillus reuteri cell and mucus-binding protein A (CmbA) in adhesion to intestinal epithelial cells and mucus in vitro. Microbiology. 2014 Apr 1;160(4):671–81. doi:10.1099/mic.0.073551-0

42. Walter J, Chagnaud P, Tannock GW, Loach DM, Dal Bello F, Jenkinson HF, et al. A High-Molecular-Mass Surface Protein (Lsp) and Methionine Sulfoxide Reductase B (MsrB) Contribute to the Ecological Performance of *Lactobacillus reuteri* in the Murine Gut. Appl Environ Microbiol. 2005 Feb;71(2):979–86. doi:10.1128/AEM.71.2.979-986.2005

43. Sequeira S, Kavanaugh D, MacKenzie DA, Šuligoj T, Walpole S, Leclaire C, et al. Structural basis for the role of serine-rich repeat proteins from *Lactobacillus reuteri* in gut microbe–host interactions. Proc Natl Acad Sci USA. 2018 Mar 20;115(12). doi:10.1073/pnas.1715016115

44. Gutiérrez-García K, Aumiller K, Dodge R, Obadia B, Deng A, Agrawal S, et al. A conserved bacterial genetic basis for commensal-host specificity. Science. 2024 Dec 6;386(6726):1117–22. doi:10.1126/science.adp7748

45. Patel S, Mathivanan N, Goyal A. Bacterial adhesins, the pathogenic weapons to trick host defense arsenal. Biomedicine & Pharmacotherapy. 2017 Sep;93:763–71. doi:10.1016/j.biopha.2017.06.102

46. Favrot L, Blanchard JS, Vergnolle O. Bacterial GCN5-Related *N* -Acetyltransferases: From Resistance to Regulation. Biochemistry. 2016 Feb 23;55(7):989–1002. doi:10.1021/acs.biochem.5b01269

47. Taranto MP, Vera JL, Hugenholtz J, De Valdez GF, Sesma F. *Lactobacillus reuteri* CRL1098 Produces Cobalamin. J Bacteriol. 2003 Sep 15;185(18):5643–7. doi:10.1128/JB.185.18.5643-5647.2003

48. Haridas M, Abdelraheem EMM, Hanefeld U. 2-Deoxy-d-ribose-5-phosphate aldolase (DERA): applications and modifications. Appl Microbiol Biotechnol. 2018 Dec;102(23):9959–71. doi:10.1007/s00253-018-9392-8

49. Papadimitriou K, Alegría Á, Bron PA, De Angelis M, Gobbetti M, Kleerebezem M, et al. Stress Physiology of Lactic Acid Bacteria. Microbiol Mol Biol Rev. 2016 Sep;80(3):837–90. doi:10.1128/MMBR.00076-15

50. Sychantha D, Brott AS, Jones CS, Clarke AJ. Mechanistic Pathways for Peptidoglycan O-Acetylation and De-O-Acetylation. Front Microbiol. 2018 Oct 1;9:2332. doi:10.3389/fmicb.2018.02332

51. Chen C, Li X, Yue L, Jing X, Yang Y, Xu Y, et al. Purification and characterization of lysozyme from Chinese Lueyang black-bone Silky fowl egg white. Preparative Biochemistry and Biotechnology. 2019 Mar 16;49(3):215–21. doi:10.1080/10826068.2018.1476887

52. Knight J, Deora R, Assimos DG, Holmes RP. The genetic composition of Oxalobacter formigenes and its relationship to colonization and calcium oxalate stone disease. Urolithiasis. 2013 Jun;41(3):187–96. doi:10.1007/s00240-013-0566-7

53. Kullin B, Tannock GW, Loach DM, Kimura K, Abratt VR, Reid SJ. A functional analysis of the formyl-coenzyme A (*frc*) gene from *Lactobacillus reuteri* 100-23C. J Appl Microbiol. 2014 Jun;116(6):1657–67. doi:10.1111/jam.12500

54. Fagan RP, Fairweather NF. Clostridium difficile Has Two Parallel and Essential Sec Secretion Systems. Journal of Biological Chemistry. 2011 Aug;286(31):27483–93. doi:10.1074/jbc.M111.263889

55. Huang M, Meng L, Fan M, Hu P, Bian Z. Effect of biofilm formation on virulence factor secretion via the general secretory pathway in Streptococcus mutans. Archives of Oral Biology. 2008 Dec;53(12):1179–85. doi:10.1016/j.archoralbio.2008.07.007

56. Wegkamp A, Mars AE, Faijes M, Molenaar D, De Vos RC, Klaus SM, et al. Physiological responses to folate overproduction in Lactobacillus plantarum WCFS1. Microb Cell Fact. 2010 Dec;9(1). doi:10.1186/1475-2859-9-100

57. Gazzaniga F, Stebbins R, Chang SZ, McPeek MA, Brenner C. Microbial NAD Metabolism: Lessons from Comparative Genomics. Microbiol Mol Biol Rev. 2009 Sep;73(3):529–41. doi:10.1128/mmbr.00042-08

58. Niehaus TD, Elbadawi-Sidhu M, De Crécy-Lagard V, Fiehn O, Hanson AD. Discovery of a widespread prokaryotic 5-oxoprolinase that was hiding in plain sight. Journal of Biological Chemistry. 2017 Sep;292(39):16360–7. doi:10.1074/jbc.m117.805028

59. Kunz DA, Ribbons DW, Chapman PJ. Metabolism of Allylglycine and *cis* -Crotylglycine by *Pseudomonas putida* (*arvilla*) mt-2 Harboring a TOL Plasmid. J Bacteriol. 1981 Oct;148(1):72–82. doi:10.1128/jb.148.1.72-82.1981

60. Salaemae W, Booker GW, Polyak SW. The Role of Biotin in Bacterial Physiology and Virulence: a Novel Antibiotic Target for *Mycobacterium tuberculosis*. Kudva IT, Bellaire BH, editors. Microbiol Spectr. 2016 Mar 25;4(2). doi:10.1128/microbiolspec.vmbf-0008-2015

61. Wu P, Zhu Q, Yang R, Mei Y, Chen Z, Liang Y. Differences in Acid Stress Response of Lacticaseibacillus paracasei Zhang Cultured from Solid-State Fermentation and Liquid-State Fermentation. Microorganisms. 2021 Sep 14;9(9):1951. doi:10.3390/microorganisms9091951

62. Marco ML, De Vries MC, Wels M, Molenaar D, Mangell P, Ahrne S, et al. Convergence in probiotic *Lactobacillus* gut-adaptive responses in humans and mice. The ISME Journal. 2010 Nov 1;4(11):1481–4. doi:10.1038/ismej.2010.61

63. Egervärn M, Roos S, Lindmark H. Identification and characterization of antibiotic resistance genes in*Lactobacillus reuteri*and*Lactobacillus plantarum*. Journal of Applied Microbiology. 2009 Nov;107(5):1658–68. doi:10.1111/j.1365-2672.2009.04352.x

64. Chang YC, Tsai CY, Lin CF, Wang YC, Wang IK, Chung TC. Characterization of tetracycline resistance lactobacilli isolated from swine intestines at western area of Taiwan. Anaerobe. 2011 Oct;17(5):239–45. doi:10.1016/j.anaerobe.2011.08.001

65. Lekagul A, Tangcharoensathien V, Yeung S. Patterns of antibiotic use in global pig production: A systematic review. Veterinary and Animal Science. 2019 Jun;7:100058. doi:10.1016/j.vas.2019.100058

66. Zavaleta V, Eyzaguirre J. Penicillium purpurogenum produces a highly stable endo-β-(1,4)-galactanase. Appl Biochem Biotechnol. 2016 Dec;180(7):1313–27. doi:10.1007/s12010-016-2169-6

67. Abedeera SM, Jayalath KS, Xie J, Rauff RM, Abeysirigunawardena SC. Pseudouridine Synthase RsuA Confers a Survival Advantage to Bacteria under Streptomycin Stress. Antibiotics. 2023 Sep 14;12(9):1447. doi:10.3390/antibiotics12091447

68. Karikó K, Buckstein M, Ni H, Weissman D. Suppression of RNA Recognition by Toll-like Receptors: The Impact of Nucleoside Modification and the Evolutionary Origin of RNA. Immunity. 2005 Aug;23(2):165–75. doi:10.1016/j.immuni.2005.06.008

69. Bansept F, Obeng N, Schulenburg H, Traulsen A. Modeling host-associating microbes under selection. The ISME Journal. 2021 Dec 1;15(12):3648–56. doi:10.1038/s41396-021-01039-0

70. Obeng N, Bansept F, Sieber M, Traulsen A, Schulenburg H. Evolution of Microbiota–Host Associations: The Microbe's Perspective. Trends in Microbiology. 2021 Sep;29(9):779–87. doi:10.1016/j.tim.2021.02.005

71. Sieber M, Traulsen A, Schulenburg H, Douglas AE. On the evolutionary origins of host–microbe associations. Proc Natl Acad Sci USA. 2021 Mar 2;118(9):e2016487118. doi:10.1073/pnas.2016487118

72. Lees JA, Galardini M, Bentley SD, Weiser JN, Corander J. pyseer: a comprehensive tool for microbial pangenome-wide association studies. Stegle O, editor. Bioinformatics. 2018 Dec 15;34(24):4310–2. doi:10.1093/bioinformatics/bty539

73. Valk LC, Luttik MAH, De Ram C, Pabst M, Van Den Broek M, Van Loosdrecht MCM, et al. A Novel D-Galacturonate Fermentation Pathway in Lactobacillus suebicus Links Initial Reactions of the Galacturonate-Isomerase Route With the Phosphoketolase Pathway. Front Microbiol. 2020 Jan 17;10. doi:10.3389/fmicb.2019.03027

74. Reverón I, Jiménez N, Curiel JA, Peñas E, López De Felipe F, De Las Rivas B, et al. Differential Gene Expression by Lactobacillus plantarum WCFS1 in Response to Phenolic Compounds Reveals New Genes Involved in Tannin Degradation. Dozois CM, editor. Appl Environ Microbiol. 2017 Apr;83(7). doi:10.1128/aem.03387-16

75. Jimï¿½nez N, Curiel JA, Reverï¿½n I, De Las Rivas B, Muï¿½oz R. Uncovering the Lactobacillus plantarum WCFS1 Gallate Decarboxylase Involved in Tannin Degradation. Appl Environ Microbiol. 2013 Jul 15;79(14):4253–63. doi:10.1128/aem.00840-13

76. Jones SA, Gibson T, Maltby RC, Chowdhury FZ, Stewart V, Cohen PS, et al. Anaerobic Respiration of Escherichia coli in the Mouse Intestine. Fang FC, editor. Infect Immun. 2011 Oct;79(10):4218–26. doi:10.1128/iai.05395-11

77. Buckel W, Thauer RK. Flavin-Based Electron Bifurcation, Ferredoxin, Flavodoxin, and Anaerobic Respiration With Protons (Ech) or NAD+ (Rnf) as Electron Acceptors: A Historical Review. Front Microbiol. 2018 Mar 14;9. doi:10.3389/fmicb.2018.00401

78. Juttukonda LJ, Green ER, Lonergan ZR, Heffern MC, Chang CJ, Skaar EP. *Acinetobacter baumannii*OxyR Regulates the Transcriptional Response to Hydrogen Peroxide. Whiteley M, editor. Infect Immun. 2019 Jan;87(1). doi:10.1128/iai.00413-18

79. Nikel PI, Fuhrer T, Chavarría M, Sánchez-Pascuala A, Sauer U, De Lorenzo V. Reconfiguration of metabolic fluxes in *Pseudomonas putida* as a response to sub-lethal oxidative stress. The ISME Journal. 2021 Jun 1;15(6):1751–66. doi:10.1038/s41396-020-00884-9

80. Nuñez MF, Pellicer MT, Badia J, Aguilar J, Baldoma L. Biochemical characterization of the 2-ketoacid reductases encoded by ycdW and yiaE genes in Escherichia coli. Biochem J. 2001 Mar 15;354(Pt 3):707–15. doi:10.1042/0264-6021:3540707 PubMed PMID: 11237876; PubMed Central PMCID: PMC1221703.

81. Ohura T, Kasuya KI, Doi Y. Cloning and Characterization of the Polyhydroxybutyrate Depolymerase Gene of *Pseudomonas stutzeri* and Analysis of the Function of Substrate-Binding Domains. Appl Environ Microbiol. 1999 Jan;65(1):189–97. doi:10.1128/aem.65.1.189-197.1999

82. Duar RM, Frese SA, Lin XB, Fernando SC, Burkey TE, Tasseva G, et al. Experimental Evaluation of Host Adaptation of Lactobacillus reuteri to Different Vertebrate Species.

Dudley EG, editor. Appl Environ Microbiol. 2017 Jun 15;83(12):e00132-17. doi:10.1128/AEM.00132-17

83. Ghosh TS, Arnoux J, O'Toole PW. Metagenomic analysis reveals distinct patterns of gut lactobacillus prevalence, abundance, and geographical variation in health and disease. Gut Microbes. 2020 Nov 9;12(1):1822729. doi:10.1080/19490976.2020.1822729

84. Pasolli E, De Filippis F, Mauriello IE, Cumbo F, Walsh AM, Leech J, et al. Large-scale genome-wide analysis links lactic acid bacteria from food with the gut microbiome. Nat Commun. 2020 May 25;11(1):2610. doi:10.1038/s41467-020-16438-8

85. Liu W, Wei Y, Xiang R, Dong B, Yang X. Lactic Acid Bacteria Exopolysaccharides Unveiling Multifaceted Insights from Structure to Application in Foods and Health Promotion. Foods. 2025 Feb 27;14(5):823. doi:10.3390/foods14050823

86. Wall T, Båth K, Britton RA, Jonsson H, Versalovic J, Roos S. The Early Response to Acid Shock in*Lactobacillus reuteri*Involves the ClpL Chaperone and a Putative Cell Wall-Altering Esterase. Appl Environ Microbiol. 2007 Jun 15;73(12):3924–35. doi:10.1128/aem.01502-06

87. Whitehead K, Versalovic J, Roos S, Britton RA. Genomic and Genetic Characterization of the Bile Stress Response of Probiotic*Lactobacillus reuteri*ATCC 55730. Appl Environ Microbiol. 2008 Mar 15;74(6):1812–9. doi:10.1128/aem.02259-07

88. Thomas CM, Hong T, van Pijkeren JP, Hemarajata P, Trinh DV, Hu W, et al. Histamine derived from probiotic Lactobacillus reuteri suppresses TNF via modulation of PKA and ERK signaling. PLoS One. 2012;7(2):e31951. doi:10.1371/journal.pone.0031951 PubMed PMID: 22384111; PubMed Central PMCID: PMC3285189.

89. Wüthrich D, Berthoud H, Wechsler D, Eugster E, Irmler S, Bruggmann R. The Histidine Decarboxylase Gene Cluster of Lactobacillus parabuchneri Was Gained by Horizontal Gene Transfer and Is Mobile within the Species. Front Microbiol. 2017 Feb 17;8. doi:10.3389/fmicb.2017.00218

90. Wu X, Han J, Gong G, Koffas MAG, Zha J. Wall teichoic acids: physiology and applications. FEMS Microbiology Reviews. 2021 Aug 17;45(4):fuaa064. doi:10.1093/femsre/fuaa064

91. Sengupta R, Altermann E, Anderson RC, McNabb WC, Moughan PJ, Roy NC. The Role of Cell Surface Architecture of Lactobacilli in Host-Microbe Interactions in the Gastrointestinal Tract. Mediators of Inflammation. 2013;2013:1–16. doi:10.1155/2013/237921

92. Bujdos D, O'Toole PW. *Ligilactobacillus*. In: Whitman WB, Rainey FA, Kämpfer P, Trujillo ME, DeVos P, Hedlund B, et al., editors. Bergey's Manual of Systematics of Archaea and Bacteria [Internet]. 1st ed. Wiley; 2025 [cited 2025 Aug 6]. p. 1–14. Available from: https://onlinelibrary.wiley.com/doi/10.1002/9781118960608.gbm02124 doi:10.1002/9781118960608.gbm02124

93. Kant R, Palva A, Von Ossowski I. An in silico pan-genomic probe for the molecular traits behind Lactobacillus ruminis gut autochthony. Moreno-Hagelsieb G, editor. PLoS ONE. 2017 Apr 17;12(4):e0175541. doi:10.1371/journal.pone.0175541

94. Wang S, Yang B, Ross RP, Stanton C, Zhao J, Zhang H, et al. Comparative Genomics Analysis of Lactobacillus ruminis from Different Niches. Genes. 2020 Jan 8;11(1):70. doi:10.3390/genes11010070

95. Wang S, Yang B, Ross RP, Stanton C, Zhao J, Zhang H, et al. Comparative Genomics Analysis of Lactobacillus ruminis from Different Niches. Genes. 2020 Jan 8;11(1):70. doi:10.3390/genes11010070

96. Yu X, Åvall-Jääskeläinen S, Koort J, Lindholm A, Rintahaka J, Von Ossowski I, et al. A Comparative Characterization of Different Host-sourced Lactobacillus ruminis Strains and Their Adhesive, Inhibitory, and Immunomodulating Functions. Front Microbiol. 2017 Apr 13;8. doi:10.3389/fmicb.2017.00657

97. Ghosh TS, Arnoux J, O'Toole PW. Metagenomic analysis reveals distinct patterns of gut lactobacillus prevalence, abundance, and geographical variation in health and disease. Gut Microbes. 2020 Nov 9;12(1):1822729. doi:10.1080/19490976.2020.1822729

98. Slamti L, Lereclus D. The oligopeptide ABC-importers are essential communication channels in Gram-positive bacteria. Research in Microbiology. 2019 Nov;170(8):338–44. doi:10.1016/j.resmic.2019.07.004

99. Wang S, Yang B, Ross RP, Stanton C, Zhao J, Zhang H, et al. Comparative Genomics Analysis of Lactobacillus ruminis from Different Niches. Genes. 2020 Jan 8;11(1):70. doi:10.3390/genes11010070

100. Sultan AR, Swierstra JW, Lemmens-den Toom NA, Snijders SV, Hansenová Maňásková S, Verbon A, et al. Production of Staphylococcal Complement Inhibitor (SCIN) and Other Immune Modulators during the Early Stages of Staphylococcus aureus Biofilm Formation in a Mammalian Cell Culture Medium. Freitag NE, editor. Infect Immun. 2018 Aug;86(8):e00352-18. doi:10.1128/IAI.00352-18

101. O' Donnell MM, Harris HMB, Lynch DB, Ross RP, O'Toole PW. Lactobacillus ruminis strains cluster according to their mammalian gut source. BMC Microbiol. 2015 Dec;15(1):80. doi:10.1186/s12866-015-0403-y

102. O' Donnell MM, Forde BM, Neville B, Ross PR, O' Toole PW. Carbohydrate catabolic flexibility in the mammalian intestinal commensal Lactobacillus ruminis revealed by fermentation studies aligned to genome annotations. Microb Cell Fact. 2011 Dec;10(S1):S12. doi:10.1186/1475-2859-10-S1-S12

103. Liu X, Mortensen UH, Workman M. Expression and functional studies of genes involved in transport and metabolism of glycerol in Pachysolen tannophilus. Microb Cell Fact. 2013 Dec;12(1):27. doi:10.1186/1475-2859-12-27

104. Moreno R, Yuste L, Rojo F. The acetoin assimilation pathway of *PSEUDOMONAS PUTIDA* KT2440 is regulated by overlapping global regulatory elements that respond to nutritional cues. Environmental Microbiology. 2023 Feb;25(2):515–31. doi:10.1111/1462-2920.16304

105. Xiao Z, Xu P. Acetoin Metabolism in Bacteria. Critical Reviews in Microbiology. 2007 Jan;33(2):127–40. doi:10.1080/10408410701364604

106. O' Donnell MM, Harris HMB, Lynch DB, Ross RP, O'Toole PW. Lactobacillus ruminis strains cluster according to their mammalian gut source. BMC Microbiol. 2015 Dec;15(1):80. doi:10.1186/s12866-015-0403-y

107. Yang H, Zhu Y, Chen X, Li X, Ye S, Zhang R. Structure of a prokaryotic SEFIR domain reveals two novel SEFIR-SEFIR interaction modes. Journal of Structural Biology. 2018 Aug;203(2):81–9. doi:10.1016/j.jsb.2018.03.005

108. Forde BM, Neville BA, O' Donnell MM, Riboulet-Bisson E, Claesson MJ, Coghlan A, et al. Genome sequences and comparative genomics of two Lactobacillus ruminis strains from the bovine and human intestinal tracts. Microb Cell Fact. 2011 Dec;10(S1):S13. doi:10.1186/1475-2859-10-S1-S13

109. Wang R, Jiang L, Zhang M, Zhao L, Hao Y, Guo H, et al. The Adhesion of Lactobacillus salivarius REN to a Human Intestinal Epithelial Cell Line Requires S-layer Proteins. Sci Rep. 2017 Mar 10;7(1):44029. doi:10.1038/srep44029

110. Vaval Taylor DM, Xayarath B, Freitag NE. Two Permeases Associated with the Multifunctional CtaP Cysteine Transport System in Listeria monocytogenes Play Distinct Roles in Pathogenesis. Brissette CA, editor. Microbiol Spectr. 2023 Jun 15;11(3):e03317-22. doi:10.1128/spectrum.03317-22

111. Xayarath B, Alonzo F, Freitag NE. Identification of a Peptide-Pheromone that Enhances Listeria monocytogenes Escape from Host Cell Vacuoles. O'Riordan M, editor. PLoS Pathog. 2015 Mar 30;11(3):e1004707. doi:10.1371/journal.ppat.1004707

112. Casiano-Colón A, Marquis RE. Role of the arginine deiminase system in protecting oral bacteria and an enzymatic basis for acid tolerance. Appl Environ Microbiol. 1988 Jun;54(6):1318–24. doi:10.1128/aem.54.6.1318-1324.1988

113. Sionek B, Szydłowska A, Trząskowska M, Kołożyn-Krajewska D. The Impact of Physicochemical Conditions on Lactic Acid Bacteria Survival in Food Products. Fermentation. 2024 Jun 4;10(6):298. doi:10.3390/fermentation10060298

114. Braga JD, Thongngam M, Kumrungsee T. Gamma-aminobutyric acid as a potential postbiotic mediator in the gut–brain axis. npj Sci Food. 2024 Apr 2;8(1):16. doi:10.1038/s41538-024-00253-2

115. Su MS, Schlicht S, Gänzle MG. Contribution of glutamate decarboxylase in Lactobacillus reuteri to acid resistance and persistence in sourdough fermentation. Microb Cell Fact. 2011 Dec;10(S1):S8. doi:10.1186/1475-2859-10-S1-S8

116. Focarelli F, Giachino A, Waldron KJ. Copper microenvironments in the human body define patterns of copper adaptation in pathogenic bacteria. Hiller NL, editor. PLoS Pathog. 2022 Jul 21;18(7):e1010617. doi:10.1371/journal.ppat.1010617

117. Hill GM, Cromwell GL, Crenshaw TD, Dove CR, Ewan RC, Knabe DA, et al. Growth promotion effects and plasma changes from feeding high dietary concentrations of zinc and copper to weanling pigs (regional study). Journal of Animal Science. 2000;78(4):1010. doi:10.2527/2000.7841010x

118. Fernandez M, Thompson J, Calle A. Novel feed additive delivers antimicrobial copper and influences fecal microbiota in pigs. Yang X, editor. Microbiol Spectr. 2024 Jun 4;12(6):e04280-23. doi:10.1128/spectrum.04280-23

119. Focarelli F, Giachino A, Waldron KJ. Copper microenvironments in the human body define patterns of copper adaptation in pathogenic bacteria. Hiller NL, editor. PLoS Pathog. 2022 Jul 21;18(7):e1010617. doi:10.1371/journal.ppat.1010617

120. Seaver LC, Imlay JA. Alkyl Hydroperoxide Reductase Is the Primary Scavenger of Endogenous Hydrogen Peroxide in *Escherichia coli*. J Bacteriol. 2001 Dec 15;183(24):7173–81. doi:10.1128/JB.183.24.7173-7181.2001

121. Gaballa A, Newton GL, Antelmann H, Parsonage D, Upton H, Rawat M, et al. Biosynthesis and functions of bacillithiol, a major low-molecular-weight thiol in Bacilli. Proc Natl Acad Sci USA. 2010 Apr 6;107(14):6482–6. doi:10.1073/pnas.1000928107

122. Zhang X, Xu P, Yu B. Chemical Synthesis of a Colanic Acid Hexasaccharide. Org Lett. 2022 Oct 28;24(42):7779–83. doi:10.1021/acs.orglett.2c03116

123. Wang Y, Xu X, Chen H, Yang F, Xu B, Wang K, et al. Assessment of beneficial effects and identification of host adaptation-associated genes of Ligilactobacillus salivarius isolated from badgers. BMC Genomics. 2023 Sep 7;24(1). doi:10.1186/s12864-023-09623-8

124. Xin WG, Li XD, Lin YC, Jiang YH, Xu MY, Zhang QL, et al. Whole genome analysis of host-associated lactobacillus salivarius and the effects on hepatic antioxidant enzymes and gut microorganisms of Sinocyclocheilus grahami. Front Microbiol. 2022 Oct 27;13. doi:10.3389/fmicb.2022.1014970

125. Iyer N, Williams MA, O'Callaghan AA, Dempsey E, Cabrera-Rubio R, Raverdeau M, et al. Lactobacillus salivarius UCC118™ Dampens Inflammation and Promotes Microbiota Recovery to Provide Therapeutic Benefit in a DSS-Induced Colitis Model. Microorganisms. 2022 Jul 9;10(7):1383. doi:10.3390/microorganisms10071383

126. Yang Y, Song X, Wang G, Xia Y, Xiong Z, Ai L. Understanding Ligilactobacillus salivarius from Probiotic Properties to Omics Technology: A Review. Foods. 2024 Mar 15;13(6):895. doi:10.3390/foods13060895

127. Lee JY, Han GG, Kim EB, Choi YJ. Comparative genomics of Lactobacillus salivarius strains focusing on their host adaptation. Microbiological Research. 2017 Dec;205:48–58. doi:10.1016/j.micres.2017.08.008

128. Hill C. Poles Apart: Where and How Cells Construct Nisin. mBio. 2020 Dec 22;11(6):e02991-20. doi:10.1128/mBio.02991-20

129. Sevillano E, Peña N, Lafuente I, Cintas LM, Muñoz-Atienza E, Hernández PE, et al. Nisin S, a Novel Nisin Variant Produced by Ligilactobacillus salivarius P1CEA3. IJMS. 2023 Apr 6;24(7):6813. doi:10.3390/ijms24076813

130. Morrison MJ, Imperiali B. The Renaissance of Bacillosamine and Its Derivatives: Pathway Characterization and Implications in Pathogenicity. Biochemistry. 2014 Feb 4;53(4):624–38. doi:10.1021/bi401546r

131. Larsen JC, Szymanski C, Guerry P. N-Linked Protein Glycosylation Is Required for Full Competence in *Campylobacter jejuni* 81-176. J Bacteriol. 2004 Oct;186(19):6508–14. doi:10.1128/jb.186.19.6508-6514.2004

132. Lin Y, Chen J, Zhou X, Li Y. Inhibition of*Streptococcus mutans*biofilm formation by strategies targeting the metabolism of exopolysaccharides. Critical Reviews in Microbiology. 2021 Sep 3;47(5):667–77. doi:10.1080/1040841x.2021.1915959

133. Jurášková D, Ribeiro SC, Silva CCG. Exopolysaccharides Produced by Lactic Acid Bacteria: From Biosynthesis to Health-Promoting Properties. Foods. 2022 Jan 8;11(2):156. doi:10.3390/foods11020156

134. Galloway-Peña JR, Liang X, Singh KV, Yadav P, Chang C, La Rosa SL, et al. The Identification and Functional Characterization of WxL Proteins from Enterococcus faecium Reveal Surface Proteins Involved in Extracellular Matrix Interactions. Zhulin IB, editor. J Bacteriol. 2015 Mar;197(5):882–92. doi:10.1128/jb.02288-14

135. Gerlach D, Guo Y, De Castro C, Kim SH, Schlatterer K, Xu FF, et al. Methicillin-resistant Staphylococcus aureus alters cell wall glycosylation to evade immunity. Nature. 2018 Nov 29;563(7733):705–9. doi:10.1038/s41586-018-0730-x

136. Gao J, Zhao X, Hu S, Huang Z, Hu M, Jin S, et al. Gut microbial DL-endopeptidase alleviates Crohn's disease via the NOD2 pathway. Cell Host & Microbe. 2022 Oct;30(10):1435-1449.e9. doi:10.1016/j.chom.2022.08.002

137. He J, Ruan W, Sun J, Wang F, Yan W. Functional Characterization of c-di-GMP Signaling-Related Genes in the Probiotic Lactobacillus acidophilus. Front Microbiol. 2018 Aug 29;9. doi:10.3389/fmicb.2018.01935

138. Girardi NM, Thoden JB, Holden HM. Misannotations of the genes encoding sugar *N*-formyltransferases. Protein Science. 2020 Apr;29(4):930–40. doi:10.1002/pro.3807

139. Gaballa A, Newton GL, Antelmann H, Parsonage D, Upton H, Rawat M, et al. Biosynthesis and functions of bacillithiol, a major low-molecular-weight thiol in Bacilli. Proc Natl Acad Sci USA. 2010 Apr 6;107(14):6482–6. doi:10.1073/pnas.1000928107

140. Newton GL, Koledin T, Gorovitz B, Rawat M, Fahey RC, Av-Gay Y. The Glycosyltransferase Gene Encoding the Enzyme Catalyzing the First Step of Mycothiol Biosynthesis (*mshA*). J Bacteriol. 2003 Jun;185(11):3476–9. doi:10.1128/jb.185.11.3476-3479.2003

141. Crawley AB, Henriksen JR, Barrangou R. CRISPRdisco: An Automated Pipeline for the Discovery and Analysis of CRISPR-Cas Systems. The CRISPR Journal. 2018 Apr;1(2):171–81. doi:10.1089/crispr.2017.0022

142. Lee HY, Yoon CK, Cho YJ, Lee JW, Lee KA, Lee WJ, et al. A mannose-sensing AraC-type transcriptional activator regulates cell–cell aggregation of Vibrio cholerae. npj Biofilms Microbiomes. 2022 Aug 20;8(1). doi:10.1038/s41522-022-00331-x

143. Denou E, Pridmore RD, Berger B, Panoff JM, Arigoni F, Brüssow H. Identification of Genes Associated with the Long-Gut-Persistence Phenotype of the Probiotic

Lactobacillus johnsonii Strain NCC533 Using a Combination of Genomics and Transcriptome Analysis. J Bacteriol. 2008 May;190(9):3161–8. doi:10.1128/jb.01637-07

144. Liveris ZJ, Kelley EH, Simmons E, Konczak K, Lutz Jr. MR, Ballicora M, et al. Synthesis and characterization of the N-succinyl-l,l-diaminopimelic acid desuccinylase (DapE) alternate substrate analog N,N-dimethyl-l,l-SDAP. Bioorganic & Medicinal Chemistry. 2023 Aug;91:117415. doi:10.1016/j.bmc.2023.117415

145. Seefeldt KE, Weimer BC. Diversity of Sulfur Compound Production in Lactic Acid Bacteria. Journal of Dairy Science. 2000 Dec;83(12):2740–6. doi:10.3168/jds.s0022-0302(00)75168-x

146. Dave UC, Kadeppagari RK. Alanine dehydrogenase and its applications – A review. Critical Reviews in Biotechnology. 2019 Jul 4;39(5):648–64. doi:10.1080/07388551.2019.1594153

147. Hudson AW, Barnes AJ, Bray AS, Ornelles DA, Zafar MA. Klebsiella pneumoniae L- Fucose Metabolism Promotes Gastrointestinal Colonization and Modulates Its Virulence Determinants. Raffatellu M, editor. Infect Immun. 2022 Oct 20;90(10). doi:10.1128/iai.00206-22

148. Osorio V, Sabater I Mezquita A, Balcázar JL. Comparative metagenomics reveals poultry and swine farming are hotspots for multidrug and tetracycline resistance. Environmental Pollution. 2023 Apr;322:121239. doi:10.1016/j.envpol.2023.121239

149. Casiano-Colón A, Marquis RE. Role of the arginine deiminase system in protecting oral bacteria and an enzymatic basis for acid tolerance. Appl Environ Microbiol. 1988 Jun;54(6):1318–24. doi:10.1128/aem.54.6.1318-1324.1988

150. Walter J, Loach DM, Alqumber M, Rockel C, Hermann C, Pfitzenmaier M, et al. D-Alanyl ester depletion of teichoic acids in *Lactobacillus reuteri* 100-23 results in impaired colonization of the mouse gastrointestinal tract. Environmental Microbiology. 2007 Jul;9(7):1750–60. doi:10.1111/j.1462-2920.2007.01292.x

151. Del Rio B, Alvarez-Sieiro P, Redruello B, Martin MC, Fernandez M, Ladero V, et al. Lactobacillus rossiae strain isolated from sourdough produces putrescine from arginine. Sci Rep. 2018 Mar 5;8(1). doi:10.1038/s41598-018-22309-6

152. Lee JY, Han GG, Kim EB, Choi YJ. Comparative genomics of Lactobacillus salivarius strains focusing on their host adaptation. Microbiological Research. 2017 Dec;205:48–58. doi:10.1016/j.micres.2017.08.008

153. Xu F, Hu XJ, Singh W, Geng W, Tikhonova IG, Lin J. The complex structure of bile salt hydrolase from Lactobacillus salivarius reveals the structural basis of substrate specificity. Sci Rep. 2019 Aug 27;9(1). doi:10.1038/s41598-019-48850-6

154. Lukomski S, Bachert BA, Squeglia F, Berisio R. Collagen-like proteins of pathogenic streptococci. Molecular Microbiology. 2017 Mar;103(6):919–30. doi:10.1111/mmi.13604

155. Lee JY, Han GG, Kim EB, Choi YJ. Comparative genomics of Lactobacillus salivarius strains focusing on their host adaptation. Microbiological Research. 2017 Dec;205:48–58. doi:10.1016/j.micres.2017.08.008

156. Hertzberger R, Arents J, Dekker HL, Pridmore RD, Gysler C, Kleerebezem M, et al. H_2O_2 Production in Species of the Lactobacillus acidophilus Group: a Central Role for a Novel NADH-Dependent Flavin Reductase. Macfarlane GT, editor. Appl Environ Microbiol. 2014 Apr;80(7):2229–39. doi:10.1128/aem.04272-13

157. Pircalabioru G, Aviello G, Kubica M, Zhdanov A, Paclet MH, Brennan L, et al. Defensive Mutualism Rescues NADPH Oxidase Inactivation in Gut Infection. Cell Host & Microbe. 2016 May;19(5):651–63. doi:10.1016/j.chom.2016.04.007

158. Hashimoto R, Nishiyama K, Namai F, Suzuki K, Sakuma T, Fukuda I, et al. Milk sialyl-oligosaccharides mediate the early colonization of gut commensal microbes in piglets. Microbiome. 2025 May 24;13(1). doi:10.1186/s40168-025-02129-3

159. Kentache T, Thabault L, Peracchi A, Frédérick R, Bommer GT, Van Schaftingen E. The putative *Escherichia coli* dehydrogenase YjhC metabolises two dehydrated forms of N-acetylneuraminate produced by some sialidases. Bioscience Reports. 2020 Jun 26;40(6). doi:10.1042/bsr20200927

160. De Souza De Azevedo PO, De Medeiros Oliveira M, Kuniyoshi TM, Matajira CEC, Frota EG, Dias M, et al. Phenotypic and genomic characterization of bacteriocin-producing lactic acid bacteria with probiotic and biotechnological potential for pathogen control in animal production. New Biotechnology. 2025 Sep;88:114–31. doi:10.1016/j.nbt.2025.04.008

161. Dobson A, Cotter PD, Ross RP, Hill C. Bacteriocin Production: a Probiotic Trait? Appl Environ Microbiol. 2012 Jan;78(1):1–6. doi:10.1128/aem.05576-11

162. Pan M, Hidalgo-Cantabrana C, Barrangou R. Host and body site-specific adaptation of Lactobacillus crispatus genomes. NAR Genomics and Bioinformatics. 2020 Mar 1;2(1):lqaa001. doi:10.1093/nargab/lqaa001

163. Santarelli G, Sanguinetti M, Delogu G, De Maio F. Comparative genomic analysis of bacteriocin genes in Lactobacillus crispatus strains. Sci Rep. 2025 Jul 1;15(1):20798. doi:10.1038/s41598-025-08321-7

164. Van Der Veer C, Hertzberger RY, Bruisten SM, Tytgat HLP, Swanenburg J, De Kat Angelino-Bart A, et al. Comparative genomics of human Lactobacillus crispatus isolates reveals genes for glycosylation and glycogen degradation: implications for in vivo dominance of the vaginal microbiota. Microbiome. 2019 Dec;7(1):49. doi:10.1186/s40168-019-0667-9

165. Zhang Q, Zhang L, Ross P, Zhao J, Zhang H, Chen W. Comparative Genomics of Lactobacillus crispatus from the Gut and Vagina Reveals Genetic Diversity and Lifestyle Adaptation. Genes. 2020 Mar 27;11(4):360. doi:10.3390/genes11040360

166. Pan M, Hidalgo-Cantabrana C, Barrangou R. Host and body site-specific adaptation of Lactobacillus crispatus genomes. NAR Genomics and Bioinformatics. 2020 Mar 1;2(1):lqaa001. doi:10.1093/nargab/lqaa001

167. Glowacki RWP, Pudlo NA, Tuncil Y, Luis AS, Sajjakulnukit P, Terekhov AI, et al. A Ribose-Scavenging System Confers Colonization Fitness on the Human Gut Symbiont Bacteroides thetaiotaomicron in a Diet-Specific Manner. Cell Host & Microbe. 2020 Jan;27(1):79-92.e9. doi:10.1016/j.chom.2019.11.009

168. Russell WM, Klaenhammer TR. Identification and Cloning of *gusA* , Encoding a New β-Glucuronidase from *Lactobacillus gasseri* ADH. Appl Environ Microbiol. 2001 Mar;67(3):1253–61. doi:10.1128/AEM.67.3.1253-1261.2001

169. Vogel-Scheel J, Alpert C, Engst W, Loh G, Blaut M. Requirement of Purine and Pyrimidine Synthesis for Colonization of the Mouse Intestine by *Escherichia coli*. Appl Environ Microbiol. 2010 Aug;76(15):5181–7. doi:10.1128/AEM.00242-10

170. Goncheva MI, Chin D, Heinrichs DE. Nucleotide biosynthesis: the base of bacterial pathogenesis. Trends in Microbiology. 2022 Aug;30(8):793–804. doi:10.1016/j.tim.2021.12.007

171. Tang Y, Underwood A, Gielbert A, Woodward MJ, Petrovska L. Metaproteomics Analysis Reveals the Adaptation Process for the Chicken Gut Microbiota. Appl Environ Microbiol. 2014 Jan 15;80(2):478–85. doi:10.1128/AEM.02472-13

172. Xu W, Yang W, Wang Y, Wang M, Zhang M. Structural and biochemical analyses of β-N-acetylhexosaminidase Am0868 from Akkermansia muciniphila involved in mucin degradation. Biochemical and Biophysical Research Communications. 2020 Sep;529(4):876–81. doi:10.1016/j.bbrc.2020.06.116

173. Wang H, Li S, Fang S, Yang X, Feng J. Betaine Improves Intestinal Functions by Enhancing Digestive Enzymes, Ameliorating Intestinal Morphology, and Enriching Intestinal Microbiota in High-salt stressed Rats. Nutrients. 2018 Jul 16;10(7):907. doi:10.3390/nu10070907

174. Luis AS, Hansson GC. Intestinal mucus and their glycans: A habitat for thriving microbiota. Cell Host & Microbe. 2023 Jul;31(7):1087–100. doi:10.1016/j.chom.2023.05.026

175. Chen Y, Gu J, Ashworth G, Wang Z, Zhang Z, Dong C. Crystal structure of the lipopolysaccharide outer core galactosyltransferase WaaB involved in pathogenic bacterial invasion of host cells. Front Microbiol. 2023 Sep 22;14:1239537. doi:10.3389/fmicb.2023.1239537

176. Malde A, Gangaiah D, Chandrashekhar K, Pina-Mimbela R, Torrelles JB, Rajashekara G. Functional characterization of exopolyphosphatase/guanosine pentaphosphate phosphohydrolase (PPX/GPPA) of *Campylobacter jejuni*. Virulence. 2014 May 15;5(4):521–33. doi:10.4161/viru.28311

177. Zhang J, Li L, Zhang T, Zhong J. Characterization of a novel type of glycogen-degrading amylopullulanase from Lactobacillus crispatus. Appl Microbiol Biotechnol. 2022 Jun;106(11):4053–64. doi:10.1007/s00253-022-11975-2

178. Sun Z, Kong J, Hu S, Kong W, Lu W, Liu W. Characterization of a S-layer protein from Lactobacillus crispatus K313 and the domains responsible for binding to cell wall and

adherence to collagen. Appl Microbiol Biotechnol. 2013 Mar;97(5):1941–52. doi:10.1007/s00253-012-4044-x

179. Marcotte H, Ferrari S, Cesena C, Hammarstrom L, Morelli L, Pozzi G, et al. The aggregation-promoting factor of Lactobacillus crispatus M247 and its genetic locus. J Appl Microbiol. 2004 Oct;97(4):749–56. doi:10.1111/j.1365-2672.2004.02364.x

180. Li H, Zang Y, Wang C, Li H, Fan A, Han C, et al. The Interaction Between Microorganisms, Metabolites, and Immune System in the Female Genital Tract Microenvironment. Front Cell Infect Microbiol. 2020 Dec 23;10:609488. doi:10.3389/fcimb.2020.609488

181. Zhang Q, Zhang L, Ross P, Zhao J, Zhang H, Chen W. Comparative Genomics of Lactobacillus crispatus from the Gut and Vagina Reveals Genetic Diversity and Lifestyle Adaptation. Genes. 2020 Mar 27;11(4):360. doi:10.3390/genes11040360

182. Tarracchini C, Lugli GA, Mancabelli L, Van Sinderen D, Turroni F, Ventura M, et al. Exploring the vitamin biosynthesis landscape of the human gut microbiota. Gilbert JA, editor. mSystems. 2024 Oct 22;9(10):e00929-24. doi:10.1128/msystems.00929-24

183. Dricot CEMK, Erreygers I, Cauwenberghs E, De Paz J, Spacova I, Verhoeven V, et al. Riboflavin for women's health and emerging microbiome strategies. npj Biofilms Microbiomes. 2024 Oct 18;10(1):107. doi:10.1038/s41522-024-00579-5

184. Dricot CEMK, Erreygers I, Cauwenberghs E, De Paz J, Spacova I, Verhoeven V, et al. Riboflavin for women's health and emerging microbiome strategies. npj Biofilms Microbiomes. 2024 Oct 18;10(1):107. doi:10.1038/s41522-024-00579-5

185. Selbach BP, Chung AH, Scott AD, George SJ, Cramer SP, Dos Santos PC. Fe-S Cluster Biogenesis in Gram-Positive Bacteria: SufU Is a Zinc-Dependent Sulfur Transfer Protein. Biochemistry. 2014 Jan 14;53(1):152–60. doi:10.1021/bi4011978

186. Zhang Q, Zhang L, Ross P, Zhao J, Zhang H, Chen W. Comparative Genomics of Lactobacillus crispatus from the Gut and Vagina Reveals Genetic Diversity and Lifestyle Adaptation. Genes. 2020 Mar 27;11(4):360. doi:10.3390/genes11040360

187. Serata M, Yasuda E, Sako T. Effect of superoxide dismutase and manganese on superoxide tolerance in *Lactobacillus casei* strain Shirota and analysis of multiple manganese transporters. Bioscience of Microbiota, Food and Health. 2018;37(2):31–8. doi:10.12938/bmfh.17-018

188. Haft DH, Varghese N. GlyGly-CTERM and Rhombosortase: A C-Terminal Protein Processing Signal in a Many-to-One Pairing with a Rhomboid Family Intramembrane Serine Protease. Donlin MJ, editor. PLoS ONE. 2011 Dec 14;6(12):e28886. doi:10.1371/journal.pone.0028886

189. He J, Ruan W, Sun J, Wang F, Yan W. Functional Characterization of c-di-GMP Signaling-Related Genes in the Probiotic Lactobacillus acidophilus. Front Microbiol. 2018 Aug 29;9:1935. doi:10.3389/fmicb.2018.01935

190. Cai XQ, Li RN, Ma Y, Chen XX, Wan LJ, Kan JS, et al. Comparative genomic analysis of Lactobacillus crispatus strains Lc31 and Lc83 with anti-cervical cancer potential by

complete genome sequencing. Cancer Adv. 2023;6(0):e23018. doi:10.53388/2023623018

191. Valasaki K, Staikou A, Theodorou LG, Charamopoulou V, Zacharaki P, Papamichael EM. Purification and kinetics of two novel thermophilic extracellular proteases from Lactobacillus helveticus, from kefir with possible biotechnological interest. Bioresource Technology. 2008 Sep;99(13):5804–13. doi:10.1016/j.biortech.2007.10.018

192. Singleton M, Isupov M, Littlechild J. X-ray structure of pyrrolidone carboxyl peptidase from the hyperthermophilic archaeon Thermococcus litoralis. Structure. 1999 Mar;7(3):237–44. doi:10.1016/S0969-2126(99)80034-3

193. Zoll S, Pätzold B, Schlag M, Götz F, Kalbacher H, Stehle T. Structural Basis of Cell Wall Cleavage by a Staphylococcal Autolysin. Ghosh P, editor. PLoS Pathog. 2010 Mar 12;6(3):e1000807. doi:10.1371/journal.ppat.1000807

194. Pan M, Hidalgo-Cantabrana C, Barrangou R. Host and body site-specific adaptation of Lactobacillus crispatus genomes. NAR Genomics and Bioinformatics. 2020 Mar 1;2(1):lqaa001. doi:10.1093/nargab/lqaa001

195. Naemi AO, Dey H, Kiran N, Sandvik ST, Slettemeås JS, Nesse LL, et al. NarAB Is an ABC-Type Transporter That Confers Resistance to the Polyether Ionophores Narasin, Salinomycin, and Maduramicin, but Not Monensin. Front Microbiol. 2020 Feb 4;11:104. doi:10.3389/fmicb.2020.00104

196. Chen J, Yan F, Kuttappan VA, Wedekind K, Vázquez-Añón M, Hancock D. Effects of bis-chelated copper in growth performance and gut health in broiler chickens subject to coccidiosis vaccination or coccidia challenge. Front Physiol. 2023 Feb 3;13:991318. doi:10.3389/fphys.2022.991318

197. Gutierrez C, Gordia S, Bonnassie S. Characterization of the osmotically inducible gene *osmE* of *Escherichia coli* K-12. Molecular Microbiology. 1995 May;16(3):553–63. doi:10.1111/j.1365-2958.1995.tb02418.x

198. Juttukonda LJ, Green ER, Lonergan ZR, Heffern MC, Chang CJ, Skaar EP. *Acinetobacter baumannii* OxyR Regulates the Transcriptional Response to Hydrogen Peroxide. Whiteley M, editor. Infect Immun. 2019 Jan;87(1):e00413-18. doi:10.1128/IAI.00413-18

199. Pan M, Hidalgo-Cantabrana C, Barrangou R. Host and body site-specific adaptation of Lactobacillus crispatus genomes. NAR Genomics and Bioinformatics. 2020 Mar 1;2(1):lqaa001. doi:10.1093/nargab/lqaa001

200. Richards PJ, Flaujac Lafontaine GM, Connerton PL, Liang L, Asiani K, Fish NM, et al. Galacto-Oligosaccharides Modulate the Juvenile Gut Microbiome and Innate Immunity To Improve Broiler Chicken Performance. Cleary DW, editor. mSystems. 2020 Feb 11;5(1):10.1128/msystems.00827-19. doi:10.1128/msystems.00827-19

201. Serata M, Yasuda E, Sako T. Effect of superoxide dismutase and manganese on superoxide tolerance in *Lactobacillus casei* strain Shirota and analysis of multiple manganese transporters. Bioscience of Microbiota, Food and Health. 2018;37(2):31–8. doi:10.12938/bmfh.17-018

202. Roos S, Karner F, Axelsson L, Jonsson H. Lactobacillus mucosae sp. nov., a new species with in vitro mucus-binding activity isolated from pig intestine. International Journal of Systematic and Evolutionary Microbiology. 2000 Jan 1;50(1):251–8. doi:10.1099/00207713-50-1-251

203. Jia Y, Yang B, Ross P, Stanton C, Zhang H, Zhao J, et al. Comparative Genomics Analysis of Lactobacillus mucosae from Different Niches. Genes. 2020 Jan 14;11(1):95. doi:10.3390/genes11010095

204. London LEE, Price NPJ, Ryan P, Wang L, Auty MAE, Fitzgerald GF, et al. Characterization of a bovine isolate *Lactobacillus mucosae* DPC 6426 which produces an exopolysaccharide composed predominantly of mannose residues. J Appl Microbiol. 2014 Aug;117(2):509–17. doi:10.1111/jam.12542

205. Ryan PM, Stolte EH, London LEE, Wells JM, Long SL, Joyce SA, et al. Lactobacillus mucosae DPC 6426 as a bile-modifying and immunomodulatory microbe. BMC Microbiol. 2019 Dec;19(1):33. doi:10.1186/s12866-019-1403-0

206. Jia Y, Yang B, Ross P, Stanton C, Zhang H, Zhao J, et al. Comparative Genomics Analysis of Lactobacillus mucosae from Different Niches. Genes. 2020 Jan 14;11(1):95. doi:10.3390/genes11010095

207. Jia Y, Yang B, Ross P, Stanton C, Zhang H, Zhao J, et al. Comparative Genomics Analysis of Lactobacillus mucosae from Different Niches. Genes. 2020 Jan 14;11(1):95. doi:10.3390/genes11010095

208. Walter J, Loach DM, Alqumber M, Rockel C, Hermann C, Pfitzenmaier M, et al. D-Alanyl ester depletion of teichoic acids in *Lactobacillus reuteri* 100-23 results in impaired colonization of the mouse gastrointestinal tract. Environmental Microbiology. 2007 Jul;9(7):1750–60. doi:10.1111/j.1462-2920.2007.01292.x

209. Watson AR, Füssel J, Veseli I, DeLongchamp JZ, Silva M, Trigodet F, et al. Metabolic independence drives gut microbial colonization and resilience in health and disease. Genome Biol. 2023 Apr 17;24(1):78. doi:10.1186/s13059-023-02924-x

210. Roager HM, Licht TR. Microbial tryptophan catabolites in health and disease. Nat Commun. 2018 Aug 17;9(1):3294. doi:10.1038/s41467-018-05470-4

211. Jia Y, Yang B, Ross P, Stanton C, Zhang H, Zhao J, et al. Comparative Genomics Analysis of Lactobacillus mucosae from Different Niches. Genes. 2020 Jan 14;11(1):95. doi:10.3390/genes11010095

212. Goh YJ, Klaenhammer TR. Insights into glycogen metabolism in Lactobacillus acidophilus: impact on carbohydrate metabolism, stress tolerance and gut retention. Microb Cell Fact. 2014 Dec;13(1):94. doi:10.1186/s12934-014-0094-3

213. Alquéres S, Meneses C, Rouws L, Rothballer M, Baldani I, Schmid M, et al. The Bacterial Superoxide Dismutase and Glutathione Reductase Are Crucial for Endophytic Colonization of Rice Roots by *Gluconacetobacter diazotrophicus* PAL5. MPMI. 2013 Aug;26(8):937–45. doi:10.1094/MPMI-12-12-0286-R

214. Lüscher B, Ahel I, Altmeyer M, Ashworth A, Bai P, Chang P, et al. ADP-ribosyltransferases, an update on function and nomenclature. The FEBS Journal. 2022 Dec;289(23):7399–410. doi:10.1111/febs.16142

215. Xiao Z, Xu P. Acetoin Metabolism in Bacteria. Critical Reviews in Microbiology. 2007 Jan;33(2):127–40. doi:10.1080/10408410701364604
